# The Incorporation of CBD into Biodegradable DL-Lactide/Glycolide Copolymers Creates a Persistent Antibacterial Environment: An In Vitro Study on *Streptococcus mutans* and *Staphylococcus aureus*

**DOI:** 10.3390/pharmaceutics17040463

**Published:** 2025-04-02

**Authors:** Ronit Vogt Sionov, Ahmad Siag, Emma Theresa Mersini, Natalya M. Kogan, Tatiana Alkhazov, Igor Koman, Praveen Rowlo, Vitaly Gutkin, Menachem Gross, Doron Steinberg

**Affiliations:** 1 Faculty of Dental Medicine, Institute of Biomedical and Oral Research (IBOR), The Hebrew University of Jerusalem, Ein Kerem Campus, Jerusalem 9112102, Israel; ahmadsi@hadassah.org.il (A.S.); emma.th.mersini@gmail.com (E.T.M.); praveen.rowlo@mail.huji.ac.il (P.R.); dorons@ekmd.huji.ac.il (D.S.); 2 Department of Otolaryngology-Head and Neck Surgery, Hadassah Medical Center, Jerusalem 9112102, Israel; drgrossm@hotmail.com; 3 Institute of Personalized and Translational Medicine, Department of Molecular Biology, Ariel University, Ariel 4070000, Israel; natalyak@ariel.ac.il (N.M.K.); tatianal@ariel.ac.il (T.A.); igorko@ariel.ac.il (I.K.); 4 Unit for Nano Characterization, The Harvey M. Krueger Family Center for Nanoscience and Nanotechnology, The Hebrew University of Jerusalem, Edmond J. Safra Campus, Jerusalem 9190401, Israel; vitalyg@savion.huji.ac.il

**Keywords:** cannabidiol, CBD, sustained-release device, PLGA scaffolds, antibacterial, antibiofilm, *Streptococcus mutans*, *Staphylococcus aureus*, IL-6, macrophages

## Abstract

**Background**: Cannabidiol (CBD) is a natural compound from the *Cannabis sativa* L. plant, which has anti-inflammatory, anti-nociceptive, neuroprotective, and antibacterial activities. **Objective**: The aim of this study was to develop a sustained-release device of CBD that can provide an antibacterial effect against the Gram-positive bacteria *Streptococcus mutans* and *Staphylococcus aureus* for extended periods of time. **Methods**: CBD was incorporated into the biodegradable PURASORB 5010 or PURASORB 7510 DL-lactide/glycolide polymers using either dimethylsulfoxide (DMSO) or acetone as the solvent, and the dried polymer scaffolds were exposed daily to a fresh culture of bacteria. The bacterial growth was determined daily by optical density, and the metabolic activity of biofilms was determined using the MTT assay. Biofilm formation on the polymer scaffolds was visualized by HR-SEM. Its anti-inflammatory effect was determined by measuring the IL-6 release from LPS-stimulated RAW 264.7 macrophages by ELISA. Cell cytotoxicity on normal Vero epithelial cells was determined by the MTT assay. The daily release of CBD was determined by gas chromatography–mass spectrometry (GC-MS). **Results**: PURASORB 5010/CBD scaffolds had antibacterial activity against *S. mutans* UA159, *S. aureus* ATCC25923, and a clinical isolate of a multidrug-resistant *S. aureus* (MDRSA CI-M) strain for the tested period of up to 17 days. PURASORB 7510/CBD scaffolds also had antibacterial activity, but overall, it was less effective than PURASORB 5010/CBD over time. The addition of PEG400 to the copolymers significantly increased the antibacterial activity of PURASORB 7510/CBD but not of PURASORB 5010/CBD. The daily release of CBD from the polymer scaffolds was sufficient to reduce the LPS-induced IL-6 secretion from RAW 264.7 macrophages, and importantly, it was not cytotoxic to either RAW 264.7 macrophages or Vero epithelial cells. The daily release of CBD was found to be between 1.12 and 9.43 µg/mL, which is far below the cytotoxic dose of 25 µg/mL. **Conclusions**: The incorporation of CBD into the biodegradable PURASORB 5010 can be used to prepare sustained-release devices for medical purposes where combined antibacterial and anti-inflammatory activities are desirable.

## 1. Introduction

The local delivery of drugs by sustained-release technology has the advantage of directly providing the medication to the target site for prolonged periods of time, resulting in smaller fluctuations in drug levels [1,2]. This requires lower doses of the drugs and less frequent dosages, which leads to higher patient compliance. Local delivery prevents the adverse effects of the systemic administration of the drug, avoids toxic effects on the gastrointestinal tract, and circumvents the first-pass metabolism in the liver [1,2].

Various approaches have been developed to obtain sustained-release systems, including hydrogels, microspheres, nanospheres, liposomes, electrospun fibers, microneedle patches, osmotic pumps, sustained-release varnishes (SRVs), and biodegradable polymeric implants [2,3,4,5,6,7,8,9,10,11]. The local application of drugs is particularly useful in the treatment of infections and inflammatory conditions. The use of sustained-release varnishes (SRVs) on medical devices to deliver antibacterial (e.g., chlorhexidine), antifungal (e.g., clotrimazole), and anti-inflammatory (e.g., mometasone) drugs has been extensively investigated [12,13,14,15,16].

Commonly used biodegradable materials include polylactide-co-glycolide (PLGA) and poly(L-lactide-co-ε-caprolactone) (PLCL) polymers, which slowly release the drug into the surroundings when the polymer degrades [17]. A major advantage of PLGA is that it breaks down into nontoxic compounds (primarily lactic acid and glycolic acid) that can be metabolized by the body without eliciting an inflammatory response [11,17]. Another advantage is that it does not need to be surgically removed [11]. PLGA copolymers have been approved for medical uses by the United States Food and Drug Administration (FDA) and the European Medicines Agency (EMA) [18,19]. Examples of the clinical use of PLGA include the steroid-releasing, biodegradable PROPEL and SINUVA sinus implants, which contain mometasone furoate and are used to reduce local inflammation in the nasal cavity [20], and the antibacterial Ethicon Polyglactin 910 Vicryl sutures, which contain triclosan to reduce bacterial infections caused by *Staphylococcus* spp. after surgery [21]. Polyglactin 910 is a copolymer composed of 90% glycolide and 10% L-lactide. Polyglactin 910 is also used as an abdominal woven mesh for hernia repair [22]. Adding antibacterial properties to the abdominal meshes is expected to reduce post-operative complications caused by infection [23]. Since bacterial infections are often associated with inflammation, it has been proposed that antibacterial and anti-inflammatory drugs should be combined to provide a better treatment outcome [24,25,26,27].

Cannabidiol (CBD) is a non-psychoactive compound of the plant *Cannabis sativa* L., which has numerous therapeutic applications due to its anti-inflammatory, anti-nociceptive, antispasmodic, and antibacterial activities [28,29,30,31]. Additionally, it has neuroprotective, bone-regenerative, and antioxidant effects [28,30,32]. These unique properties have made CBD an attractive drug for combined antibacterial and anti-inflammatory treatment. Its antibacterial activity is especially effective against Gram-positive bacteria (e.g., *Streptococcus* spp., *Staphylococcus* spp., *Clostridium* spp., *Enterococcus* spp.) with a minimum inhibitory concentration (MIC) of 1–5 µg/mL [29,33,34,35,36,37,38]. Most Gram-negative bacteria are usually resistant (MIC > 64 µg/mL) unless combined with other agents such as polymyxin B [36,38,39,40]. An exception is the Gram-negative *Neisseria gonorrhoeae* which is highly sensitive with an MIC of 0.25–1 µg/mL [36]. Also, the opportunistic vaginal Gram-variable pathogen *Gardnerella vaginalis* is susceptible with an MIC of 2.5 µg/mL [41]. A relatively high dose of CBD (5–10 µg/mL) is required to reduce the growth of the periodontal pathogen *Porphyromonas gingivalis*, while 1 µg/mL is sufficient to reduce *P. gingivalis*-induced pro-inflammatory cytokine secretion from human monocytes [42]. The addition of CBD to mouthwashes has been shown to reduce the bacterial burden in dental plaques [43]. Importantly, CBD is as effective against methicillin-resistant *S. aureus* (MRSA) as it is against methicillin-susceptible strains (MSSA) [37], and it can reduce the bacterial load of *S. aureus* in a porcine skin infection model [36]. So far, there is no evidence that prolonged exposure to CBD results in the appearance of CBD-resistant bacteria [36].

Several delivery systems for CBD have been tested to increase its bioavailability for systemic treatment, including nanoemulsions and lipid-based formulations [44,45,46,47,48]. A major limitation of the oral administration of CBD is its high first-pass metabolism in the liver, which leads to rapid inactivation [44,45,49]. Another limitation is its high affinity to serum albumin, which reduces the free CBD concentration in the blood [50]. Sublingual administration of CBD can increase bioavailability [51], while transdermal administration has limited bioavailability, as most of the CBD is retained in the outermost layers of the epidermis due to the stratum corneum barrier [44,49]. Although great efforts have been made to increase the bioavailability of CBD for the treatment of systemic diseases, we hypothesized that a sustained-release device would be preferable for local treatment at the site of infection. Such a system should deliver a low but effective dose of the active compound over an extended period of time.

The aim of this research was to formulate a sustained-release delivery system for CBD that can be used locally to prevent the growth of Gram-positive pathogens such as *S. aureus*, which is involved in infectious diseases [52], and *Streptococcus mutans*, which is involved in dental caries [53]. We present data showing that the incorporation of CBD into the PURASORB^®^ PDLG 5010 (50/50 DL-lactide/glycolide) and 7510 (75/25 DL-lactide/glycolide) polymers resulted in long-term antibacterial activity against MSSA, MRSA, and *S. mutans*, suggesting that this drug delivery system has potential applications for reducing bacterial burden at the infection site.

## 2. Materials and Methods

### 2.1. Materials

PURASORB^®^ PDLG 5010 (50/50 DL-lactide/glycolide copolymer; inherent viscosity 1.05 dL/g) and PURASORB^®^ PDLG 7510 (75/25 DL-lactide/glycolide copolymer; inherent viscosity 0.99 dL/g) were a kind gift from the Corbion Company (Purac Biochem BV, Gorinchem, the Netherlands). Cannabidiol (CBD) isolate (99.4% purity) was purchased from NCLABS (Prague, Czech Republic; see Certificate of Analysis). Cannabinoid profiling of the CBD showed the presence of 0.1% C4-CBD (cannabidibutol) and 0.3% CBDV (cannabidivarin), but no Δ^8^-THC (tetrahydrocannabinol) or Δ^9^-THC was detected.

### 2.2. Incorporation of CBD into PURASORB 5010 and PURASORB 7510

**Figure 1:**
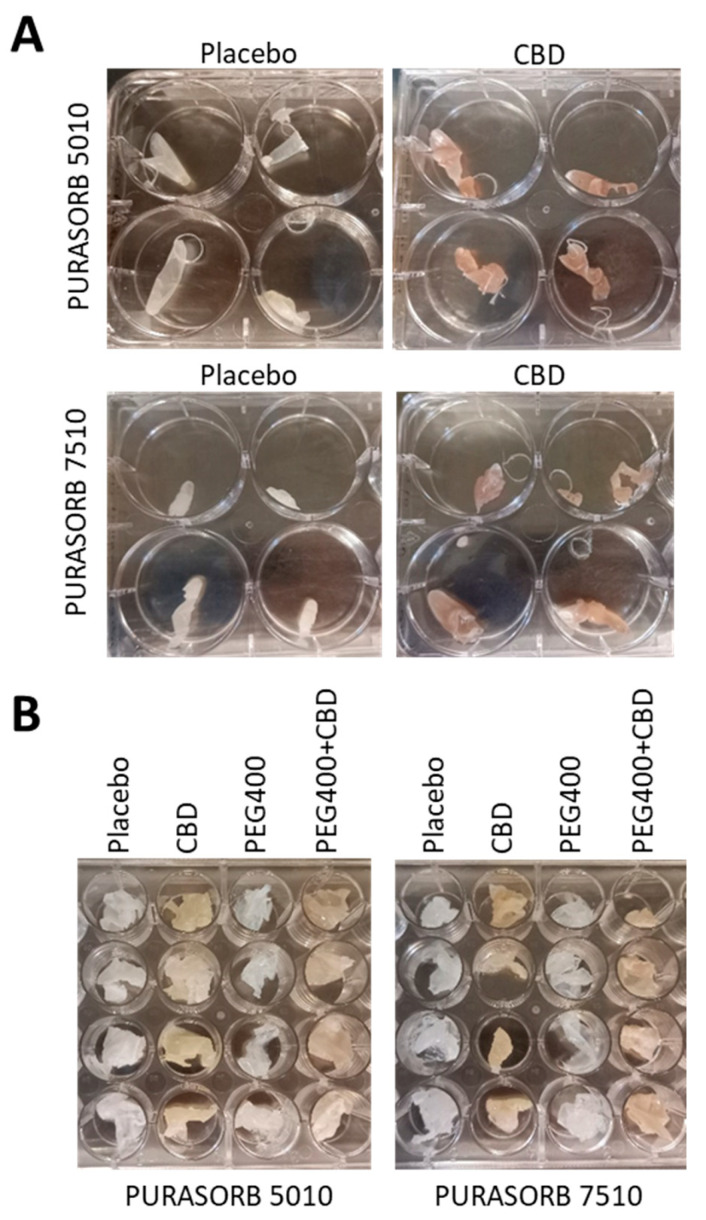
(**A**,**B**) Images of the PURASORB/CBD scaffolds formed using DMSO (**A**) or acetone (**B**) as the solvent. The scaffolds are flexible and can be made into different structures. The 5010 scaffolds were softer than the 7510 scaffolds, and the incorporation of CBD caused the scaffolds to become even softer.

Preliminary experiments were conducted to determine the optimal PURASORB/CBD ratio and to identify the solvents that dissolve both CBD and PURASORB without compromising CBD’s antibacterial activity. Two main protocols were then used to prepare the PURASORB/CBD scaffolds. In the first approach, 100 mg of PURASORB 5010 or PURASORB 7510 was allowed to dissolve in 500 µL dimethyl sulfoxide (DMSO) in Eppendorf tubes (Axygen brand products, Corning Inc., Reynosa, Mexico) overnight at room temperature with constant mixing. Thereafter, 50 mg CBD was added and mixed for 1 h at room temperature. The controls were placebo samples containing only the polymers. After the CBD was dissolved and thoroughly mixed with the polymers, the samples were frozen at −80 °C for 2 h. The tubes with perforated lids were then lyophilized for 14 days to ensure that all the DMSO had evaporated. The scaffolds were washed extensively with sterile double distilled water (DDW) to ensure that the residual DMSO was removed and then dried. The placebo scaffolds appeared white and firm, while the CBD-containing scaffolds were brownish and softer. Each scaffold was cut into four pieces (Figure 1A).

In the second approach, 100 mg of PURASORB 5010 or PURASORB 7510 was allowed to dissolve in 2 mL acetone for 5 h at room temperature in a glass vial with constant mixing. Then, 50 mg CBD was added to each sample, which was immediately dissolved. After mixing thoroughly for 2–3 min, the solutions were rapidly poured into sterile glass beakers, which were swirled to rapidly evaporate the acetone to form a dry film on the glass surface. The films were washed extensively with sterile DDW to ensure the removal of residual acetone and then dried. Each scaffold was cut into four pieces (Figure 1B). Alternatively, 10 µL of polyethylene glycol-400 (PEG400; Sigma, St. Louis, MO, USA) was added to PUROSORB samples in acetone before the addition of CBD. Controls were placebo samples of the same preparations without CBD. The dried scaffolds were kept in the dark at room temperature and used in the experiments within one week.

### 2.3. Bacterial Strains and Growth Conditions

*Streptococcus mutans* UA159 (ATCC700610) was cultured in brain–heart infusion (BHI) broth (HiMedia Laboratories Pvt. Ltd., Mumbai, India) [34], while *Staphylococcus aureus* (ATCC25923) and a multidrug-resistant *S. aureus* clinical isolate (MDRSA CI-M) were cultured in tryptic soy broth (TSB) (HiMedia Laboratories Pvt. Ltd., Mumbai, India) [54]. Fresh bacterial cultures were prepared daily by inoculating 100 µL of a frozen bacterial stock in its respective growth medium (10 mL) for an overnight incubation at 37 °C in a humidified incubator supplemented with 5% CO_2_. The optical density (OD)_600nm_ of the overnight culture was measured in an Ultraspec 10 spectrophotometer (Amersham Biosciences, Freiburg im Breisgau, Germany) and then diluted in the appropriate growth medium to an OD_600nm_ of 0.1 prior to exposure to the scaffolds. This meant that the bacteria had reached the late-log phase/early stationary phase before dilution. The morphology of the bacteria was inspected under a light microscope using a ×100 lens. *S. mutans* appears as ovoid bacteria arranged in long chains, while *S. aureus* appears as round bacteria in doublets (diplococci) or clusters.

### 2.4. Determination of Bacterial Viability

The polymer scaffolds (37.5 ± 5.2 mg per sample) were exposed daily to 1 mL of a fresh bacterial culture at an OD_600nm_ of 0.1 in tissue-grade 24-well plates (Corning Incorporation, Kennebunk, ME, USA) in quadruplicates. After a 24 h incubation, the polymer scaffolds were transferred to new wells and re-exposed to 1 mL of a new, fresh bacterial culture (OD_600nm_ = 0.1) for another 24 h incubation. This was repeated daily. For the weekends, the incubation time was 48 h, with similar data as after a 24 h incubation. The turbidity of the bacterial culture after exposure to the polymer scaffolds was measured at an OD of 600 nm in a Multiskan SkyHigh microplate reader (Thermo Scientific, Life Technologies Holdings Pte Ltd., Singapore). Placebo samples and bacteria in empty wells served as controls. The medium exposed to polymer samples without bacteria served as a background control and had an OD similar to that of the medium alone.

### 2.5. Determination of Metabolically Active Biofilms

Biofilms formed at the well surface were washed gently with PBS, and the metabolic activity was determined by adding 500 µL of 0.5 mg/mL MTT (Sigma, St. Louis, MO, USA) in PBS to each well for a 2 h incubation at 37 °C [54]. At the end of the incubation period, the excess MTT solution was discarded and the purple-blue formazan precipitates formed in the biofilms were dissolved in 1 mL of DMSO. The OD at 570 nm was read in a Multiskan SkyHigh microplate reader (ThermoScientific, Life Technologies Holdings Pte Ltd., Singapore). Placebo samples and bacteria in empty wells served as controls. The metabolic activity of the biofilms was calculated against control samples without scaffolds which was set to 100%.

### 2.6. HR-SEM Imaging

Polymer scaffolds that had been repeatedly exposed to bacterial cultures were washed twice in DDW, fixed with 4% glutaraldehyde (Electron Microscopy Sciences, Hatfield, PA, USA) in DDW for 2 h, rewashed in DDW, and then allowed to dry [54]. The samples were then coated with iridium and visualized by an analytical high-resolution scanning electron microscope (HR-SEM) (Apreo 2 S LoVac, Thermo Scientific) at various magnifications. HR-SEM imaging was performed blind with numbered samples without knowing the sample identities.

### 2.7. Determination of IL-6 Secretion from RAW 264.7 Macrophages

To study whether the daily release of CBD from the scaffolds was sufficient to prevent the IL-6 secretion from macrophages, PURASORB 5010 and PURASORB 7510 scaffolds with or without CBD were exposed daily to 1 mL serum-free RPMI-1640 (Sigma) for a 24 h incubation at 37 °C in triplicates. The conditioned media were kept at −20 °C until used for the IL-6 assay. 1.5 × 10^5^ RAW 264.7 macrophages (ATCC TIB-7) were seeded in 200 µL of DMEM (Sigma) supplemented with 8% heat-inactivated fetal calf serum (Sigma), 2 mM L-glutamine, 1 mM sodium pyruvate, 100 U/mL penicillin, and 0.1 mg/mL streptomycin in tissue-grade 96-well plates (Corning Inc., Kennebunk, ME, USA) for an overnight incubation at 37 °C. On the following day, the medium was exchanged with 100 µL of serum-free RPMI-1640 medium, the scaffold-conditioned media, or RPMI-1640 with different concentrations of CBD (1–5 µg/mL). After a 30 min incubation at 37 °C in a humidified incubator supplemented with 5% CO_2_, 100 µL of serum-free RPMI-1640 medium containing 20 ng/mL lipopolysaccharide (LPS; Sigma) was added to obtain a final concentration of 10 ng/mL LPS. RPMI was added to control wells to measure baseline IL-6 production. The plates were incubated for 6 h at 37 °C. In total, 100 µL of the supernatant from each well was transferred to the ELISA plates (Nunc, Maxisorb immune plates, Thermo Scientific, Roskilde, Denmark) precoated with capture IL-6 antibodies from the murine IL-6 standard TMB developmental kit (Catalog# 900-T50, Peprotech, Cranbury, NJ, USA) for an overnight incubation on an orbital shaker at 4 °C. The next steps of the ELISA were performed according to the manufacturer’s instruction using 3,3′,5,5′ tetramethylbenzidine (TMB; Sigma) as the substrate and reading the OD at 630 nm in a Multiskan SkyHigh microplate reader (ThermoScientific, Life Technologies Holdings Pte Ltd., Singapore). The quantity of IL-6 was measured against a standard curve made from 7.8 to 1000 pg/mL IL-6 in two-fold dilutions. At the end of incubation, the viability of the macrophages was measured by adding 30 µL of 5 mg/mL MTT solution in PBS to the remaining 100 µL medium, followed by a 30 min incubation at 37 °C. The formazan formed in the macrophages was dissolved in 200 µL DMSO, and the OD at 570 nm was read in a plate reader. The viability of macrophages incubated in RPMI medium alone in the absence of LPS was set to 100%.

### 2.8. Cytotoxicity Assay Using Vero Epithelial Cells

For measuring cytotoxicity, 4 × 10^4^ Vero epithelial cells were seeded in each well of a 96 flat-bottomed tissue-cultured plate in 200 μL DMEM supplemented with 8% heat-inactivated fetal calf serum, 2 mM L-glutamine, 1 mM sodium pyruvate, and penicillin/streptomycin and incubated overnight at 37 °C. On the following day, the medium was discarded, and 100 μL of serum-free RPMI (control) or 100 μL of the polymer-conditioned media that had been incubated with the polymer scaffolds was added to the cells. Then, 100 μL of DMEM supplemented with 8% heat-inactivated FCS was added, and the plates were incubated for 24 h at 37 °C. The viability of the Vero cells at the end of incubation was measured by adding 30 µL of 5 mg/mL MTT solution in PBS to the cultures for a 30 min incubation at 37 °C. The formazan formed in the Vero cells was dissolved in 200 µL DMSO, and the OD at 570 nm was read in a plate reader. The metabolic activity of the Vero cells was calculated against control samples without the scaffold-conditioned medium, which was set to 100%. Total cell mass was determined by staining the cells with a 0.25% crystal violet (CV) solution (Merck, EMD Millipore Corporation, Billerica, MA, USA) for 20 min, followed by two washes in 200 µL DDW. The CV stain was dissolved in 200 µL 33% acetic acid, and the absorbance measured at 595 nm in a spectrophotometer [34]. The viability of Vero cells incubated in medium alone was set to 100%.

### 2.9. Quantification of CBD in Supernatants by Gas Chromatography–Mass Spectrometry (GC-MS)

The quantification of CBD in the conditioned medium from the placebo and CBD scaffolds was analyzed by GC-MS. The scaffolds were exposed daily to 1 mL of 10 mM Tris-HCl pH 6.8 and kept at −20 °C until analysis. In total, 500 µL of samples from day 1, 3, 7, and 15 in 10 mM Tris-HCl pH 6.8 was lyophilized by the CoolSafe Basic 110-4 freeze dryer (LaboGene, Lillerød, Denmark) and reconstituted in 50 µL hexane with sonication for 5 min (SW 3/H Ultrasonic bath, Sono Swiss, Ramsen, Switzerland). This procedure was performed to concentrate the samples 10-fold. CBD was immediately dissolved in hexane. Following centrifugation at 17,950× *g* at 4 °C for 15 min, the clarified supernatants were transferred to HPLC injection vials (Lab Logistics Group GmbH, Meckenheim, Germany). Samples were analyzed using the 5978 GC/MSD system (Agilent, Santa Clara, CA, USA) and the HP-5MS ultra-inert (UI) capillary column (30 m long, 0.25 mm internal diameter, 0.25 µm film thickness; Agilent Technologies, Santa Clara, CA, USA) under pulsed split mode. The parameters were as follows: injection volume of 1 μL; carrier gas: helium (constant velocity, initial flow rate of 1 mL/min); oven temperature of 60 °C (1 min hold) ramped up to 300 °C at a rate of 20–25 °C/min and maintained at 300 °C for 19 min; interface temperature of 250 °C; tune type: electron ionization (EI); and scan range of *m*/*z* 40–600. CBD was observed at a retention time of 11.4 min and quantified versus a standard calibration curve, with a limit of quantification (LOQ) of 500 ng/mL (1.59 µM) and a limit of detection (LOD) of 250 ng/mL (0.8 µM) [55,56]. Examples of chromatograms of CBD measurements from day 3 and CBD standards for calibration curves are presented in Supplementary File S2. When the quantification program detects CBD, it draws a single red peak on the top of the chromatogram, while in the absence of CBD, it draws multiple noise peaks at the region of CBD’s retention time. The presence of POLYSORB degradation products did not interfere with CBD quantification.

### 2.10. Statistical Analysis

The experiments were performed in triplicate or quadruplicate as indicated in the figure legends. The data are presented as the average ± standard deviation. Statistical significance was calculated according to the Student’s *t*-test and ANOVA with Bonferroni post hoc correction according to sample size. A *p* value less than 0.05 was considered statistically significant when comparing between groups.

## 3. Results

### 3.1. Choice of Solvent for Preparation of PURASORB/CBD Scaffolds for Sustained Release

The aim of this research was to investigate whether the incorporation of CBD into PURASORB 5010 or PURASORB 7510 could provide a sustained-release delivery system that has antibacterial activity against Gram-positive bacteria for prolonged periods of time. In contrast to the use of PLGA polymers in the form of nanoparticles with a high surface area resulting in short half-life [18,19], this research used the polymers in flakes which are expected to have a longer half-life due to reduced surface area. The first step of preparing the PURASORB/CBD scaffolds was to choose a solvent that can dissolve both PURASORB and CBD without interfering with the antibacterial activity of CBD. The preparation should also allow the evaporation of the solvent to achieve a dry polymer with or without CBD. Ethanol dissolves CBD but not PURASORB and can therefore not be used. DMSO and acetone dissolve both CBD and PURASORB. Since both solvents can be evaporated, these were chosen for this study. The minimum inhibitory concentration (MIC)_CBD_ for the three bacterial strains used in this study (*S. mutans* UA159, *S. aureus* ATCC25923 and MDRSA CI-M) was 5 µg/mL when the stock solution of CBD (10 mg/mL) was prepared in either of the four solvents: ethanol, DMSO, PEG400, or acetone (Supplementary Figure S1). While ethanol, DMSO, and acetone solutions provided a light-yellow solution of CBD, the PEG400 solution had a light-pink color. The presence of 1% of each of the solvents in the bacterial broth medium did not affect bacterial growth.

### 3.2. Long-Term Antibacterial Activity of PURASORB/CBD Scaffolds Prepared by Using DMSO as the Solvent

**Figure 2:**
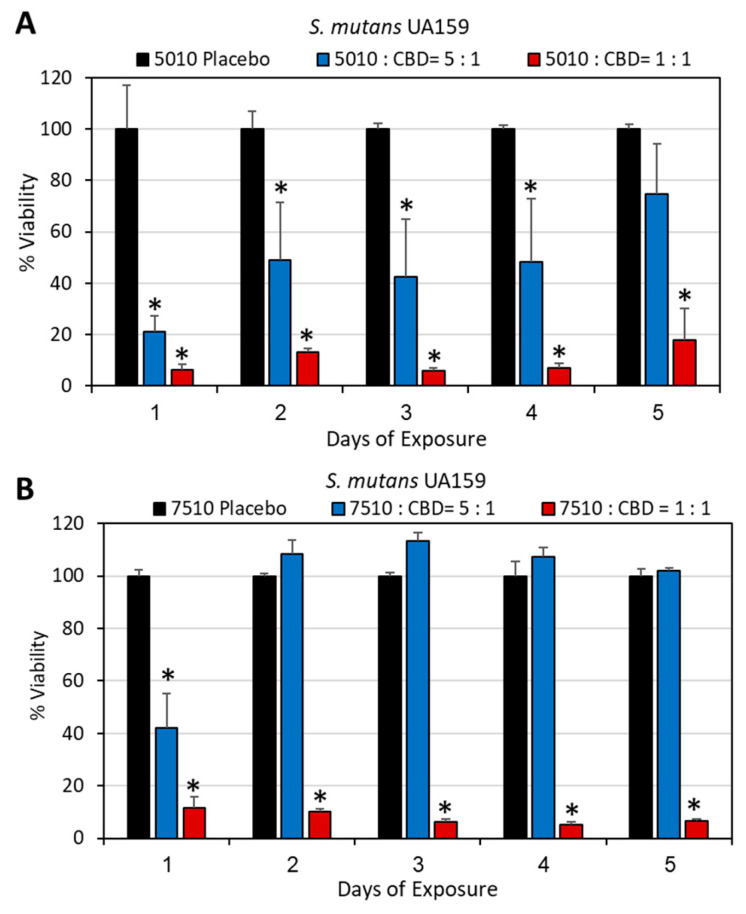
(**A**,**B**) Antibacterial activity of the PURASORB 5010 (**A**) and 7510 (**B**) scaffolds containing two different ratios of polymer to CBD against *S. mutans*. Each scaffold contained 12.5 mg polymer alone or with either 2.5 or 12.5 mg CBD. DMSO was used as the solvent during the preparation of the scaffolds. The scaffolds were exposed daily to fresh *S. mutans* cultures in BHI broth, and the OD at 600 nm of the supernatant was measured after a 24 h incubation. N = 4. * *p* < 0.05 when compared to the placebo.

**Figure 3:**
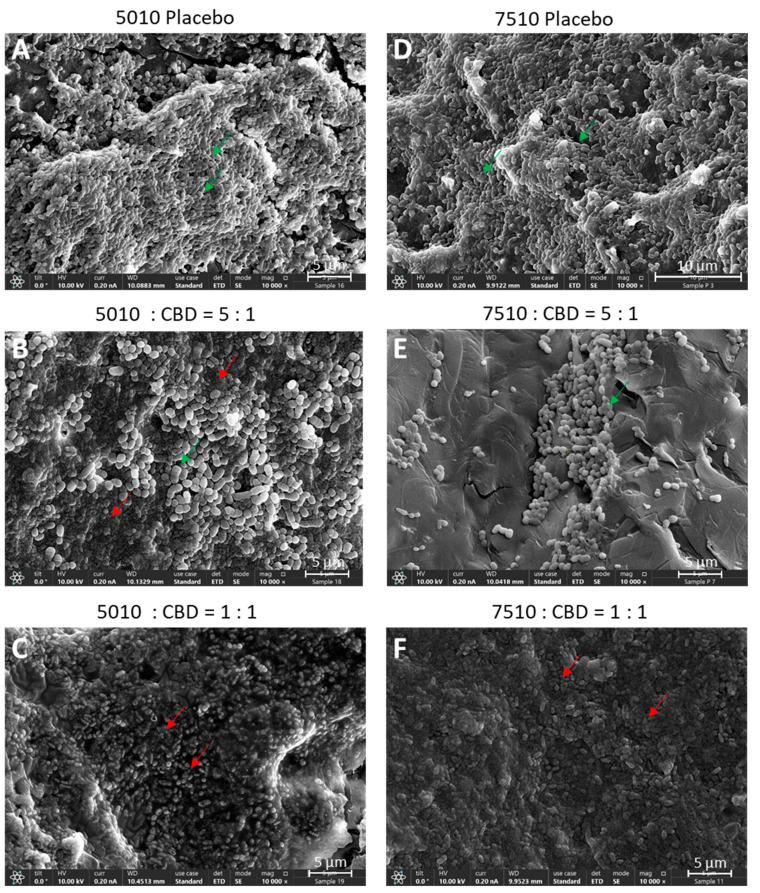
(**A**–**F**) HR-SEM images of the PURASORB 5010 (**A**–**C**) and PURASORB 7510 (**D**–**F**) scaffolds with or without CBD after five times of exposure to fresh *S. mutans* cultures in BHI broth. Each scaffold contained 12.5 mg polymer alone (**A**,**D**) or with either 2.5 mg (ratio 5:1) (**B**,**E**) or 12.5 mg (ratio 1:1) CBD (**C**,**F**). DMSO was used as the solvent during the preparation of the scaffolds. The magnification is ×10,000, and the bars represent 5 μm, except for (**D**) where the bar is 10 μm. The live bacteria appear as smooth luminous 3D structures (green arrows), while the dead bacteria appear as smaller, dysmorphic bacteria that have lost the classical bacterial morphology and appear as darker structures (red arrows). Higher magnification is presented in Supplementary Figure S2.

DMSO was chosen as the solvent for the first series of experiments as CBD is stable in this solvent [57], and its antibacterial activity is preserved ([57]; Supplementary Figure S1). We first determined which polymer-to-CBD ratio is optimal to reach antibacterial activity over time. To this end, we tested the effect of two polymer-to-CBD ratios (5:1 and 1:1) on the growth of *S. mutans,* by daily exposing the polymer scaffolds to a fresh bacterial culture for five consecutive days. The scaffolds with a 1:1 polymer/CBD ratio showed antibacterial activity against *S. mutans* for the entire test period of 5 days (82–95% reduction in bacterial growth; Figure 2A,B), while the scaffolds of 5:1 polymer/CBD ratio were only significantly effective during the first exposure to bacteria (60–80% inhibition) (Figure 2A,B) with a decline on the following days (Figure 2A,B). PURASORB 5010/CBD at a 5:1 ratio showed a 51–58% reduction in bacterial growth on days 2–4 (Figure 2A), while the PURASORB 7510/CBD at a 5:1 ratio lost antibacterial activity on day 2 (Figure 2B). The HR-SEM images of scaffolds exposed five times to *S. mutans* showed multilayered bacterial biofilm with smooth luminous 3D structures on placebo scaffolds indicative of live bacteria (Figure 3A,D; Supplementary Figure S2A,D), while a single layer of apparently dead, shriveled bacteria was seen on scaffolds with a polymer/CBD ratio of 1:1 (Figure 3C,F; Supplementary Figure S2C,F). PURASORB 5010 scaffolds with the lower amount of CBD showed both dead and live bacteria on the surface (Figure 3B; Supplementary Figure S2B), while PURASORB 7510 scaffolds with the lower amount of CBD showed scattered clusters of live bacteria (Figure 3E; Supplementary Figure S2E). The apparent live bacteria on the PURASORB 5010/CBD and PURASORB 7510/CBD scaffolds showed a more rounded and swollen structure (Figure 3B,E; Supplementary Figure S2B,C) compared to the classical ovoid structure of *S. mutans* on the placebo scaffolds (Figure 3A,D; Supplementary Figure S2A,D). The surface coverage of the bacteria was lower on the PURASORB 5010/7510 scaffolds containing CBD compared to the placebo.

**Figure 4:**
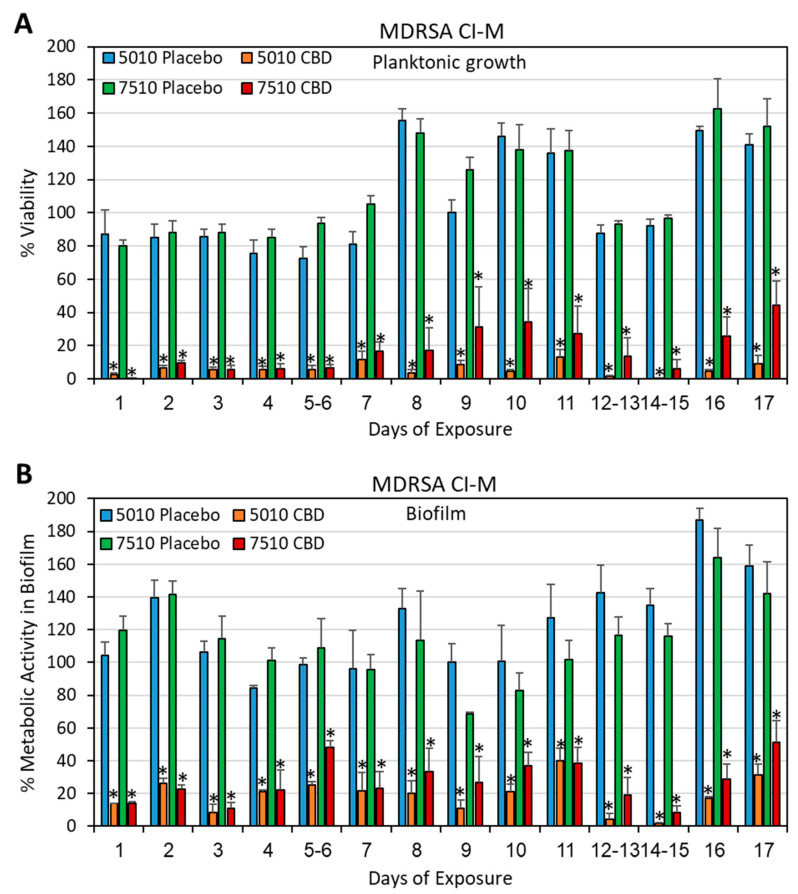
(**A**,**B**) Long-term antibacterial (**A**) and antibiofilm (**B**) activity of the polymer/CBD scaffolds against MDRSA CI-M. The PURASORB 5010 and 7510 scaffolds with or without CBD at a polymer-to-CBD ratio of 2:1 (25 mg polymer and 12.5 mg CBD per scaffold) were exposed daily to a fresh culture of MDRSA CI-M in TSB for a 24 h incubation. The viability of bacteria in the supernatant was determined by measuring the OD at 600 nm (**A**), while the metabolic activity of biofilms formed on the well surface was determined by the MTT assay (**B**). The % viability and metabolic activity was calculated against control samples without scaffolds, which was set to 100%. DMSO was used as the solvent during the preparation of the scaffolds. N = 4. * *p* < 0.05 when compared to the placebo samples.

**Figure 5:**
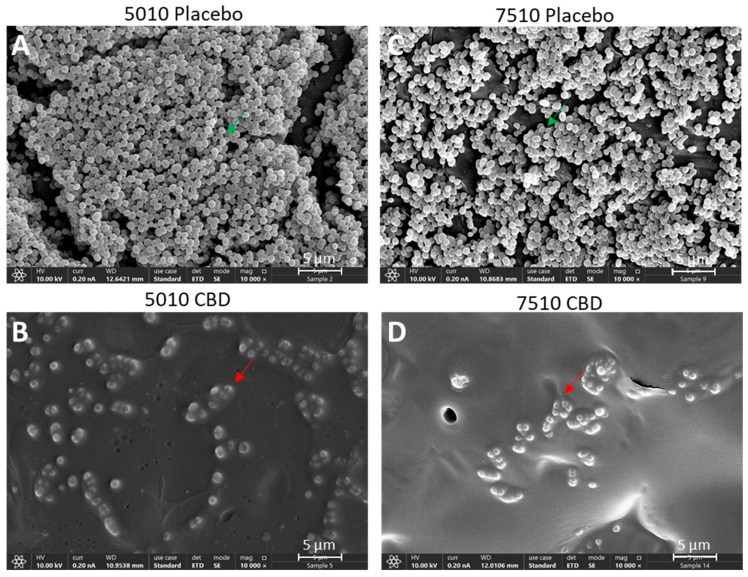
HR-SEM images of the PURASORB 5010 (**A**,**B**) and PURASORB 7510 (**C**,**D**) scaffolds with or without CBD after seven times of exposure to fresh MDRSA CI-M cultures in TSB. Each scaffold contained 25 mg polymer alone (**A**,**C**) or with 12.5 mg CBD (**B**,**D**). DMSO was used as the solvent during the preparation of the scaffolds. The magnification is ×10,000, and the bars represent 5 μm. The live bacteria appear as luminous, round 3D structures with smooth membranes (green arrows), while the dead bacteria appear shriveled with a lack of a defined surface and a lack of cell boundaries (red arrows). Higher magnification is presented in Supplementary Figure S3.

DMSO was also used as a solvent to prepare PURASORB scaffolds with a polymer-to-CBD ratio of 2:1, where each scaffold contained 25 mg of polymer and 12.5 mg of CBD (Figure 1A). The CBD/polymer scaffolds showed antibacterial and antibiofilm activities against the multidrug-resistant *S. aureus* strain MDRSA CI-M for the entire test period of 17 days (Figure 4A,B). The HR-SEM images of polymer scaffolds exposed seven times to MDRSA CI-M showed multilayered biofilms of luminous, live bacteria on placebo scaffolds with classical round 3D structures (Figure 5A,C; Supplementary Figure S3A,C). In contrast, the CBD-containing polymers showed scattered dead bacteria attached to the scaffolds with dysmorphic, shriveled structures (Figure 5B,D; Supplementary Figure S3B,D). Altogether, these data show that the PURASORB/CBD scaffolds prepared using DMSO as the solvent had a long-term antibacterial and antibiofilm effect against *S. mutans* and MDRSA CI-M.

### 3.3. Long-Term Antibacterial Activity of the PURASORB/CBD Scaffolds Prepared by Using Acetone as the Solvent

**Figure 6:**
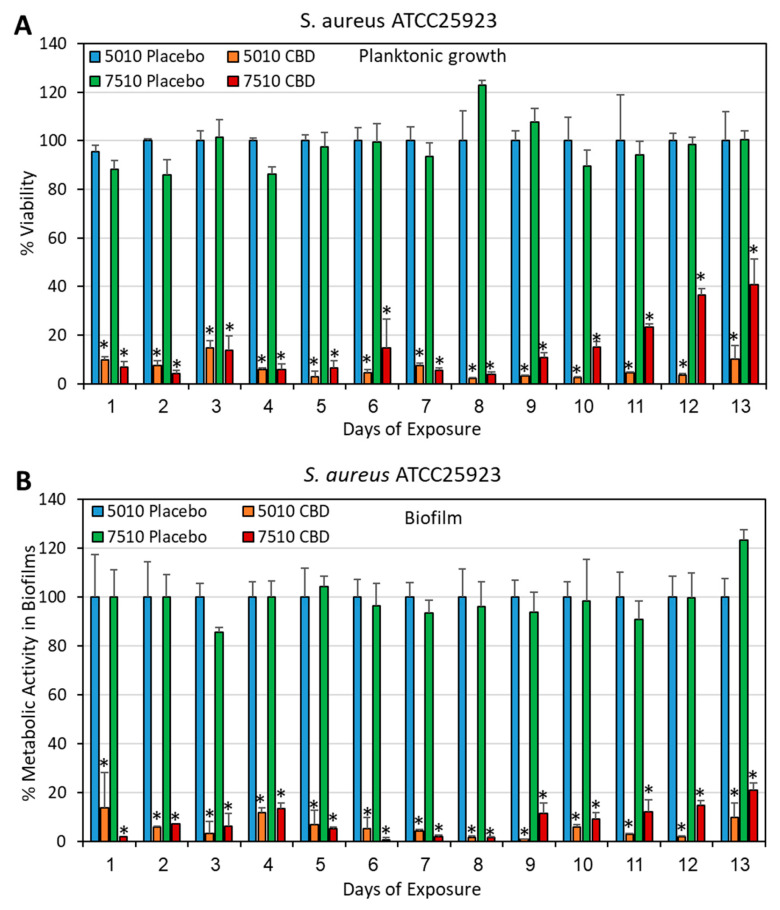
(**A**,**B**) Long-term antibacterial (**A**) and antibiofilm (**B**) activity of the polymer/CBD scaffolds against *S. aureus* ATCC25923. The PURASORB 5010 and 7510 scaffolds with or without CBD at a polymer-to-CBD ratio of 2:1 (25 mg polymer and 12.5 mg CBD per scaffold) were exposed daily to a fresh culture of *S. aureus* ATCC25923 in TSB for a 24 h incubation. The viability was determined by measuring the OD of the supernatant at 600 nm (**A**), while the metabolic activity of biofilms formed at the well surface was determined by the MTT assay (**B**). Acetone was used as the solvent during the preparation of the scaffolds. N = 4. * *p* < 0.05 compared to the placebo.

**Figure 7:**
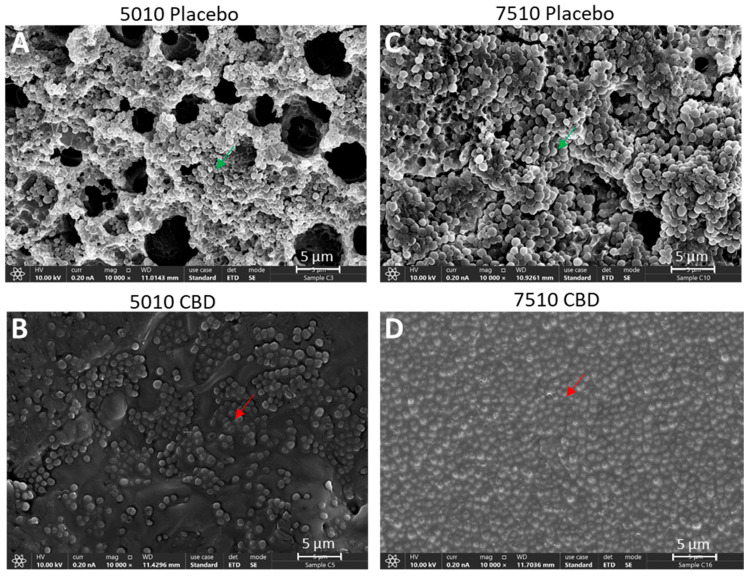
HR-SEM images of the PURASORB 5010 (**A**,**B**) and PURASORB 7510 (**C**,**D**) scaffolds with or without CBD after 13 times of exposure to fresh *S. aureus* ATCC25923 cultures in TSB. Each scaffold contained 25 mg polymer alone (**A**,**C**) or with 12.5 mg CBD (**B**,**D**). Acetone was used as the solvent during the preparation of the scaffolds. The magnification is ×10,000, and the bars represent 5 μm. The live bacteria appear as luminous, round 3D structures with smooth membranes (green arrows), while the dead bacteria appear darker with flat and shriveled structures (red arrows). Higher magnification is presented in Supplementary Figure S4.

**Figure 8:**
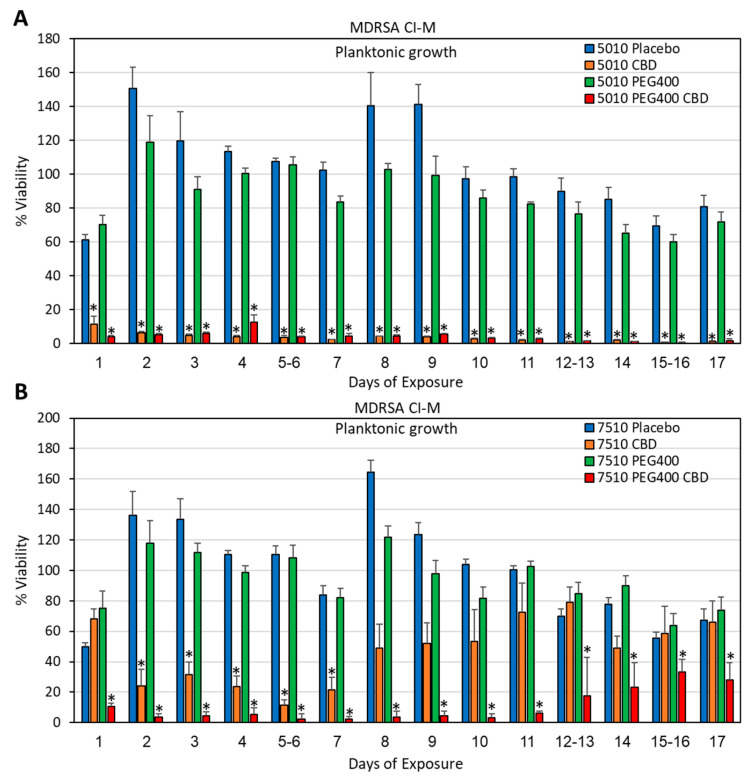
(**A**,**B**) Long-term antibacterial activity of the polymer/CBD scaffolds against MDRSA CI-M. The PURASORB 5010 (**A**) and 7510 (**B**) scaffolds with or without CBD at a polymer-to-CBD ratio of 2:1 (25 mg polymer and 12.5 mg CBD per scaffold) and with or without 7% PEG400 were exposed daily to a fresh culture of *S. aureus* MDRSA CI-M in TSB for a 24 h incubation. The viability was determined by measuring the OD of the supernatant at 600 nm. Acetone was used as the solvent during the preparation of the scaffolds. Samples without any PURASORB scaffolds were used as controls and set to 100%. N = 4–5. * *p* < 0.05 compared to the placebo and control samples.

**Figure 9:**
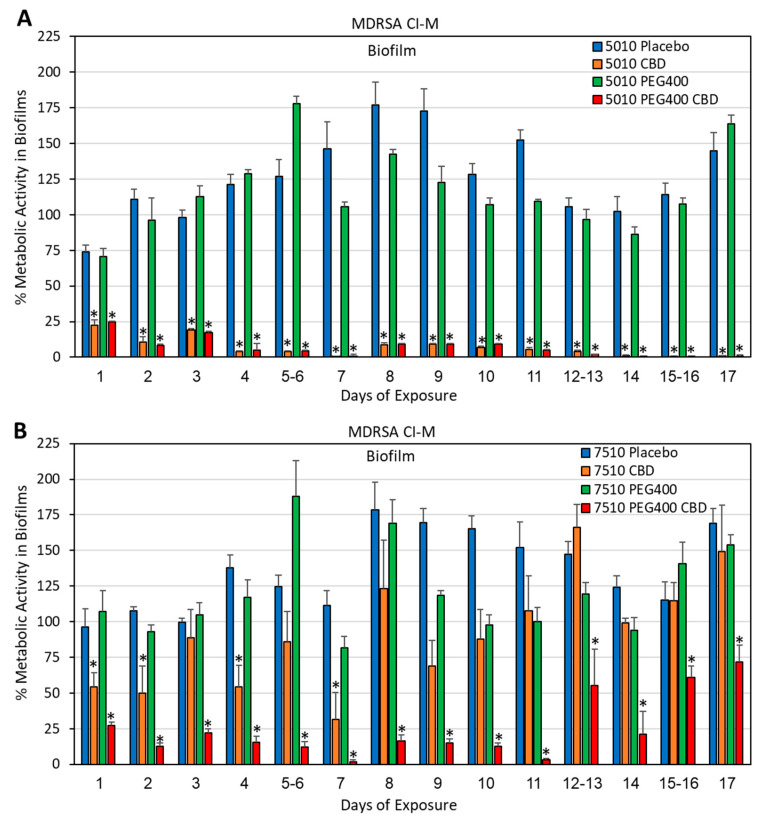
(**A**,**B**) Long-term antibiofilm activity of the polymer/CBD scaffolds against MDRSA CI-M. **The** PURASORB 5010 (**A**) and 7510 (**B**) scaffolds with or without CBD at a polymer-to-CBD ratio of 2:1 (25 mg polymer and 12.5 mg CBD per scaffold) and with or without 7% PEG400 were exposed daily to a fresh culture of MDRSA CI-M in TSB for a 24 h incubation. The metabolic activity of the biofilms that formed at the well surface was determined by the MTT assay. Acetone was used as the solvent during the preparation of the polymer scaffolds. Samples without any scaffolds were used as the control and set to 100%. N = 5, except for PURASORB 7510/CBD with N = 4. * *p* < 0.05 compared to the placebo and control samples.

**Figure 10:**
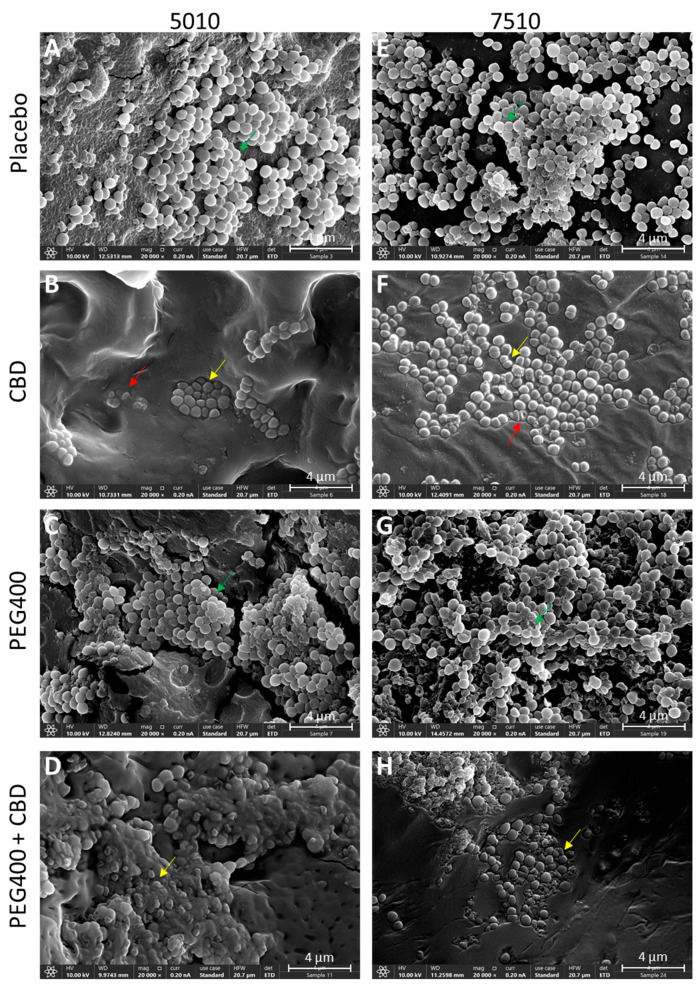
HR-SEM images of the PURASORB 5010 (**A**–**D**) and PURASORB 7510 (**E**–**H**) scaffolds with or without CBD at a ratio of 2:1 and with or without 7% PEG400 after 17 days of exposure to fresh MDRSA CI-M cultures in TSB. Each scaffold contained 25 mg polymer alone (**A**,**E**) or with 12.5 mg CBD (**B**,**D**,**F**,**H**) and/or 7% PEG400 (**C**,**D**,**G**,**H**). Acetone was used as the solvent during the preparation of the scaffolds. The magnification is ×20,000, and the bars represent 4 μm. The appearance of the bacteria on the CBD-containing polymer scaffolds (**B**,**F**) differs from those on the placebo polymer scaffolds (**A**,**E**) (red and yellow arrows versus green arrows). The incorporation of PEG400 still caused the adherence of live bacteria (**C**,**G**) (green arrows), while the bacteria adhered to the polymer scaffolds containing both PEG400 and CBD showed distorted structures (**D**,**H**) (yellow arrows), suggesting that PEG400 enhances the antibacterial effect of CBD on the polymer surface.

Although the polymer/CBD scaffolds formed by using DMSO as a solvent showed long-term antibacterial activity (Section 3.2), the long time (around 2 weeks) required to evaporate DMSO under vacuum is a disadvantage during preparation. We therefore decided to test the antibacterial activity of the polymer/CBD scaffolds prepared by using acetone as the solvent, which quickly evaporates at room temperature within a couple of hours. The acetone-prepared PURASORB 5010/7510 scaffolds with CBD at a ratio of 2:1 were shown to have antibacterial and antibiofilm activity against *S. aureus* 25923 (Figure 6 and Figure 7) and MDRSA CI-M (Figure 8, Figure 9 and Figure 10). The HR-SEM images of the PURASORB/CBD scaffolds that had been exposed for 13 days to *S. aureus* 25923 showed a layer of apparently dead bacteria (Figure 7B,D; Supplementary Figure S4B,D), while the placebo scaffolds had multilayered biofilms of live bacteria (Figure 7A,C; Supplementary Figure S4A,C). The dead bacteria have lost the classical 3D smooth and luminous structure observed for live bacteria and appeared darker and shriveled (Figure 7B,D; Supplementary Figure S4B,D). Similar observations were seen for the PURASORB/CBD scaffolds that had been exposed for 17 days to MDRSA CI-M (Figure 10B,F versus Figure 10A,E).

**Figure 11:**
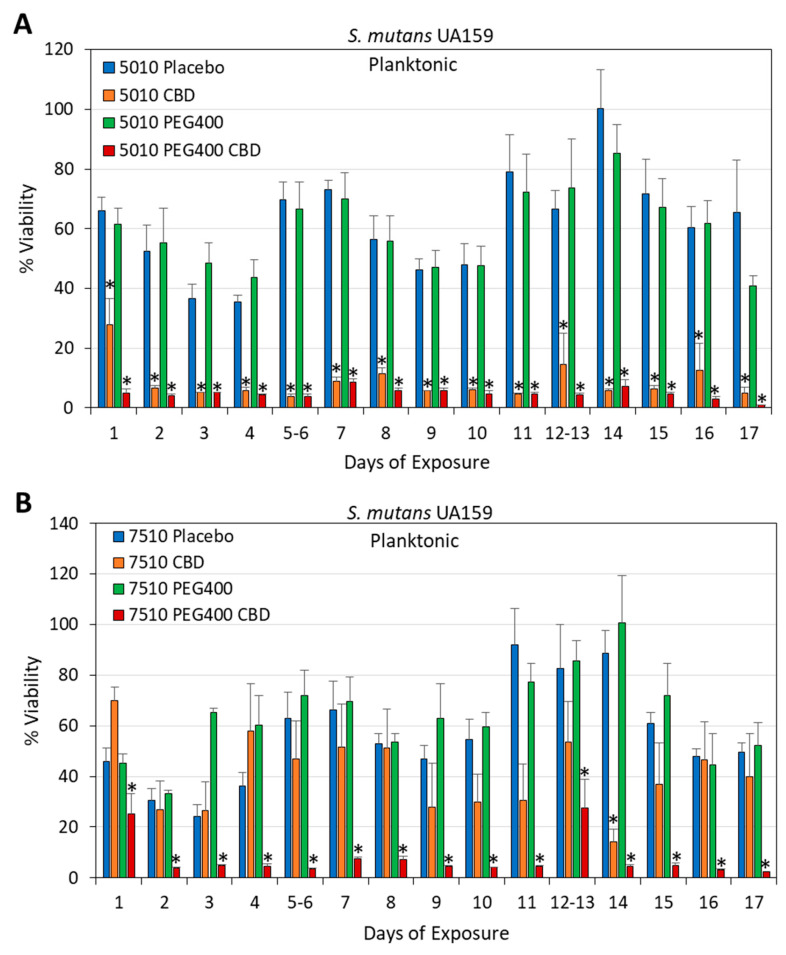
(**A**,**B**) Long-term antibacterial activity of the polymer/CBD scaffolds against *S. mutans* UA159. The PURASORB 5010 (**A**) and 7510 (**B**) scaffolds with or without CBD at a polymer-to-CBD ratio of 2:1 (25 mg polymer and 12.5 mg CBD per scaffold) and with or without 7% PEG400 were exposed daily to a fresh culture of *S. mutans* in BHI broth for a 24 h incubation. The % viability was determined by measuring the OD of the supernatant at 600 nm and setting control samples without scaffolds to 100%. Acetone was used as the solvent during the preparation of the scaffolds. N = 4. * *p* < 0.05 compared to the placebo and control samples. PURASORB 7510/PEG400/CBD had significantly better antibacterial activity against *S. mutans* than the PURASORB 7510/CBD scaffolds (*p* < 0.05).

**Figure 12:**
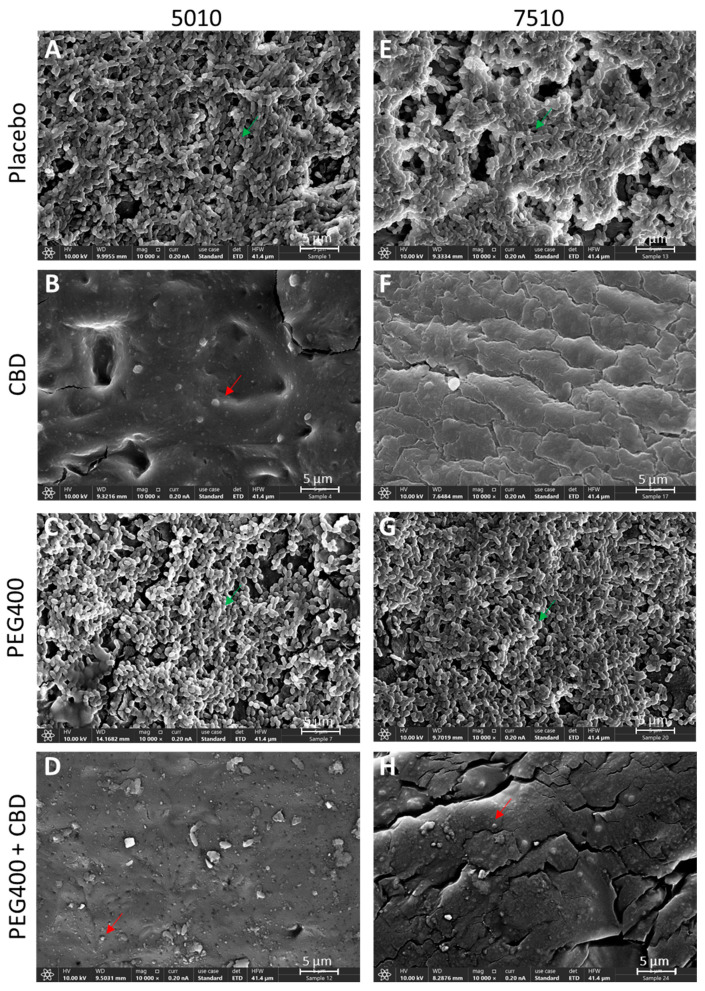
HR-SEM images of the PURASORB 5010 (**A**–**D**) and PURASORB 7510 (**E**–**H**) scaffolds with or without CBD at a ratio of 2:1 (**B**,**D**,**F**,**H**) and with or without 7% (*w*/*w*) PEG400 (**C**,**D**,**G**,**H**) after 17 days of exposure to fresh *S. mutans* UA159 cultures in BHI broth. Each scaffold contained 25 mg polymer alone (**A**,**E**) or with 12.5 mg CBD (**B**,**D**,**F**,**H**) and/or 7% (*w*/*w*) PEG400 (**C**,**D**,**G**,**H**). Acetone was used as the solvent during the preparation of the scaffolds. The magnification is ×10,000, and the bars represent 5 μm. Green arrows point to live bacteria with luminous 3D structures, while red arrows point to dead bacteria with a dysmorphic structure.

In particular, when using acetone as the solvent for the preparation, PURASORB 5010/CBD showed an overall better long-term antibacterial effect than PURASORB 7510/CBD (Figure 6, Figure 8 and Figure 9; *p* < 0.05). The superiority of PUROSORB 5010/CBD over PURASORB 7510/CBD was even more pronounced when the scaffolds were exposed to *S. mutans* (Figure 11). PURASORB 5010/CBD showed antibacterial activity against *S. mutans* for the entire test period of 17 days (Figure 11A), while there was no significant difference in bacterial growth in the presence of PURASORB 7510 with or without CBD (Figure 11B). The HR-SEM images of the polymer scaffolds exposed to *S. mutans* for 17 days showed multilayered biofilms on the PURASORB 5010 and PURASORB 7510 placebo scaffolds (Figure 12A,E), with only few scattered dead bacteria on the PURASORB 5010/CBD and PURASORB 7510/CBD scaffolds (Figure 12B,F).

In an attempt to increase the antibacterial durability of the PURASORB samples, PEG400 was added to the polymers. PEG400 is a hydrophilic polymer known to increase the porosity of polymers and assist in drug release [58]. The incorporation of PEG400 into the polymer/CBD scaffolds at a final concentration of 7% (*w*/*w*) significantly increased the duration of the antibacterial activity of the PURASORB 7510/CBD scaffolds but not that of the PURASORB 5010/CBD scaffolds (Figure 8 and Figure 11). The HR-SEM images of the polymer/PEG400/CBD scaffolds that had been exposed to MDRSA CI-M for 17 days showed only few live bacteria and several bacteria with distorted structure, which is in contrast to the polymer/PEG400 scaffolds where multilayered biofilms of live bacteria with clear luminous 3D structures were seen (Figure 10D,H, versus Figure 10C,G). Similarly, the HR-SEM images of the polymer/PEG400/CBD scaffolds that had been exposed to *S. mutans* for 17 days showed multilayered biofilm layers on the polymer/PEG400 scaffolds (Figure 12C,G), while only scattered dead bacteria can be seen on the surface of the polymer/PEG400/CBD scaffolds (Figure 12D,H). Altogether, these data show that the PURASORB/CBD scaffolds prepared using acetone as the solvent had long-term antibacterial and antibiofilm effect against *S. mutans* as well as methicillin-sensitive and methicillin-resistant *S. aureus*. The addition of PEG400 increased the long-term antibacterial and antibiofilm effect of the PURASORB 7510/CBD scaffolds.

### 3.4. PURASORB/CBD Scaffolds Reduced IL-6 Secretion from LPS-Stimulated Macrophages

**Figure 13:**
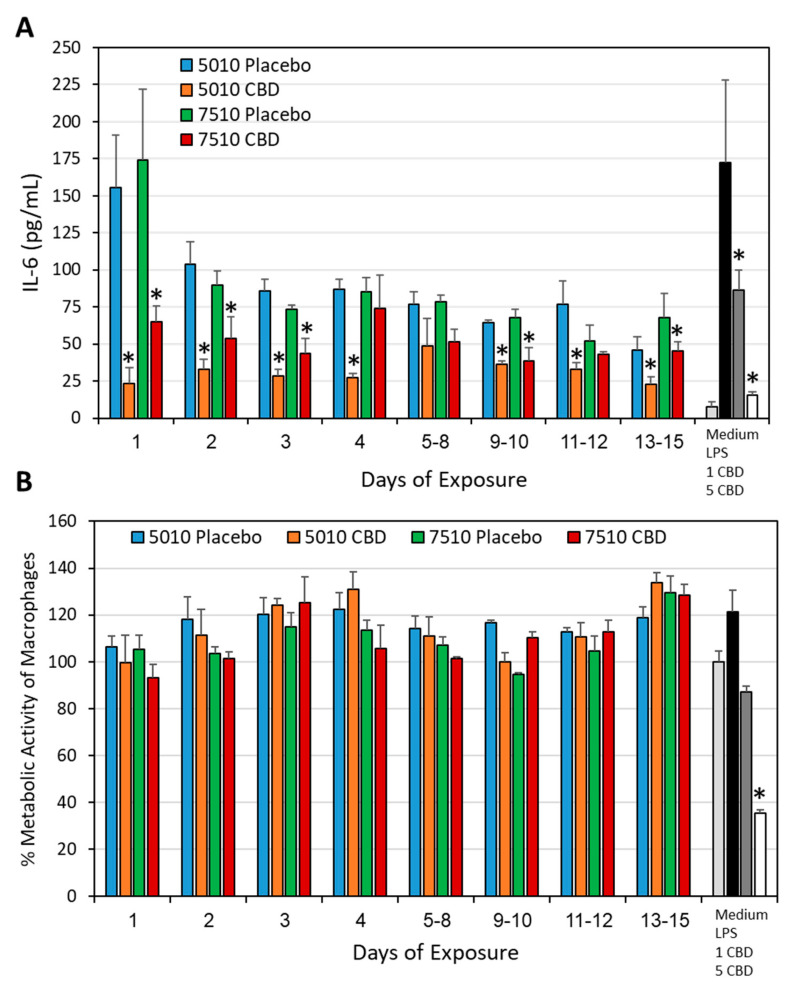
Long-term inhibition of the IL-6 secretion from LPS-stimulated macrophages. (**A**) The effect of the PURASORB 5010 and PURASORB 7510 scaffolds with or without CBD on the IL-6 secretion from LPS-stimulated RAW 264.7 macrophages. The gray-scaled columns at the very right present the IL-6 secretion from RAW 264.7 macrophages in the medium alone, in the presence of 10 ng/mL LPS alone or together with either 1 μg/mL or 5 μg/mL CBD after a 6 h incubation. The IL-6 concentration was determined by ELISA. (**B**) The metabolic activity of RAW 264.7 macrophages after 6 h incubation with the indicated treatments as indicated in (**A**). The polymer/CBD scaffolds were prepared by using DMSO as the solvent. N = 3. * *p* < 0.05 compared to the placebo and LPS-treated macrophages.

**Figure 14:**
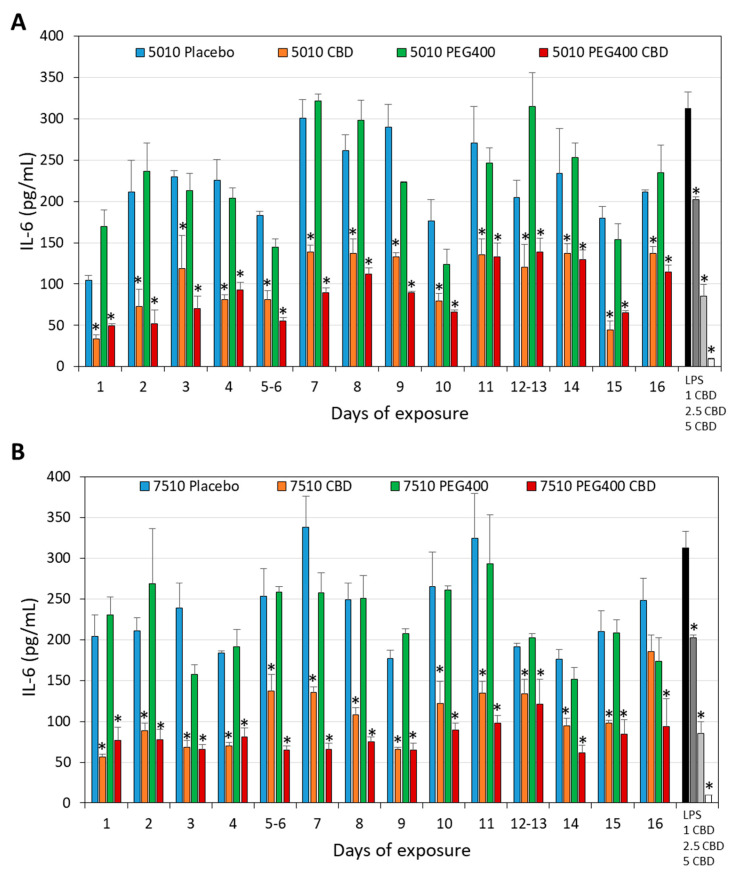
Long-term inhibition of the IL-6 secretion from LPS-stimulated macrophages. (**A**,**B**) The effect of the PURASORB 5010 (**A**) and PURASORB 7510 (**B**) scaffolds with or without CBD and/or PEG400 on the IL-6 secretion from LPS-stimulated RAW 264.7 macrophages. The gray-scaled columns at the very right presents the IL-6 secretion from RAW 264.7 macrophages in the presence of 10 ng/mL LPS alone or together with either 1 μg/mL, 2.5 μg/mL or 5 μg/mL CBD after a 6 h incubation. The IL-6 concentration was determined by ELISA. The polymer/CBD scaffolds were prepared by using acetone as the solvent. N = 3. * *p* < 0.05 compared to the placebo and LPS-treated macrophages.

Another parameter that was important to study was whether the daily release of CBD from the PURASORB scaffolds was sufficient to exert anti-inflammatory activity. It is known that CBD has an anti-inflammatory effect and reduces cytokine production by macrophages, which are innate immune cells involved in inflammation [59,60,61,62]. The PURASORB/CBD scaffolds were exposed daily to the RPMI-1640 medium, and the conditioned RPMI-1640 medium was tested for its effect on the IL-6 secretion from LPS-stimulated RAW 264.7 macrophages. The PURASORB/CBD scaffolds prepared by using DMSO as the solvent showed stronger inhibition of the IL-6 secretion (81.6 ± 4.8% and 70.0 ± 6.9% inhibition at the average with PURASORB 5010/CBD and PURASORB 7510/CBD, respectively) than the respective placebo scaffolds, although the placebo scaffolds also contributed to the inhibition (49.6 ± 10.5% inhibition; Figure 13A). The metabolic activity of the macrophages at the end of the incubation was not significantly affected by the scaffold-conditioned RPMI-1640 media (Figure 13B), indicating that the macrophages were alive. Notably, CBD at a concentration of 5 μg/mL significantly reduced the metabolic activity of the macrophages (Figure 13B). This suggests that the daily amount of active CBD released from the polymers is less than 5 μg. Similarly, the PURASORB/CBD scaffolds prepared from acetone showed significant reduction in the IL-6 secretion from LPS-stimulated RAW 264.7 macrophages (71.2 ± 11.9% inhibition) in comparison to the PURASORB-placebo (25.3 ± 16.3% inhibition) (Figure 14; *p* < 0.05). A dose-dependent reduction in the IL-6 secretion was observed for CBD (35.2 ± 1.0, 72.7 ± 4.5 and 86.9 ± 0.1% inhibition with 1, 2.5, and 5 µg/mL CBD, respectively; Figure 14), suggesting that the average effective CBD release from the polymer scaffolds was around 2.5 µg. The metabolic activity of the macrophages was reduced by around 22.9 ± 14.6% by the conditioned medium from the polymer scaffolds with no significant difference between the placebo and the CBD-containing scaffolds (Supplementary Figure S5). These data show that the amount of CBD released is sufficient for the long-term inhibition of the IL-6 secretion from LPS-activated macrophages without a significant cytotoxic effect.

### 3.5. The Daily Release of CBD from PURASORB/CBD Scaffolds Was Not Cytotoxic to Vero Epithelial Cells

**Figure 15:**
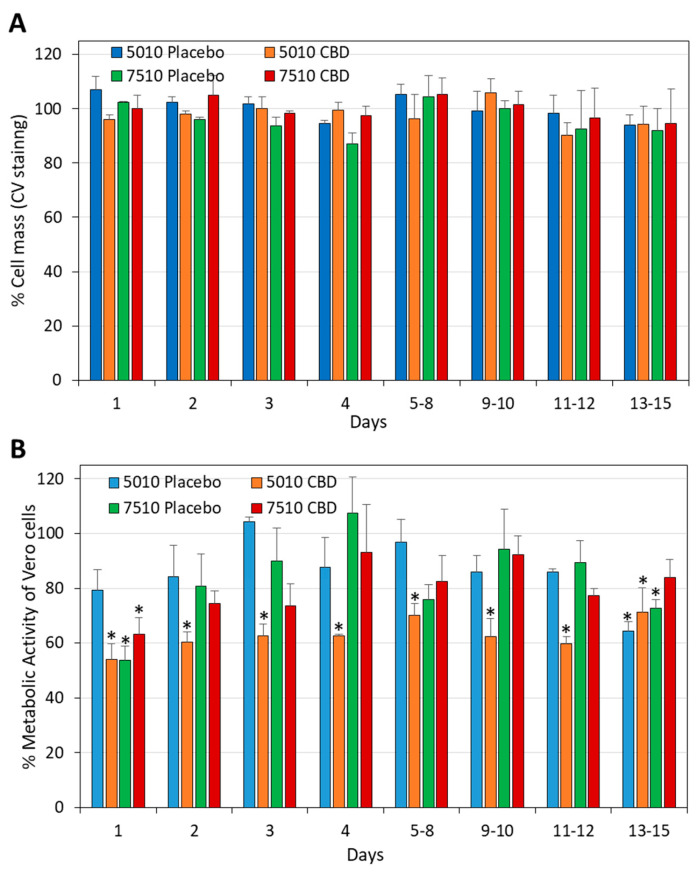
Cytotoxicity assay using Vero epithelial cells. (**A**,**B**) Vero epithelial cells in monolayers were incubated with RPMI supernatants that had been exposed daily to the PURASORB 5010 or 7510 scaffolds with or without CBD at a polymer/drug ratio of 2:1 (25 mg polymer and 12.5 mg CBD). After a 24 h incubation, the total cell mass was determined by crystal violet (CV) staining (**A**), and the metabolic activity of the cells was determined by the MTT assay (**B**). The Vero cells receiving only RPMI were set to 100%. The scaffolds were prepared using DMSO as the solvent. * *p* < 0.05 compared to the placebo and control samples.

Next, we studied the effect of the PURASORB/CBD-conditioned media on the viability of Vero epithelial cells, which are considered the standard cell line for studying cytotoxicity. After a 24 h incubation, the cells were either stained with crystal violet (CV), or the metabolic activity was determined by the MTT assay. The conditioned media did not reduce the cell mass (Figure 15A), but there was a 37.1 ± 5.5% reduction on average in the metabolic activity with PURASORB 5010/CBD, and a 19.9 ± 10.0% reduction in the metabolic activity with PURASORB 7510/CBD (Figure 15B). The placebo samples caused a 14.0 ± 11.8% reduction on average (Figure 15B). CBD is known for its antimetabolic effect on mammalian cells [63], which can explain the relatively reduced metabolic activity with PURASORB 5010/CBD. Also, the PURASORB/CBD-conditioned media from the polymer scaffolds with or without CBD and/or PEG400 prepared by the acetone procedure showed, in general, no significant cytotoxicity toward Vero epithelial cells with occasional reductions in the metabolic activity (Supplementary Figures S6 and S7). Light microscopy of the treated cells showed no significant cytotoxicity of the scaffold-conditioned media on Vero epithelial cells. Overall, these data indicate that the daily amount of CBD released from the scaffolds is below the cytotoxic level for Vero epithelial cells.

### 3.6. The Determination of CBD Concentration in the Scaffold-Conditioned Media by GC-MS

**Figure 16:**
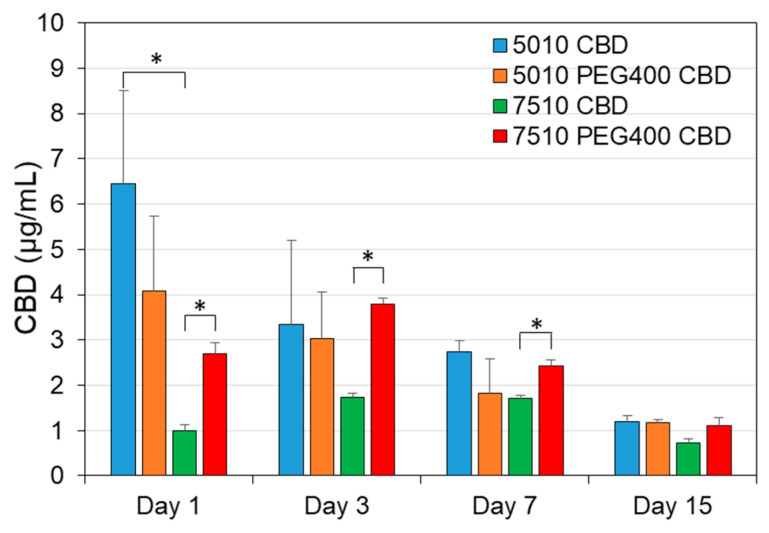
Quantification of CBD levels in the polymer-conditioned media by GC-MS. The PURASORB 5010/7510 scaffolds with or without CBD and/or PEG400 were incubated daily in 1 mL of 10 mM Tris-HCl pH 6.8, and the quantity of CBD in the supernatant was determined by GC-MS after lyophilizing the samples. The highest level was observed for a PURASORB 5010/CBD scaffold on day 1 (9.43 µg/mL), while the lowest level was observed for a PURASORB 7510/CBD scaffold on day 15 (0.575 µg/mL). The placebo scaffolds with or without PEG400 served as controls with no CBD detection. N = 3. * *p* ≤ 0.05.

Next, we determined the CBD concentration in the scaffold-conditioned media on selected days (1, 3, 7 and 15) by GC-MS. The amount of CBD released from PURSORB 5010/CBD (6.46 ± 2.04 µg/mL) was significantly higher than from PURASORB 7510/CBD (0.99 ± 0.14 µg/mL) on day 1 (Figure 16; *p* = 0.05). The incorporation of PEG400 significantly increased the release of CBD from PURASORB 7510 on days 1, 3, and 7 (Figure 16; *p* < 0.05). There was a general trend with the reduced release of CBD over time (Figure 16), but as shown above (Figure 1, Figure 2, Figure 3, Figure 4, Figure 5, Figure 6, Figure 7, Figure 8, Figure 9, Figure 10, Figure 11, Figure 12, Figure 13 and Figure 14), the amount of CBD released was still sufficient to exert antibacterial and anti-inflammatory effects. The quantification of CBD in the conditioned media proved the sustained release of CBD at low quantities that are sufficient for the desired biological effects, while being below the cytotoxic level (25 µg/mL) to mammalian cells ([34]; Figure 15, Supplementary Figures S6 and S7).

## 4. Discussion

The main objective of this research was to develop a sustained-release device for CBD that could provide prolonged antibacterial activity against CBD-susceptible bacteria such as *S. mutans* involved in caries formation and *S. aureus* involved in infectious diseases. The advantage of a sustained-release system is the direct local delivery of the drug to the intended treatment site over a prolonged period of time, which would require lower doses of the drug and higher patient compliance [1,2]. Such a system is expected to have fewer adverse effects compared to those of the systemic administration of the drug. Moreover, it overcomes the problematic aspects of drugs with low absorption, gastrointestinal cytotoxicity, and the metabolic inactivation of the drug in the liver during first passage [1,2]. We chose CBD as the active drug because it is effective against both antibiotic-sensitive and antibiotic-resistant Gram-positive bacteria ([36,37] and Supplementary Figure S1), and so far, the propensity to develop resistance to CBD has been found to be low (<3.78 × 10^−10^) when MRSA was exposed to sub-lethal concentrations of CBD for 20 days [36]. Additionally, CBD has anti-inflammatory and anti-nociceptive effects that may dampen the inflammation and pain associated with infections [62,64,65,66].

We postulated that the incorporation of CBD into a biodegradable polymer of the PLGA family would lead to a gradual release of CBD when the polymer disintegrates, which then would prevent the bacterial growth and biofilm formation of CBD-susceptible bacterial strains. PLGA has several advantages as a drug carrier. It is biodegradable, biocompatible, and has the loading capacity of both hydrophobic and hydrophilic molecules with sustained release of the incorporated compound [17]. Another advantage is that it can be processed into any shape and size. It is an FDA (Food and Drug Administration, Silver Spring, MD, USA)- and EMA (European Medicines Agency)-approved copolymer. PLGA has, in particular, been used in the form of nanoparticles and microspheres in various settings to increase the surface area for improved drug delivery [17]. The degradation rate of PLGA in nanoparticles or microparticles has been shown to be relatively fast, with a half-life of 48 h, and complete degradation after 7 days when a PLGA with an L-lactide-to-glycolide ratio of 65:35 was used [67]. In the circulation, PLGA nanoparticles had a short half-life of a few hours [68], which may be in part due to uptake into cells [69]. Since we were interested in developing a device that would act over a longer period of time, the CBD-incorporated PUROSORB scaffolds were prepared as structures with a relatively smaller surface-to-volume area compared to that of the nanoparticles. The antibacterial activities of CBD using two different PUROSORB scaffolds (5010 and 7510) with different ratios of L-lactide to glycolide (50:50 for PURASORB 5010 and 75:25 for PURASORB 7510) were compared over time. The polyglycolic acid (PGA) component is more hydrophilic, while the polylactic acid (PLA) component is stiffer and more hydrophobic [17]. The higher the ratio of PLA to PGA, the longer the degradation time [17]. The degradation rate of our PURASORB 5010/7510 scaffolds was relatively slow. After 17 days, the net weight of the PURASORB 5010 scaffolds had decreased by 22.4 ± 11.2% and that of the PURASORB 7510 scaffolds by 14.6 ± 8.0% (N = 12). The slower degradation rate of PURASORB 7510 compared to that of PURASORB 5010 may explain why PURASORB 5010/CBD in general has better antibacterial activity over longer periods of time. This is consistent with the CBD quantification data showing higher CBD release from PURASORB 5010. The addition of CBD to the polymers had no significant effect on the degradation rate. Moreover, the addition of PEG400 to the polymer/CBD scaffolds did not increase the degradation rate of the polymers, but it increased the antibacterial activity of the CBD-PURASORB 7510 scaffolds. The CBD quantification data showed that PEG400 facilitated the release of CBD from PURASORB 7510. Other studies have also shown the increased bioavailability of other hydrophobic drugs such as curcumin and paclitaxel when PEG400 was incorporated into PLGA [70,71].

The daily release of CBD from PURASORB 5010/7510 was relatively low compared to the degradation rate of the polymers. Polymer scaffolds with an initial average CBD content of 12.5 mg released less than 2% of the incorporated CBD after 17 days, suggesting that CBD is mainly retained within the polymer. According to the 22.4 ± 11.2% decrease in the PURASORB 5010 scaffold weight observed during the 17 days in fluid, we would have theoretically expected a daily release of 165 ± 82 μg CBD. However, the daily release was found to be between 1.12 and 9.43 µg/mL. The reason for the relatively low CBD release is unclear. It could be due to an interaction between CBD and PLGA, or its low solubility in water (12.6 µg/mL) and high lipophilicity (logP of 6.3) [72] limit the amount of CBD that can be dissolved in the medium, resulting in the slow release of CBD from the scaffolds. Regardless of the mechanisms, the daily amount of CBD released is sufficient to exert antibacterial activity against *S. mutans* and *S. aureus* and to dampen the inflammatory activity of macrophages over time without having a cytotoxic effect on mammalian cells. It is actually important that only a small amount, but still active CBD is released daily to avoid cytotoxic effects on healthy tissue. CBD is cytotoxic to Vero epithelial cells at 25 µg/mL [34], which is far above the daily effective release of CBD from our polymer/CBD devices.

Not only did the PURASORB/CBD scaffolds prevent bacterial growth in their vicinity, but the presence of CBD in the scaffolds also prevented bacterial growth and biofilm formation on the scaffolds. This has implications for their application on medical devices that frequently suffer from infections by bacterial biofilms [73]. For instance, the medical devices can be coated with PURASORB/CBD, a methodology that has already been studied in other settings [74,75,76]. Alternatively, CBD can be incorporated into PLGA-based medical devices such as nasal stents, sutures, and Polyglactin 910 meshes.

The incorporation of CBD into PLGA nano- or microparticles has been studied to improve the pharmacokinetics of CBD for various medical purposes such as treating inflammatory conditions, cancer, neurodegenerative diseases, and pain [77,78,79,80] and to form in situ sustained-release implants [81]. A study by David et al. [67] showed that PLGA microparticles (65:35 ratio) containing 5–10% CBD oil embedded in a chondroitin sulfate/polyvinyl alcohol hydrogel matrix prevented the growth of *S. aureus* in a disk diffusion assay for two subsequent days without affecting the viability of primary human dental pulp cells. Thereafter, the antibacterial activity was lost, suggesting that most of the CBD was released during the first two days [67]. Qi et al. [82] prepared a CBD-loaded alginate–copper hydrogel intended for bone repair, which significantly reduced the bacterial growth of *S. aureus* (MSSA) and *Escherichia coli* after a 24 h incubation. Part of the antibacterial activity was related to the copper ions that were released during this time period [82]. Notably, CBD was found to induce osteogenesis of human dental pulp cells [83], suggesting that it might have positive effects on oral health. Together with our data showing an antibacterial activity of the PURASORB/CBD scaffolds on the cariogenic bacterium *S. mutans* and an anti-inflammatory activity on macrophages, these devices might have a beneficial effect in preventing periodontal inflammatory diseases. No less important is the treatment of infection caused by *S. aureus*, which can occur in the nasal cavity, bone, and skin. The CBD scaffold is expected to be useful in the prevention and treatment of *S. aureus* infections after surgical procedures. It could be used as a nasal stent, similar to the biodegradable PROPEL and SINUVA sinus implants that contain mometasone, which has an anti-inflammatory effect. Another prospect is the incorporation of CBD into the biodegradable Polyglactin 910 meshes used after hernia surgery, which should reduce post-operative *S. aureus* infections as well as inflammation. Similarly, CBD scaffolds can be used to cover skin infections caused by *S. aureus*. The wound-healing properties of CBD would further contribute to a faster recovery [84,85,86]. In dentistry, CBD scaffolds can be used as chips inserted into periodontal pockets, where it is expected to reduce bacterial infection and inflammation. CBD has been shown to increase the wound healing activity of human gingival fibroblasts [87], reduce bone resorption [88], and alleviate gingivitis and periodontitis [64,65].

It should be kept in mind that the PURASORB/CBD drug delivery system is only active when they are embedded in fluid, which is usually the case in the infected area. Further studies should be conducted to investigate whether encapsulating the PURASORB/CBD devices with a hydrogel would increase their effectiveness against bacteria. Various hydrogels have been studied for embedding polymer/CBD nano- or microparticles [67,78,79,89,90]. These include a chondroitin sulfate/polyvinyl alcohol matrix [67], chitosan [78,79], gelatin/nano-hydroxyapatite [89], and hydroxypropyl cellulose [90].

The novelty of this study is the design of a novel PURASORB/CBD sustained-release device that can be used to treat local infections caused by the CBD-susceptible bacteria *S. mutans* and *S. aureus*, including antibiotic-resistant *S. aureus*. Importantly, the incorporated drug was released in small portions over an extended period of time, which allowed the generation of a persistent antibacterial environment. This device also had an anti-inflammatory effect, without causing any significant cytotoxic effect on mammalian cells. The limitation of this study is that it is an in vitro study, and further in vivo studies are required to prove its medical applicability. Further studies should be conducted to investigate the stability of the scaffolds over time. Our experience so far shows that CBD is biologically active as long as the scaffolds are protected from light. Since the scaffolds are stored dry, it is recommended to rehydrate the CBD scaffolds before use to initiate the polymer decomposition process. Our data show that the CBD-containing scaffolds are still active after 17 days in terms of antibacterial, antibiofilm, and anti-inflammatory effects.

## 5. Conclusions

We have developed a sustained-release CBD technology that exhibits prolonged antibacterial and antibiofilm activity against *S. mutans* and *S. aureus*, as well as anti-inflammatory activity as measured by the reduced IL-6 secretion by LPS-stimulated macrophages. The device is based on the biodegradable polymers PURASORB 5010 and PURASORB 7510, with PURASORB 5010 performing better than PURASORB 7510 in terms of the sustained antibacterial activity in the microenvironment. The presence of PEG400 enhanced the antibacterial and antibiofilm activity of the PURASORB 7510/CBD scaffolds, while the effect on the POLYSORB 5010/CBD scaffolds was relatively low. Both polymers released a sufficient amount of CBD daily to achieve the anti-inflammatory effect over an extended period of time. The amount of CBD released daily from the devices was below the cytotoxic dose for normal Vero epithelial cells, which is an advantage for minimizing the cytotoxic effects. Although this is an in vitro study, it is a proof of principle that the incorporation of CBD into these polymers is a feasible approach for the safe delivery of CBD with long-lasting effects.

## Data Availability

Raw data are available upon reasonable request.

## Supplementary Materials

The following supporting information can be downloaded at https://www.mdpi.com/article/10.3390/pharmaceutics17040463/s1, Supplementary Figure S1. Effect of solvents on the antibacterial activity of CBD. Supplementary Figure S2. HR-SEM images of PURASORB 5010 and PURASORB 7510 scaffolds with or without CBD after five times of exposure to fresh *S. mutans* cultures. Supplementary Figure S3. HR-SEM images of PURASORB 5010 and PURASORB 7510 scaffolds with or without CBD after seven times of exposure to fresh MDRSA CI-M cultures. Supplementary Figure S4. HR-SEM images of PURASORB 5010 and PURASORB 7510 scaffolds with or without CBD after 13 times of exposure to fresh *S. aureus* ATCC25923 cultures. Supplementary Figure S5. Metabolic activity of RAW 264.7 macrophages after 6 h incubation with the indicated scaffold-conditioned media. Supplementary Figures S6 and S7. Cytotoxicity assay of scaffold-conditioned media using Vero epithelial cells. Full-size HR-SEM images of the individual images of Figure 3, Figure 5, Figure 7, Figure 10 and Figure 12. Certificate of Analysis of CBD. Supplementary File S2: Examples of chromatograms for determining the CBD content in scaffold-conditioned media.
