# Supplementary material for: The Incorporation of CBD into Biodegradable DL-Lactide/Glycolide Copolymers Creates a Persistent Antibacterial Environment: An In Vitro Study on Streptococcus mutans and Staphylococcus aureus
Source: Pharmaceutics. 2025 Apr 2;17(4):463. doi: 10.3390/pharmaceutics17040463 (PMC12030335; doi:10.3390/pharmaceutics17040463)
Supplement: Supplementary file 1 [file pharmaceutics-17-00463-s001.zip › pharmaceutics-3548672-supplementary/pharmaceutics-3548672-supplementary.pptx]

## Slide 1
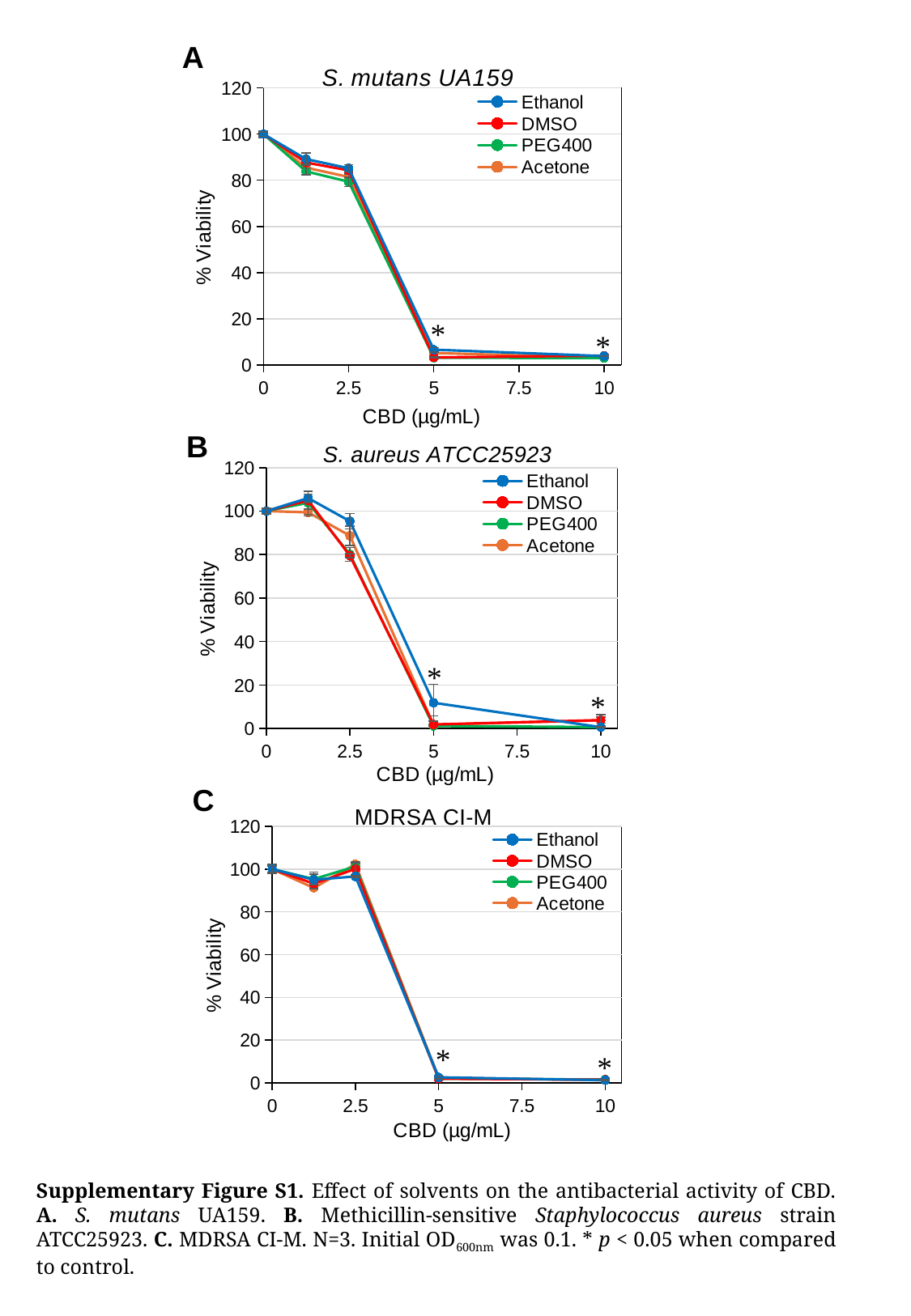

A
### Chart: S. mutans UA159
| Category | | | | |
|---|---|---|---|---|B
### Chart: S. aureus ATCC25923
| Category | | | | |
|---|---|---|---|---|C
### Chart: MDRSA CI-M
| Category | | | | |
|---|---|---|---|---|*
*
*
*
*
*
Supplementary Figure S1. Effect of solvents on the antibacterial activity of CBD. A. S. mutans UA159. B. Methicillin-sensitive Staphylococcus aureus strain ATCC25923. C. MDRSA CI-M. N=3. Initial OD600nm was 0.1. * p < 0.05 when compared to control.

## Slide 2
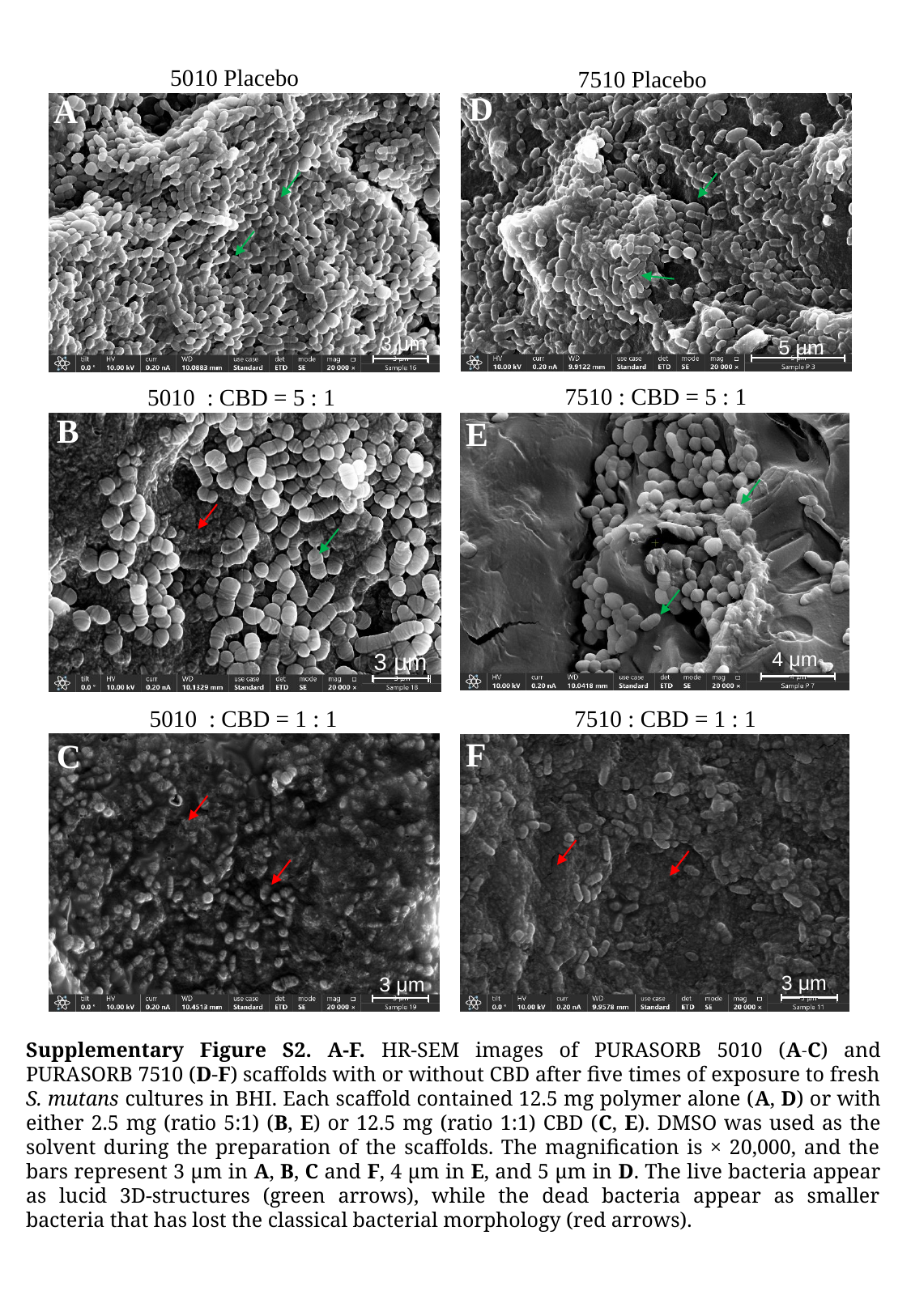

5010 Placebo
7510 Placebo
D
A
7510 : CBD = 5 : 1
5010 : CBD = 5 : 1
B
E
5010 : CBD = 1 : 1
7510 : CBD = 1 : 1
F
C
3 μm
5 μm
4 μm
3 μm
3 μm
3 μm
Supplementary Figure S2. A-F. HR-SEM images of PURASORB 5010 (A˗C) and PURASORB 7510 (D-F) scaffolds with or without CBD after five times of exposure to fresh S. mutans cultures in BHI. Each scaffold contained 12.5 mg polymer alone (A, D) or with either 2.5 mg (ratio 5:1) (B, E) or 12.5 mg (ratio 1:1) CBD (C, E). DMSO was used as the solvent during the preparation of the scaffolds. The magnification is × 20,000, and the bars represent 3 μm in A, B, C and F, 4 μm in E, and 5 μm in D. The live bacteria appear as lucid 3D-structures (green arrows), while the dead bacteria appear as smaller bacteria that has lost the classical bacterial morphology (red arrows).

## Slide 3
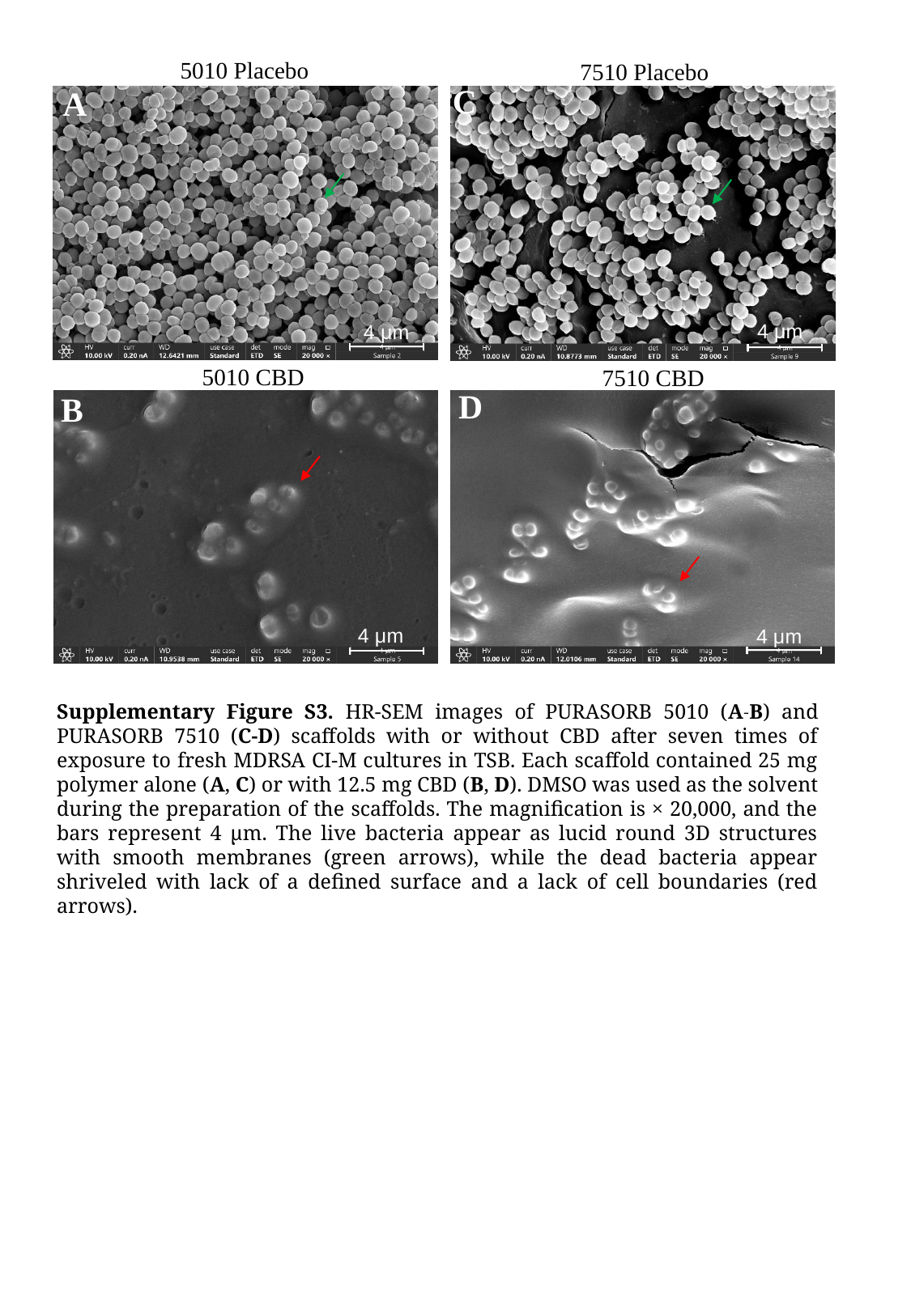

5010 Placebo
7510 Placebo
C
A
5010 CBD
7510 CBD
D
B
4 μm
4 μm
4 μm
4 μm
Supplementary Figure S3. HR-SEM images of PURASORB 5010 (A˗B) and PURASORB 7510 (C-D) scaffolds with or without CBD after seven times of exposure to fresh MDRSA CI-M cultures in TSB. Each scaffold contained 25 mg polymer alone (A, C) or with 12.5 mg CBD (B, D). DMSO was used as the solvent during the preparation of the scaffolds. The magnification is × 20,000, and the bars represent 4 μm. The live bacteria appear as lucid round 3D structures with smooth membranes (green arrows), while the dead bacteria appear shriveled with lack of a defined surface and a lack of cell boundaries (red arrows).

## Slide 4
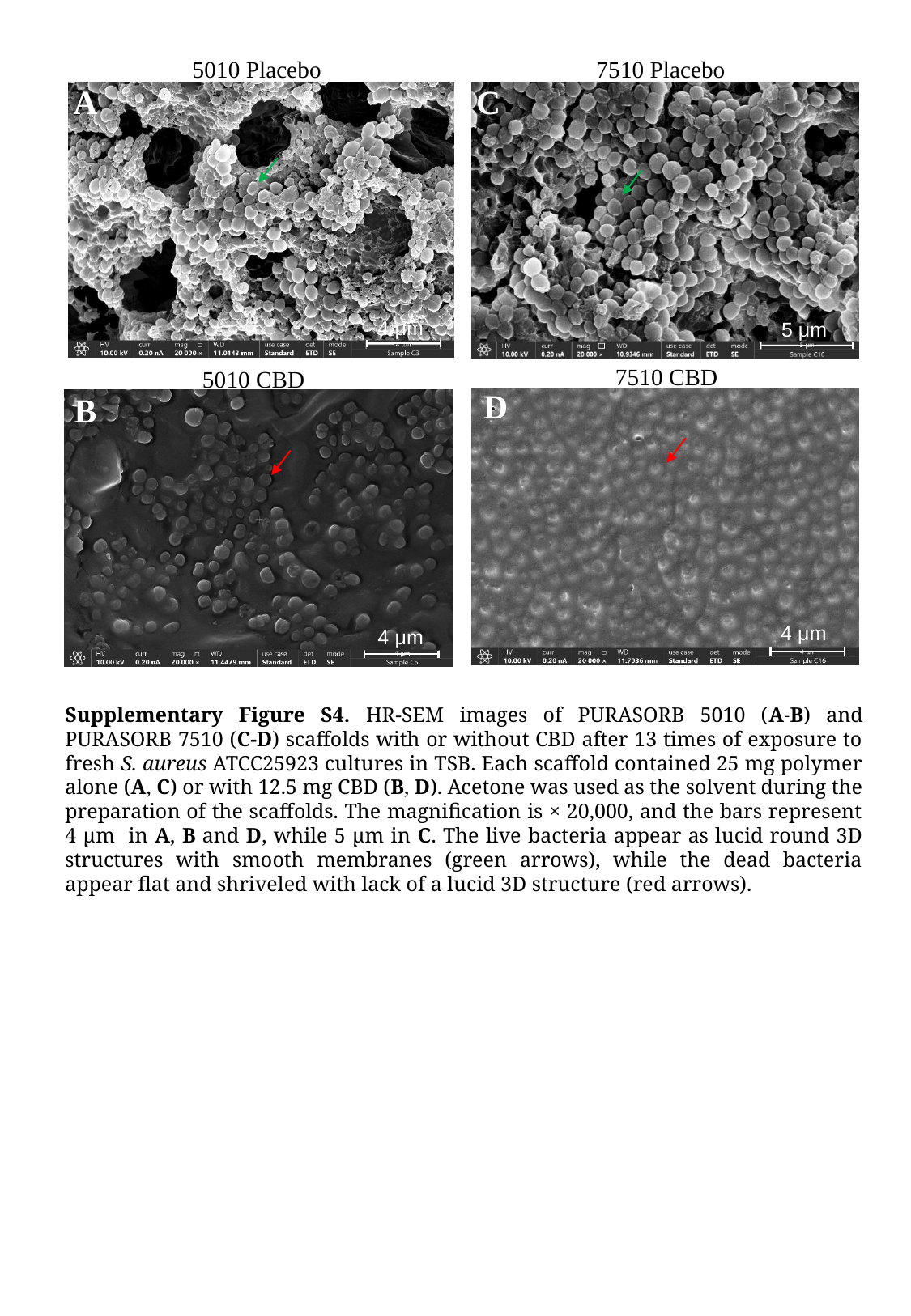

7510 Placebo
5010 Placebo
A
C
4 μm
5 μm
7510 CBD
5010 CBD
D
B
4 μm
4 μm
Supplementary Figure S4. HR-SEM images of PURASORB 5010 (A˗B) and PURASORB 7510 (C-D) scaffolds with or without CBD after 13 times of exposure to fresh S. aureus ATCC25923 cultures in TSB. Each scaffold contained 25 mg polymer alone (A, C) or with 12.5 mg CBD (B, D). Acetone was used as the solvent during the preparation of the scaffolds. The magnification is × 20,000, and the bars represent 4 μm in A, B and D, while 5 μm in C. The live bacteria appear as lucid round 3D structures with smooth membranes (green arrows), while the dead bacteria appear flat and shriveled with lack of a lucid 3D structure (red arrows).

## Slide 5
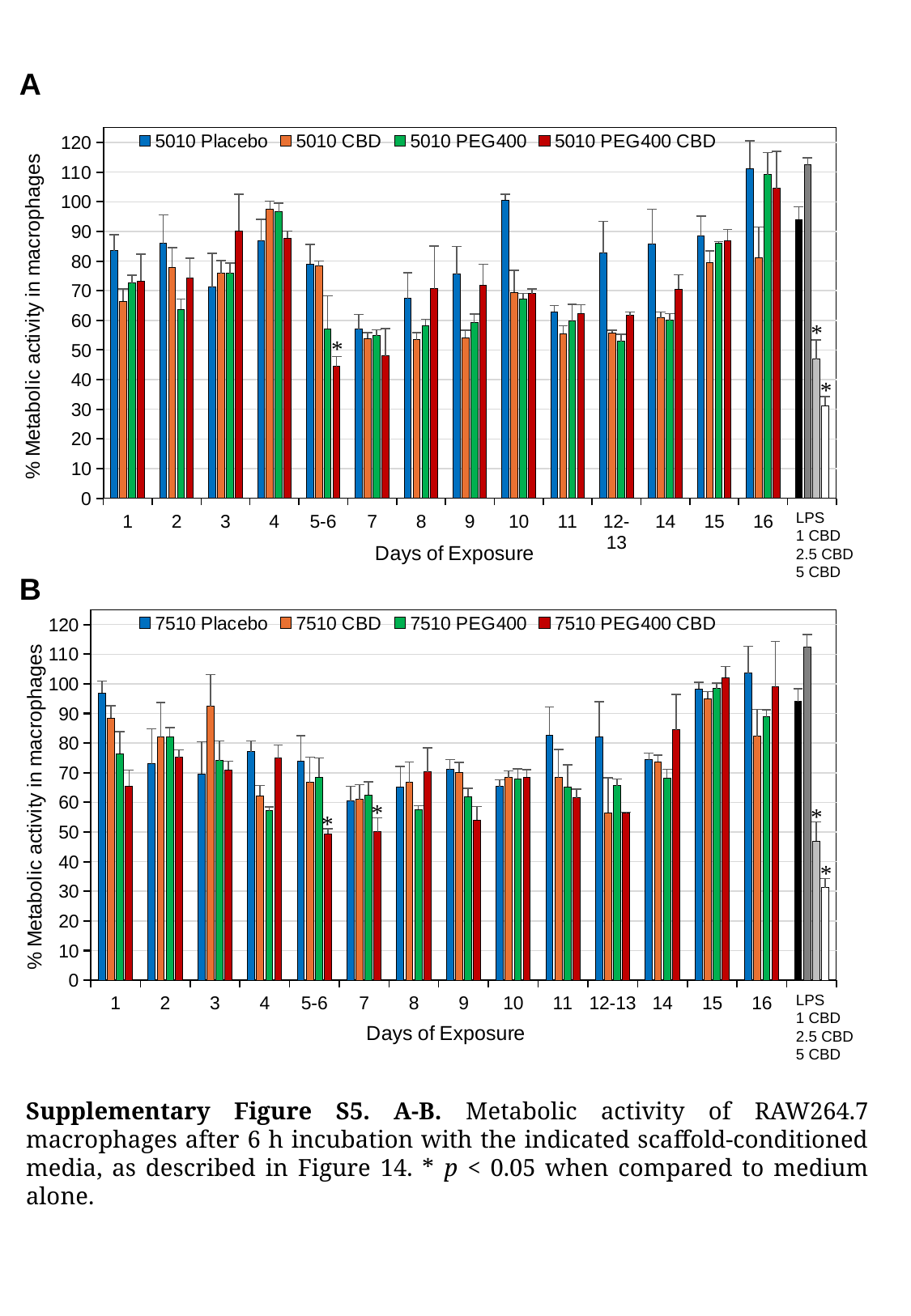

A
### Chart
| Category | | | | |
|---|---|---|---|---|
| 1 | 83.54073101024268 | 66.44418014307777 | 72.57912768714611 | 73.21818472298654 |
| 2 | 85.95317132054036 | 77.85312339126268 | 63.69268457209807 | 74.18564606891167 |
| 3 | 71.23888307031403 | 75.8702714217244 | 75.89689879821776 | 90.26858147089628 |
| 4 | 86.74311682317648 | 97.62306285836011 | 96.60412191788119 | 87.73010491186339 |
| 5-6 | 79.0371540660004 | 78.26851046455899 | 57.17075248965971 | 44.49967159568992 |
| 7 | 57.0305149734614 | 53.86008201231961 | 54.90565032929189 | 47.975431807288814 |
| 8 | 67.35128610228465 | 53.465996840218 | 58.276676193350255 | 70.6885839561181 |
| 9 | 75.55961869596864 | 54.13345641098468 | 59.238812063976724 | 71.88326558145316 |
| 10 | 100.63195640210891 | 69.41934567660164 | 67.28027976496904 | 69.06786430688939 |
| 11 | 62.94179255498554 | 55.46837555251807 | 59.77668506914243 | 62.293859726980635 |
| 12-13 | 82.6922052793212 | 55.72577352528714 | 52.8908455079616 | 61.71693323629135 |
| 14 | 85.73837715016067 | 60.88793424813166 | 60.04828430937464 | 70.48799105320151 |
| 15 | 88.42064154225766 | 79.56437612056877 | 86.09695915360447 | 86.93660909236151 |
| 16 | 111.24385351392614 | 81.12119006621344 | 109.22727353416293 | 104.59411002431969 |LPS
1 CBD
2.5 CBD
5 CBD
B
### Chart
| Category | | | | |
|---|---|---|---|---|
| 1 | 96.89879821774096 | 88.46147018621413 | 76.36909094136651 | 65.56547671879717 |
| 2 | 73.07617204835533 | 82.05314824348075 | 82.13658068982657 | 75.14245646423946 |
| 3 | 69.66431754034049 | 92.34019136207907 | 74.03830791898177 | 70.91047876022937 |
| 4 | 77.12175811691195 | 62.173148953544114 | 57.23288303481087 | 74.91878650169528 |
| 5-6 | 73.93002325457547 | 66.6909271652495 | 68.50513908366322 | 49.377806969272015 |
| 7 | 60.43526884774466 | 60.94118900111836 | 62.54060674915236 | 50.05059201533738 |
| 8 | 65.21044503221913 | 66.7299806507731 | 57.6393943159427 | 70.23591855573112 |
| 9 | 71.04894111799479 | 70.18443896117729 | 61.97078089219463 | 54.08020165799798 |
| 10 | 65.38973603394103 | 68.35780093373334 | 67.9335380682726 | 68.58502121314328 |
| 11 | 82.73835939857634 | 68.32762324037421 | 65.24594820087694 | 61.53941739300235 |
| 12-13 | 82.17740933378305 | 56.54057124598372 | 65.64535884827721 | 56.36483056112759 |
| 14 | 74.4323930910834 | 73.74363161912201 | 68.25129142775994 | 84.48334013810734 |
| 15 | 98.2727708447979 | 95.03665702163919 | 98.40945804413046 | 102.13906591163263 |
| 16 | 103.54321623204874 | 82.30344558251825 | 88.80585092219482 | 98.98460937638686 |LPS
1 CBD
2.5 CBD
5 CBD
*
*
*
*
*
*
*
Supplementary Figure S5. A-B. Metabolic activity of RAW264.7 macrophages after 6 h incubation with the indicated scaffold-conditioned media, as described in Figure 14. * p < 0.05 when compared to medium alone.

## Slide 6
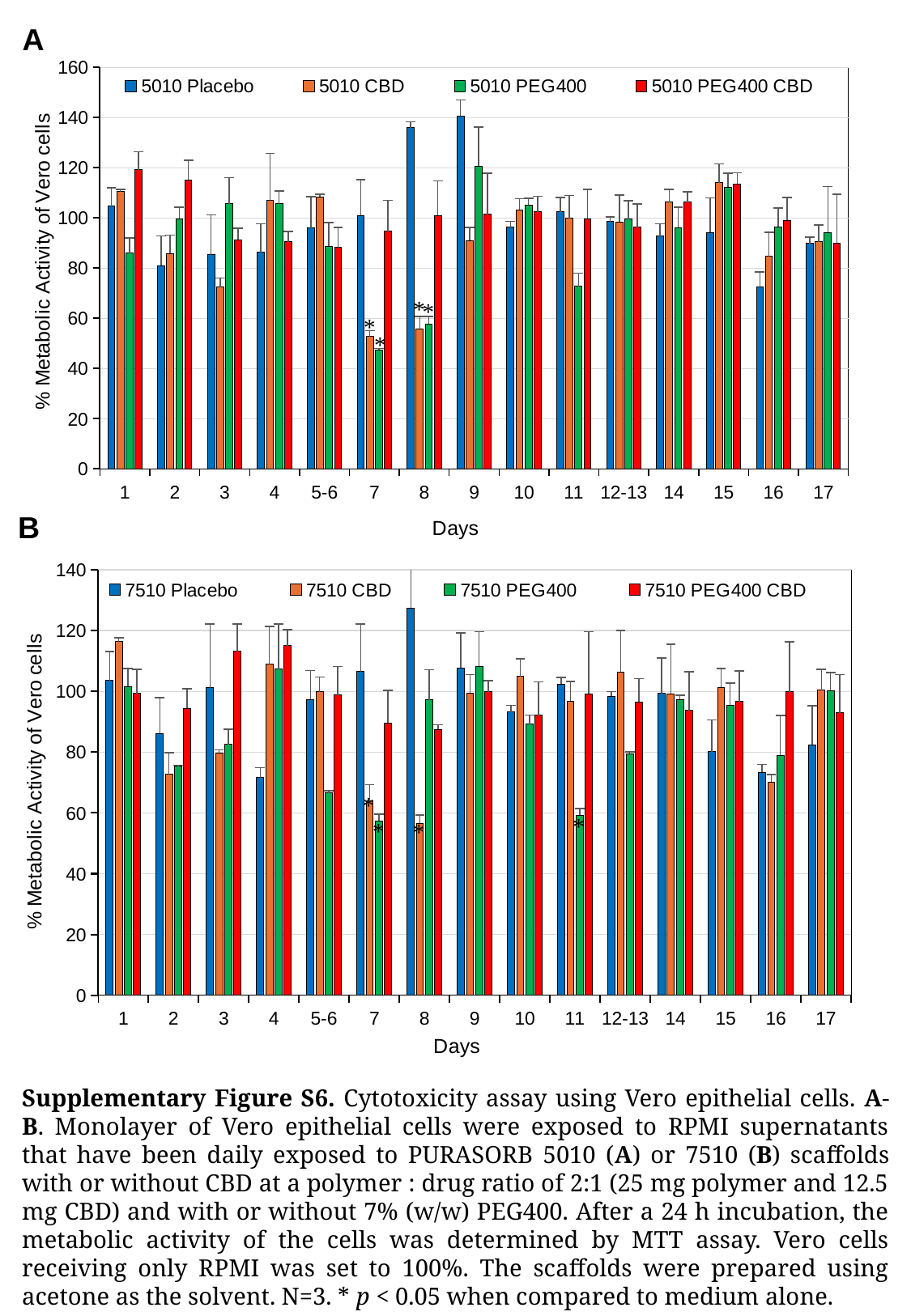

A
### Chart
| Category | 5010 Placebo | 5010 CBD | 5010 PEG400 | 5010 PEG400 CBD |
|---|---|---|---|---|
| 1 | 104.72053609548738 | 110.44161051400567 | 85.92934974290435 | 119.18193462159606 |
| 2 | 81.07516103283768 | 85.87455978534297 | 99.77373571155711 | 115.13310702161871 |
| 3 | 85.43743386980007 | 72.43647402743423 | 105.70853597182628 | 91.3822881305461 |
| 4 | 86.35443930871897 | 106.99362346096206 | 105.62468556095925 | 90.46240092071801 |
| 5-6 | 95.9551521226634 | 108.38504108670134 | 88.73976722169627 | 88.28868964749122 |
| 7 | 101.05689228693502 | 52.83337081178322 | 47.413986957499446 | 94.74008991801593 |
| 8 | 136.02990780301326 | 55.79042050820778 | 57.70182145266472 | 101.07328525909777 |
| 9 | 140.67910951203058 | 91.05014616595457 | 120.7105914099393 | 101.43529271633611 |
| 10 | 96.41087041265244 | 103.31571608447526 | 105.21097931879528 | 102.51990867071336 |
| 11 | 102.49184567012428 | 99.81049807144055 | 72.82437598380932 | 99.6445386860065 |
| 12-13 | 98.51044541057045 | 98.4169042428308 | 99.56150213627164 | 96.37914812612013 |
| 14 | 92.77997438324887 | 106.372631225893 | 96.10208339455201 | 106.38060020647386 |
| 15 | 94.17218911844964 | 114.06693768446843 | 112.28211839394106 | 113.56574037979617 |
| 16 | 72.51953741345065 | 84.70129675436624 | 96.50326062514054 | 99.06408862507713 |
| 17 | 89.83404800356408 | 90.46923019263924 | 94.232066561727 | 90.07546536978032 |*
*
*
*
B
### Chart
| Category | 7510 Placebo | 7510 CBD | 7510 PEG400 | 7510 PEG400 CBD |
|---|---|---|---|---|
| 1 | 103.5436505726643 | 116.43207663600835 | 101.65209481910489 | 99.35401236286683 |
| 2 | 86.16324181841807 | 72.63698373435275 | 75.38415411138595 | 94.41036534122112 |
| 3 | 101.22143639434947 | 79.82535042350649 | 82.54253804062664 | 113.15585730450834 |
| 4 | 71.59325054296374 | 108.98695729855277 | 107.27150763038738 | 115.02723019607063 |
| 5-6 | 97.40274342253205 | 99.88756465032607 | 66.54298778202534 | 98.81284710998794 |
| 7 | 106.49314144366988 | 63.96447042950303 | 57.342028333708114 | 89.5659995502586 |
| 8 | 127.41736001798967 | 56.58871149089274 | 97.26782100292331 | 87.47470204632337 |
| 9 | 107.7407139277162 | 99.46333684050316 | 108.30789870870368 | 99.92359907143485 |
| 10 | 93.21817876907598 | 104.93079838970351 | 89.28491117607376 | 92.19802862393495 |
| 11 | 102.33115958323961 | 96.81807960422756 | 59.180346300877 | 99.27011163387792 |
| 12-13 | 98.4575069977964 | 106.4011403655878 | 79.54426204932163 | 96.525254931544 |
| 14 | 99.40413209333407 | 99.18146429374191 | 97.3166842025159 | 93.73688997791018 |
| 15 | 80.1748621707412 | 101.30826472419423 | 95.54523816520232 | 96.74627280487562 |
| 16 | 73.31402795567188 | 70.10344052170004 | 78.95585038602803 | 99.86080831796077 |
| 17 | 82.47851348592008 | 100.35604527396748 | 100.32606251405443 | 92.88284236563976 |*
*
*
*
Supplementary Figure S6. Cytotoxicity assay using Vero epithelial cells. A-B. Monolayer of Vero epithelial cells were exposed to RPMI supernatants that have been daily exposed to PURASORB 5010 (A) or 7510 (B) scaffolds with or without CBD at a polymer : drug ratio of 2:1 (25 mg polymer and 12.5 mg CBD) and with or without 7% (w/w) PEG400. After a 24 h incubation, the metabolic activity of the cells was determined by MTT assay. Vero cells receiving only RPMI was set to 100%. The scaffolds were prepared using acetone as the solvent. N=3. * p < 0.05 when compared to medium alone.

## Slide 7
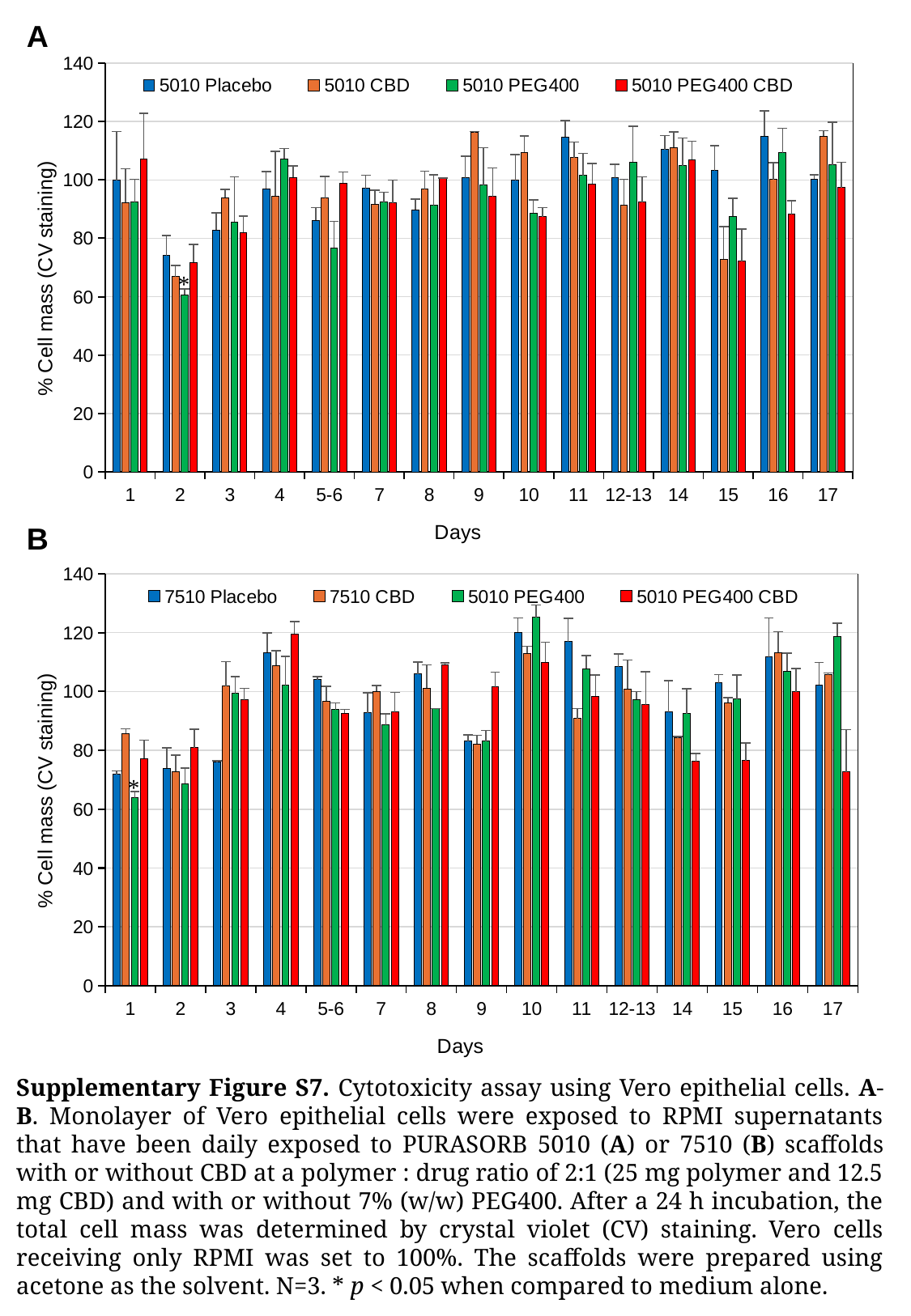

A
### Chart
| Category | | | | |
|---|---|---|---|---|
| 1 | 99.8805256869773 | 92.29434930749147 | 92.36986887325398 | 107.23306340988539 |
| 2 | 74.11065386374028 | 66.93747510951813 | 60.70357095446701 | 71.75538740652242 |
| 3 | 82.8284437364485 | 93.86374028349339 | 85.60378777822028 | 82.0354882959423 |
| 4 | 96.9647624526159 | 94.53397642963553 | 107.23542339631547 | 100.90357980441613 |
| 5-6 | 86.16782453500893 | 93.71270115196837 | 76.77507854329838 | 98.86101154918359 |
| 7 | 97.17716123132291 | 91.73503252356299 | 92.3557089546735 | 92.1857899317079 |
| 8 | 89.72078410549139 | 96.85384309040224 | 91.32675487115948 | 100.41152263374487 |
| 9 | 100.72186084930013 | 116.2918713217399 | 98.19431538268655 | 94.29797778662773 |
| 10 | 99.89114562591266 | 109.48921043703999 | 88.61041049013969 | 87.34309777718777 |
| 11 | 114.73428027788842 | 107.58234140153695 | 101.4864964526454 | 98.5766331843592 |
| 12-13 | 100.73130079502045 | 91.39755446406183 | 106.06133014735167 | 92.32974910394265 |
| 14 | 110.35768544330871 | 111.13412097880435 | 104.91555673554878 | 106.93334513326549 |
| 15 | 103.15854683835568 | 72.8740209743794 | 87.60387627771141 | 72.16838503178606 |
| 16 | 115.02809858843312 | 100.11534433677008 | 109.3535112173105 | 88.22691269525201 |
| 17 | 100.35488295942298 | 115.02809858843312 | 105.07603581279407 | 97.38956001002994 |*
B
### Chart
| Category | | | | |
|---|---|---|---|---|
| 1 | 71.82382701299467 | 85.7099871675738 | 63.847072879330945 | 77.27067569361478 |
| 2 | 73.87229523430241 | 72.80676136112218 | 68.63784533238935 | 80.94517456524625 |
| 3 | 75.94672330634099 | 101.83105447143679 | 99.36722863843532 | 97.1559213534522 |
| 4 | 113.22624894906855 | 108.87089399235954 | 102.11897281590629 | 119.52387273773178 |
| 5-6 | 104.07304158001094 | 96.74056374175846 | 93.80002064988126 | 92.63890732628288 |
| 7 | 92.7002669734649 | 100.05398468958803 | 88.57737068011859 | 93.07786480227739 |
| 8 | 105.9657506969335 | 101.16317831172472 | 94.14221868224257 | 109.09509270321695 |
| 9 | 83.17536174166999 | 82.18416744103722 | 83.15176187736921 | 101.61865569272976 |
| 10 | 120.13274923669191 | 112.94895054353438 | 125.18784016991901 | 109.92108795374426 |
| 11 | 116.91844771892563 | 90.85239759871382 | 107.7121406551912 | 98.44211395784474 |
| 12-13 | 108.40125669277401 | 100.83278021151378 | 97.30460049854713 | 95.48033098809682 |
| 14 | 93.05190495154653 | 84.1523961237223 | 92.50674808619851 | 76.17328200362849 |
| 15 | 102.91664822927267 | 96.2213667271413 | 97.42023983362095 | 76.52727996814018 |
| 16 | 111.75007743705474 | 113.1920291458324 | 106.9215452011151 | 99.89350561234272 |
| 17 | 102.26529197457114 | 105.64715252887298 | 118.7025974600646 | 72.65454223638214 |*
Supplementary Figure S7. Cytotoxicity assay using Vero epithelial cells. A-B. Monolayer of Vero epithelial cells were exposed to RPMI supernatants that have been daily exposed to PURASORB 5010 (A) or 7510 (B) scaffolds with or without CBD at a polymer : drug ratio of 2:1 (25 mg polymer and 12.5 mg CBD) and with or without 7% (w/w) PEG400. After a 24 h incubation, the total cell mass was determined by crystal violet (CV) staining. Vero cells receiving only RPMI was set to 100%. The scaffolds were prepared using acetone as the solvent. N=3. * p < 0.05 when compared to medium alone.

## Slide 8
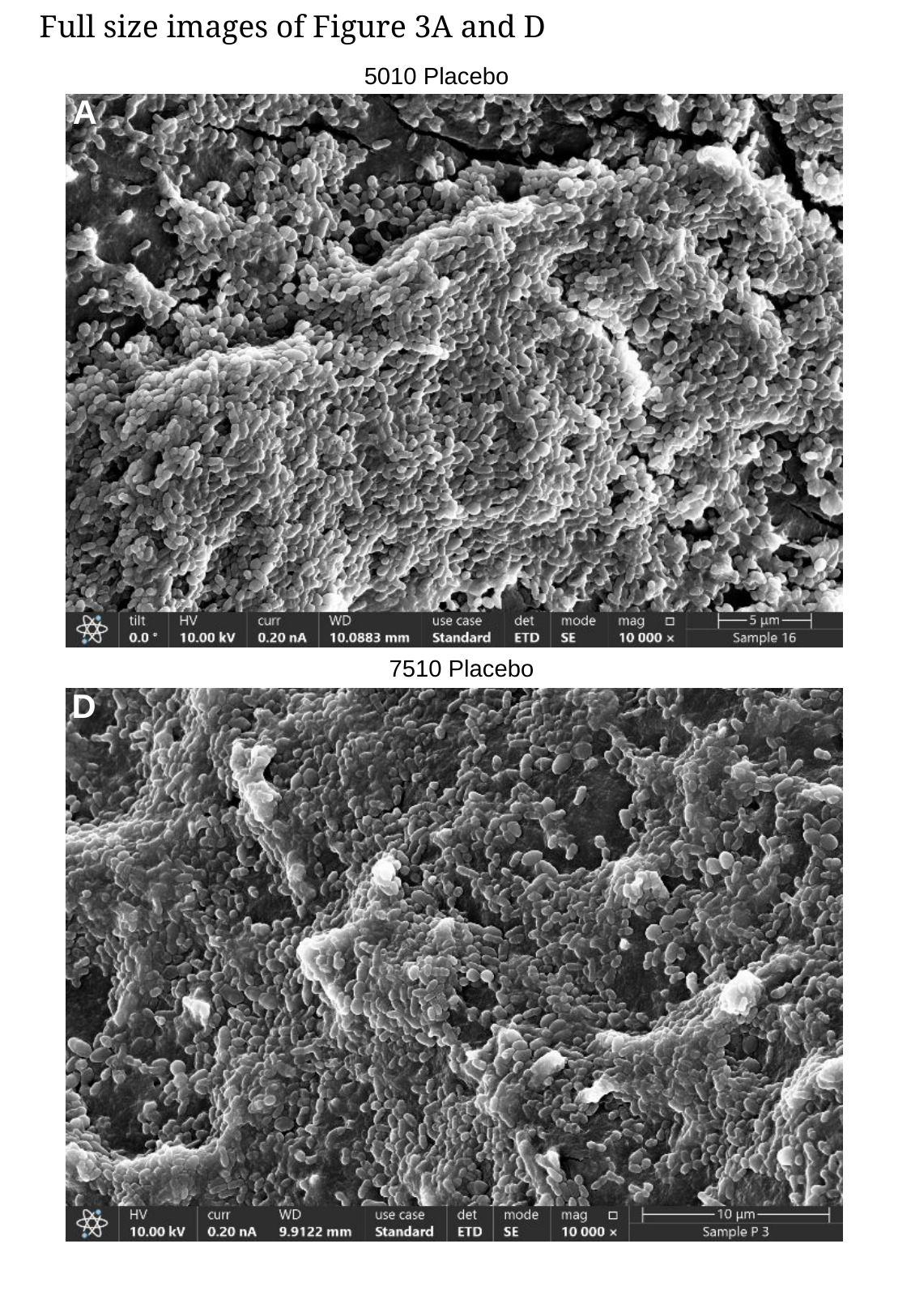

Full size images of Figure 3A and D
5010 Placebo
A
7510 Placebo
D

## Slide 9
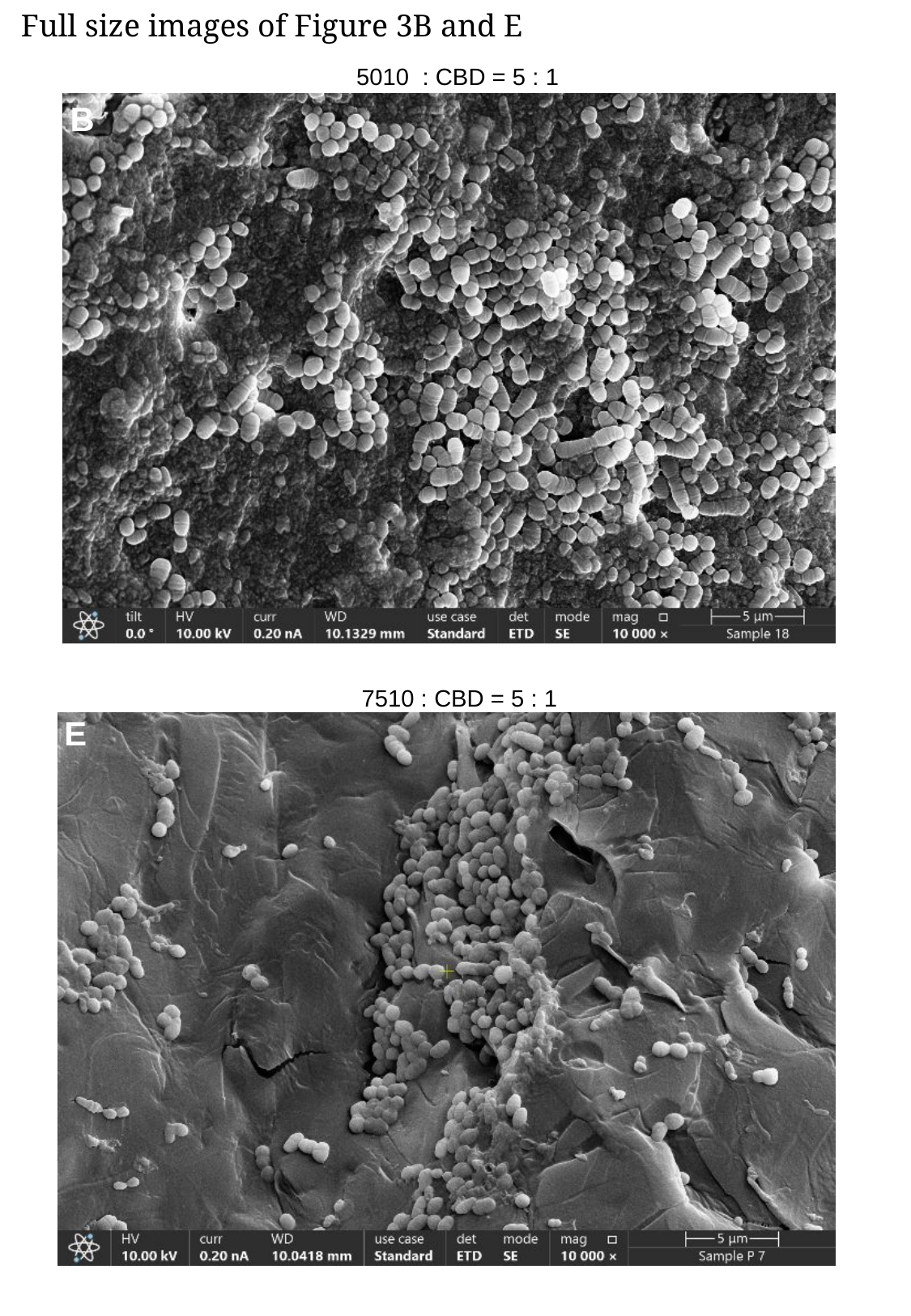

Full size images of Figure 3B and E
5010 : CBD = 5 : 1
B
7510 : CBD = 5 : 1
E

## Slide 10
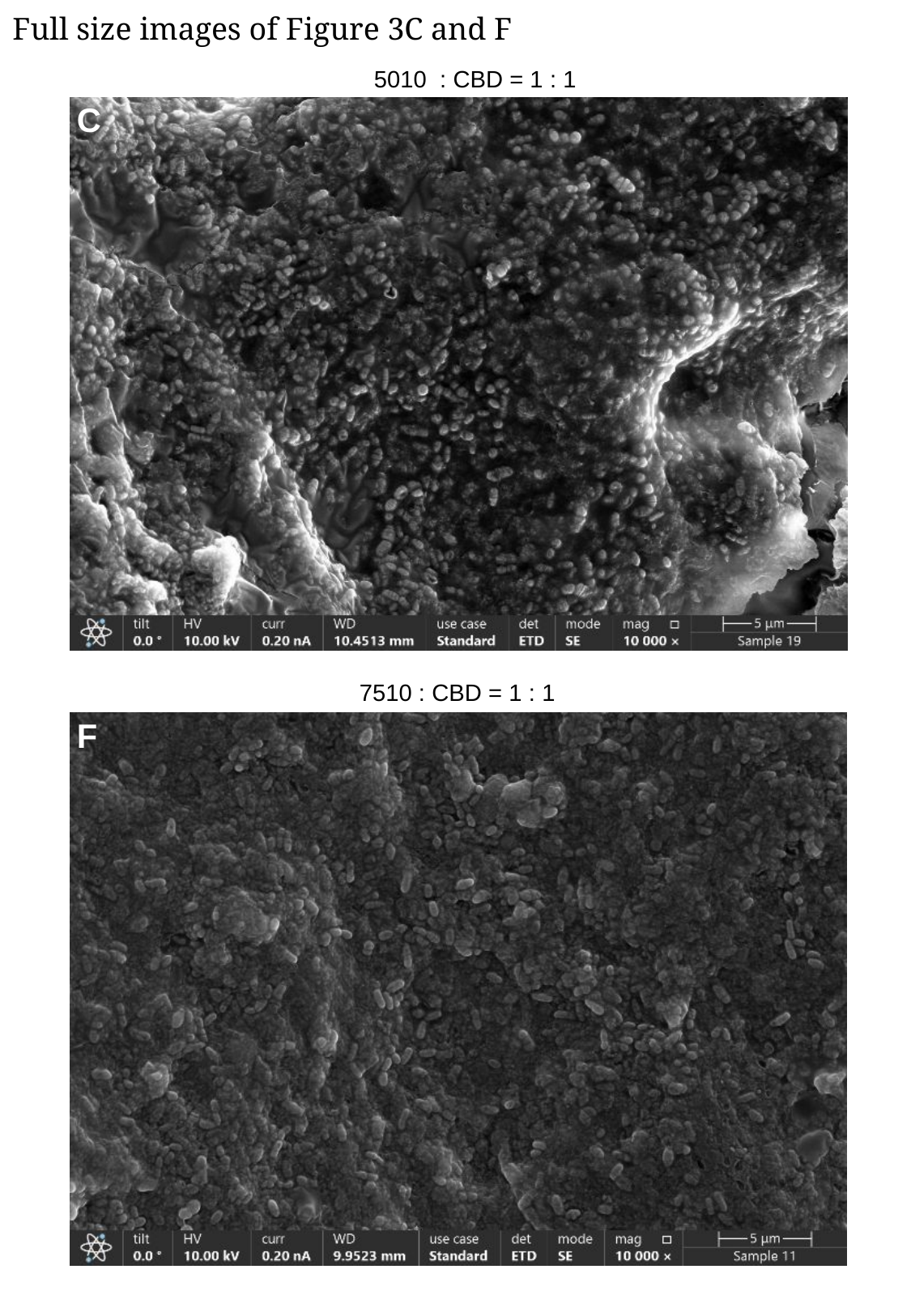

Full size images of Figure 3C and F
5010 : CBD = 1 : 1
C
7510 : CBD = 1 : 1
F

## Slide 11
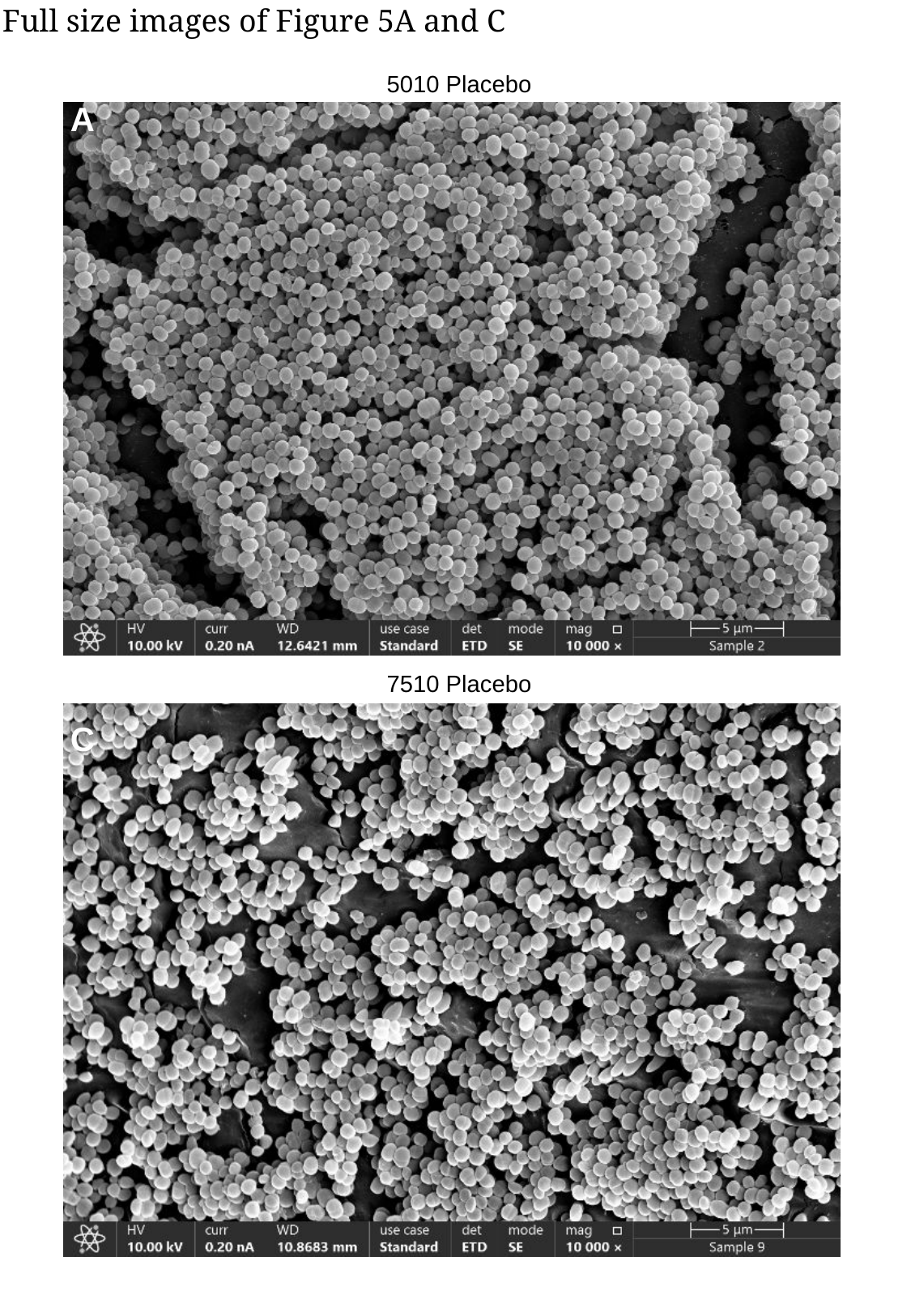

Full size images of Figure 5A and C
5010 Placebo
A
7510 Placebo
C

## Slide 12
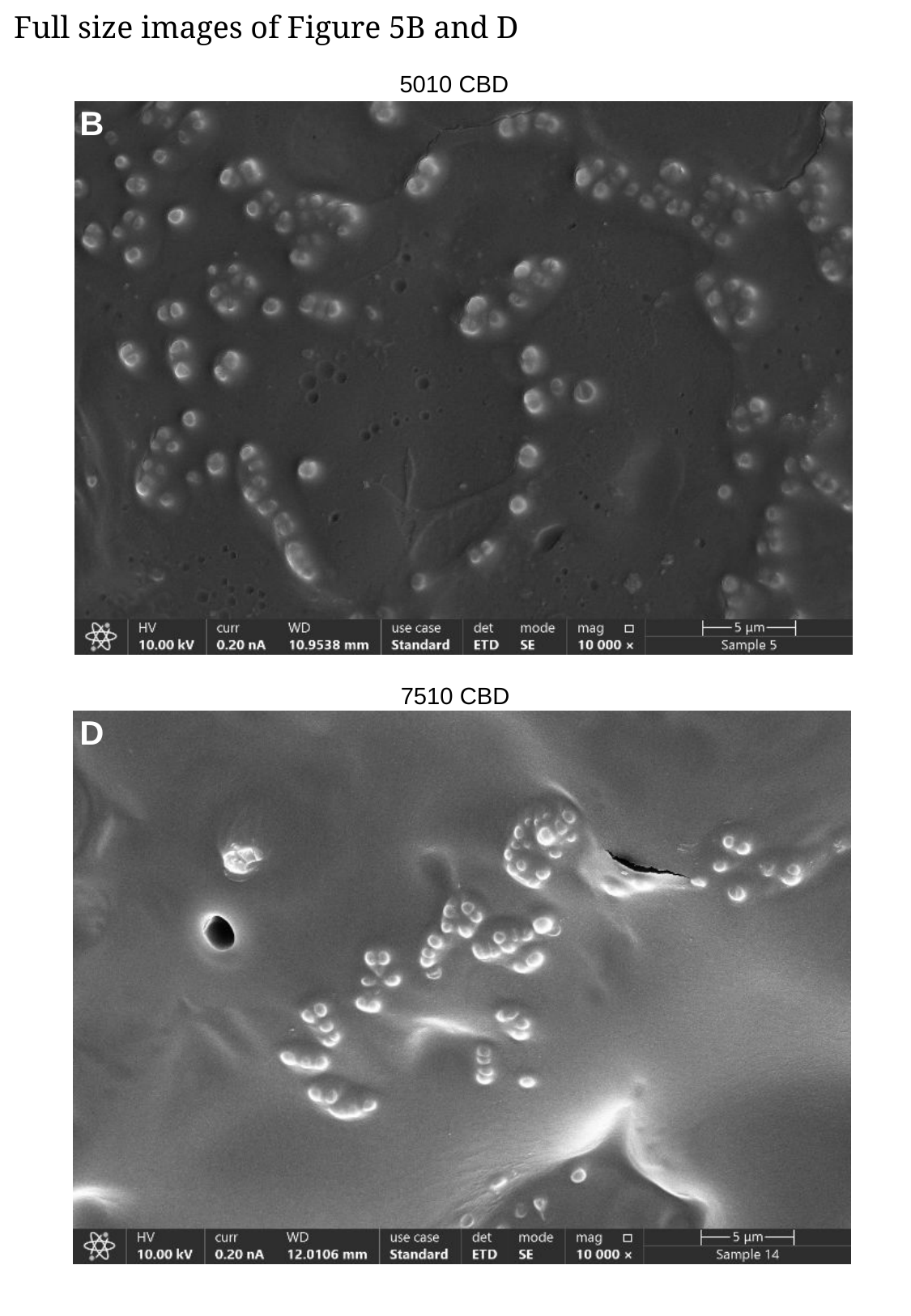

Full size images of Figure 5B and D
5010 CBD
B
7510 CBD
D

## Slide 13
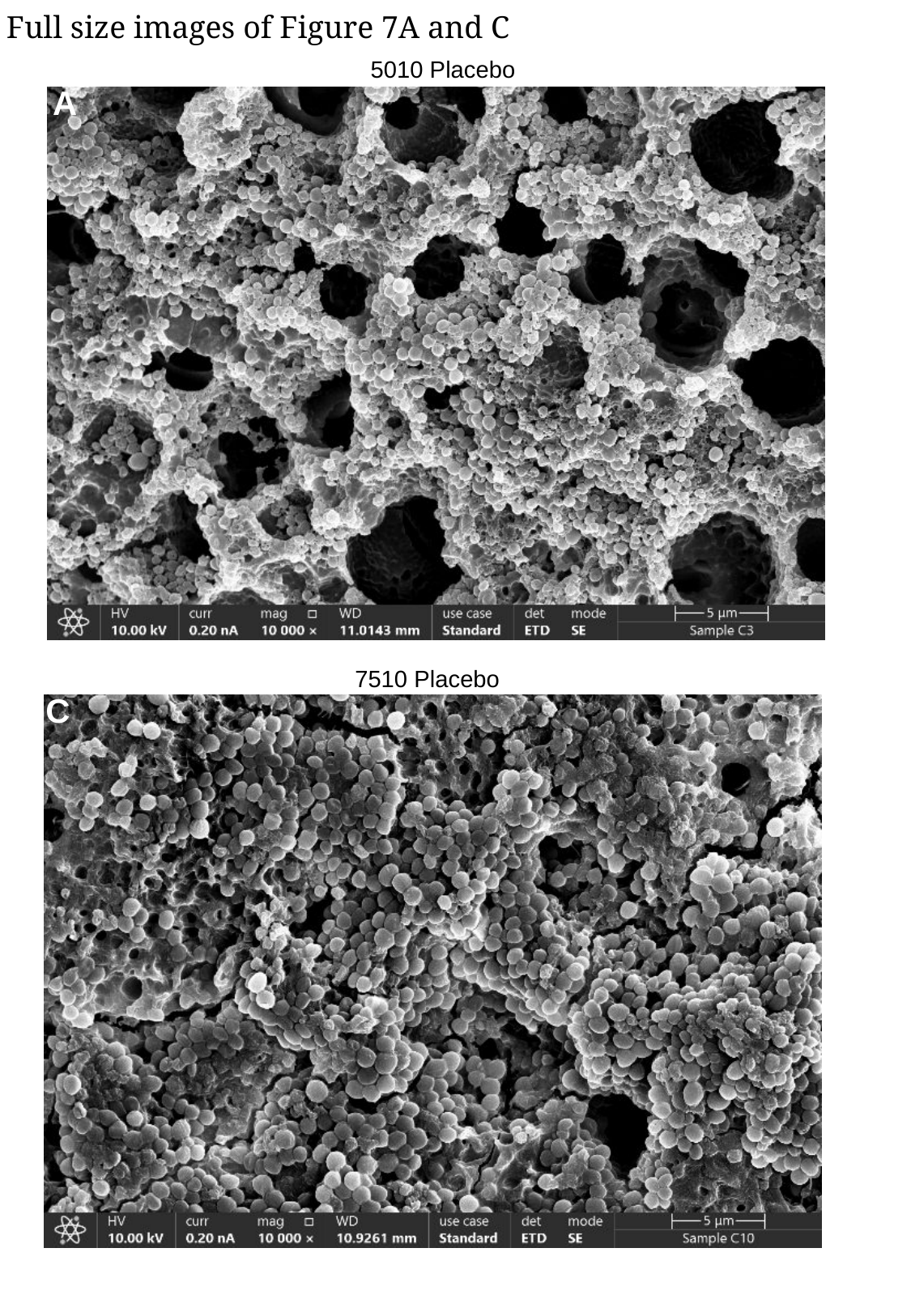

Full size images of Figure 7A and C
5010 Placebo
A
7510 Placebo
C

## Slide 14
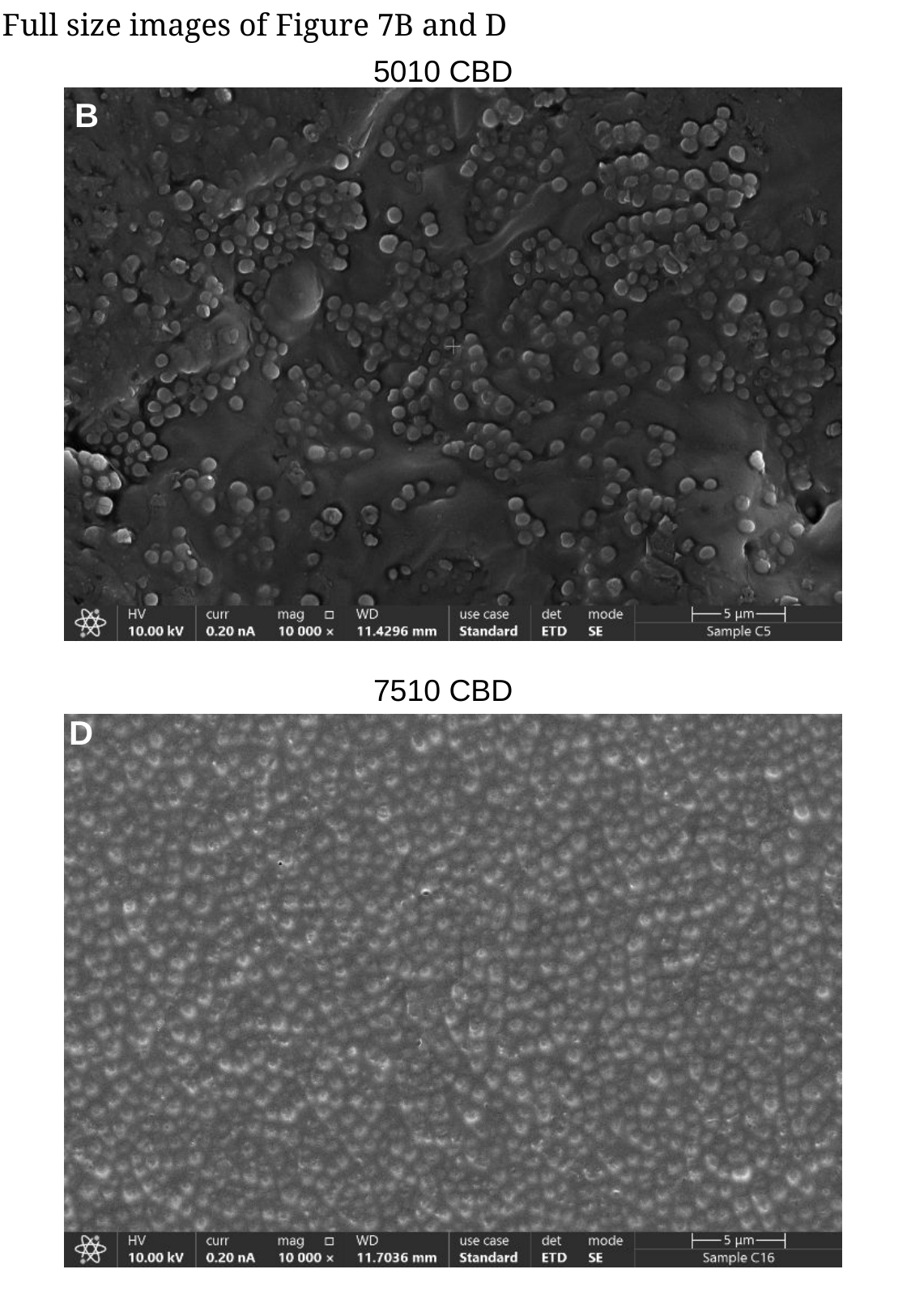

Full size images of Figure 7B and D
5010 CBD
B
7510 CBD
D

## Slide 15
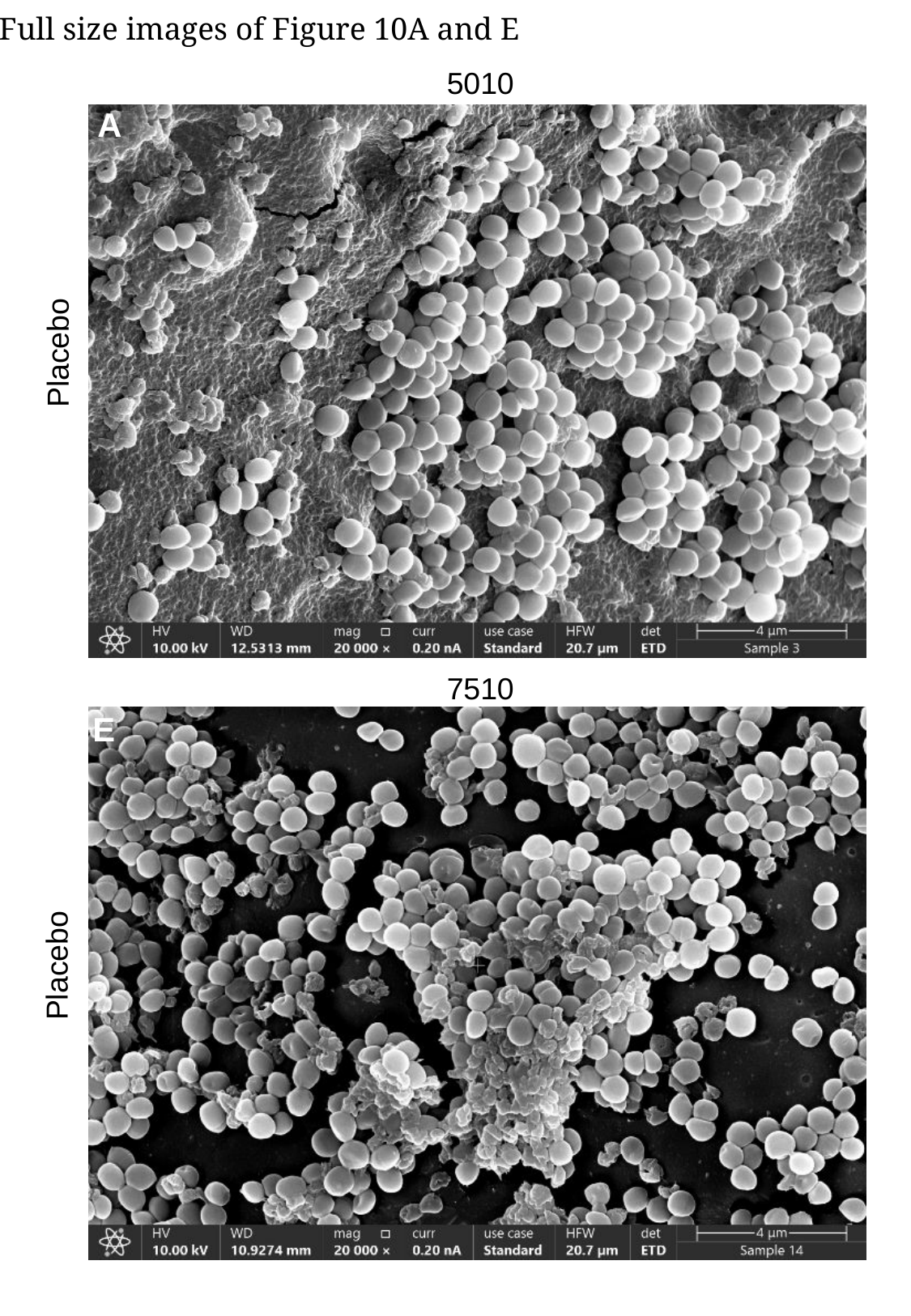

Full size images of Figure 10A and E
5010
A
Placebo
7510
E
Placebo

## Slide 16
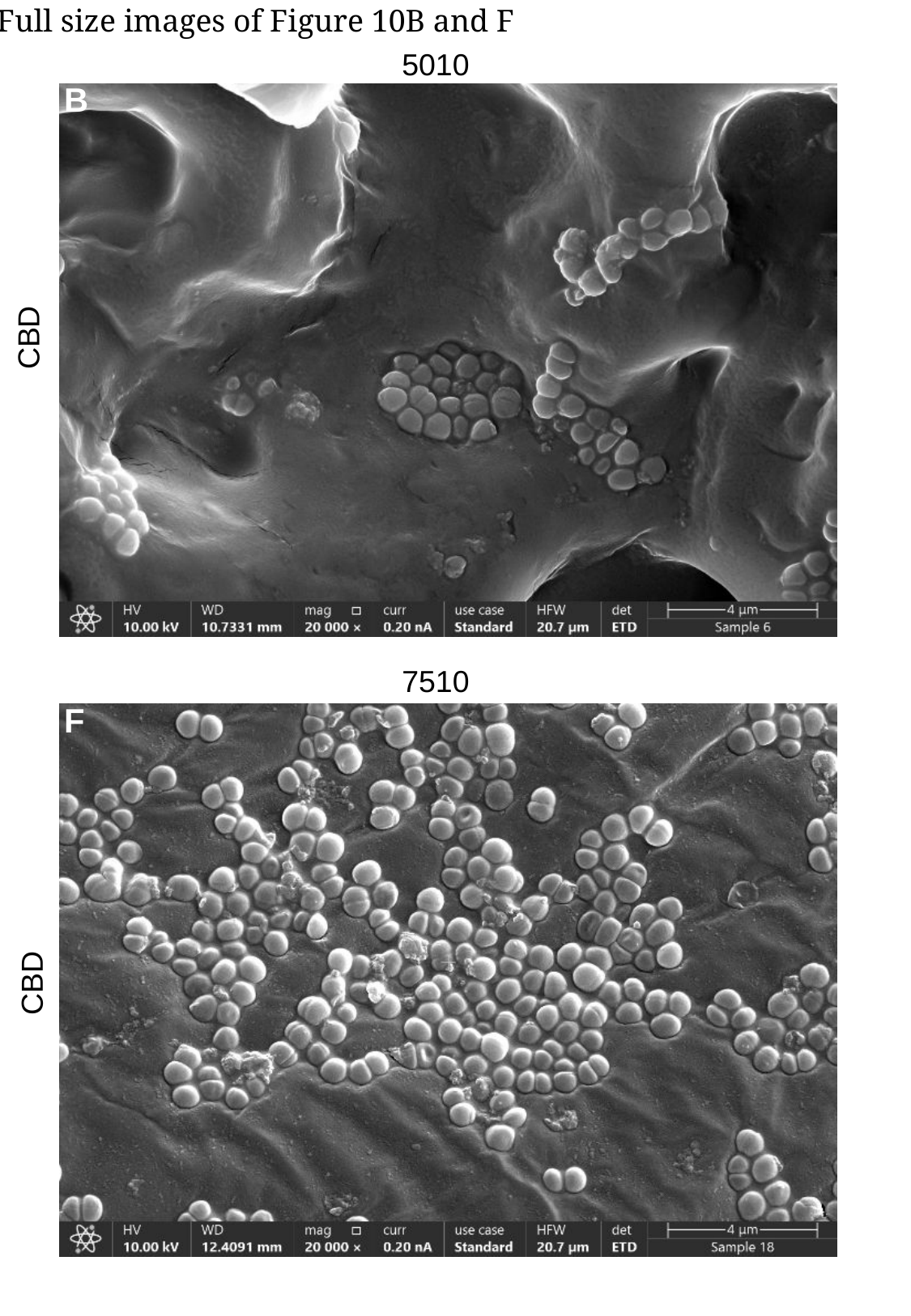

Full size images of Figure 10B and F
5010
B
CBD
7510
F
CBD

## Slide 17
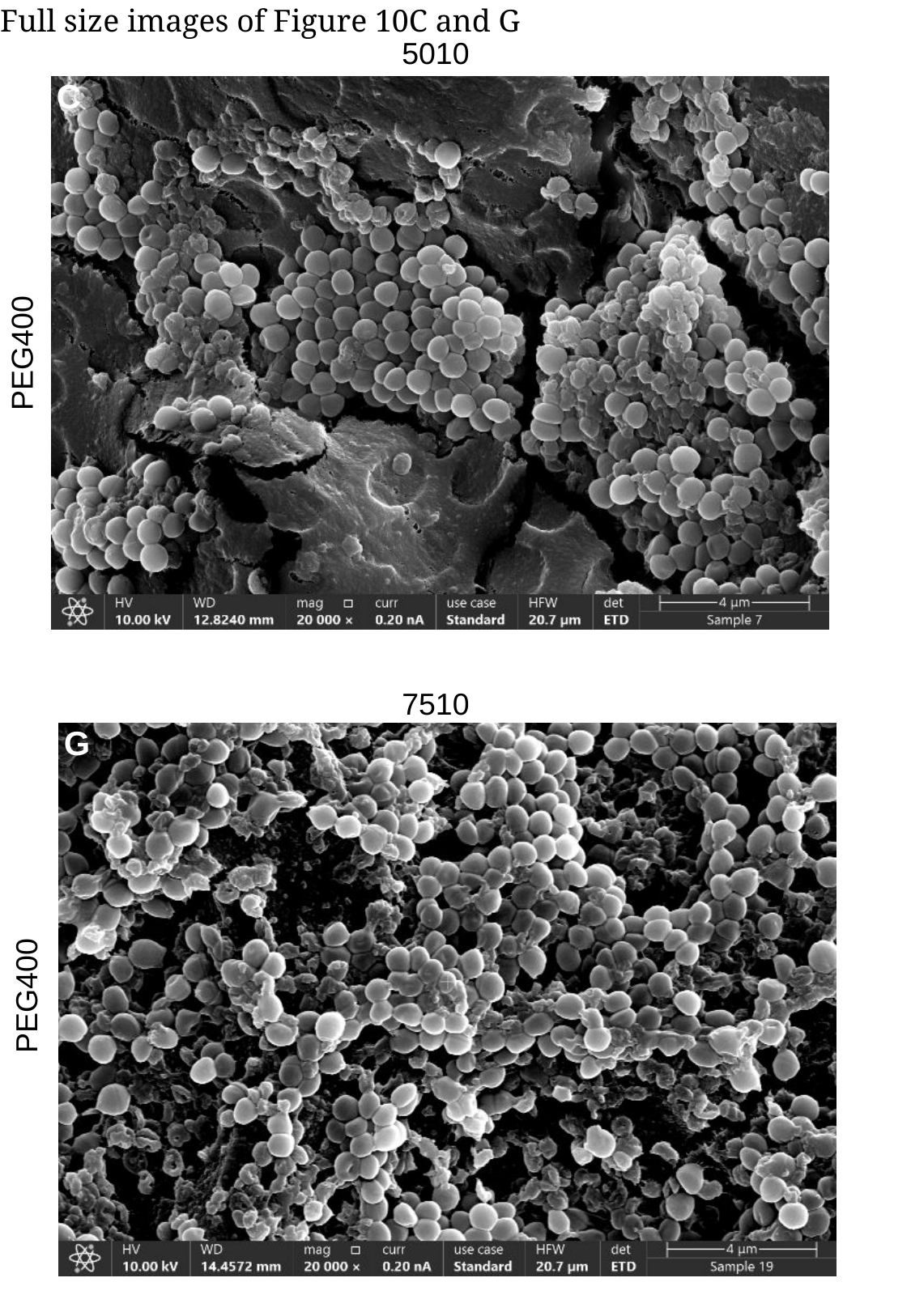

Full size images of Figure 10C and G
5010
C
PEG400
7510
G
PEG400

## Slide 18
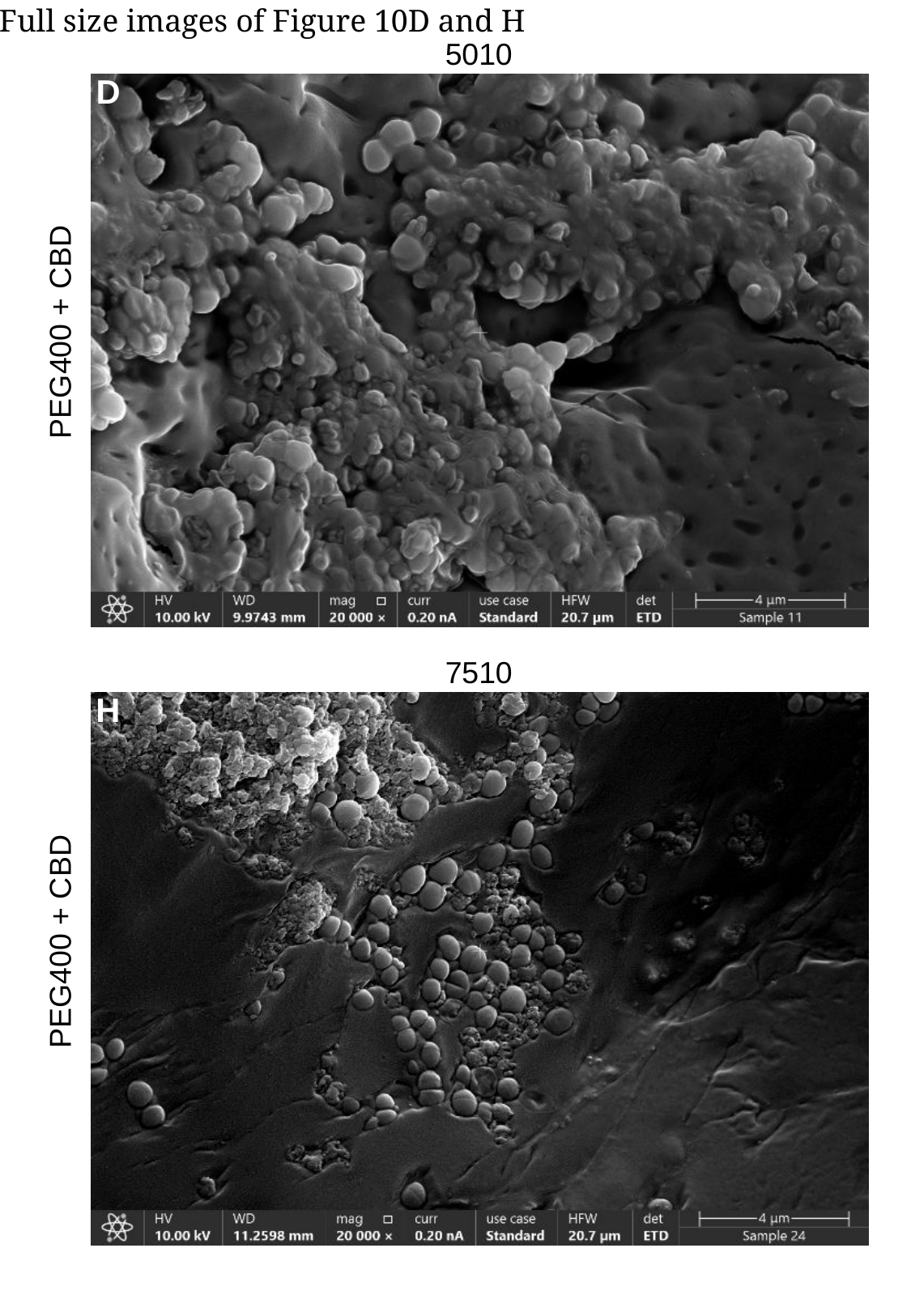

Full size images of Figure 10D and H
5010
D
PEG400 + CBD
7510
H
PEG400 + CBD

## Slide 19
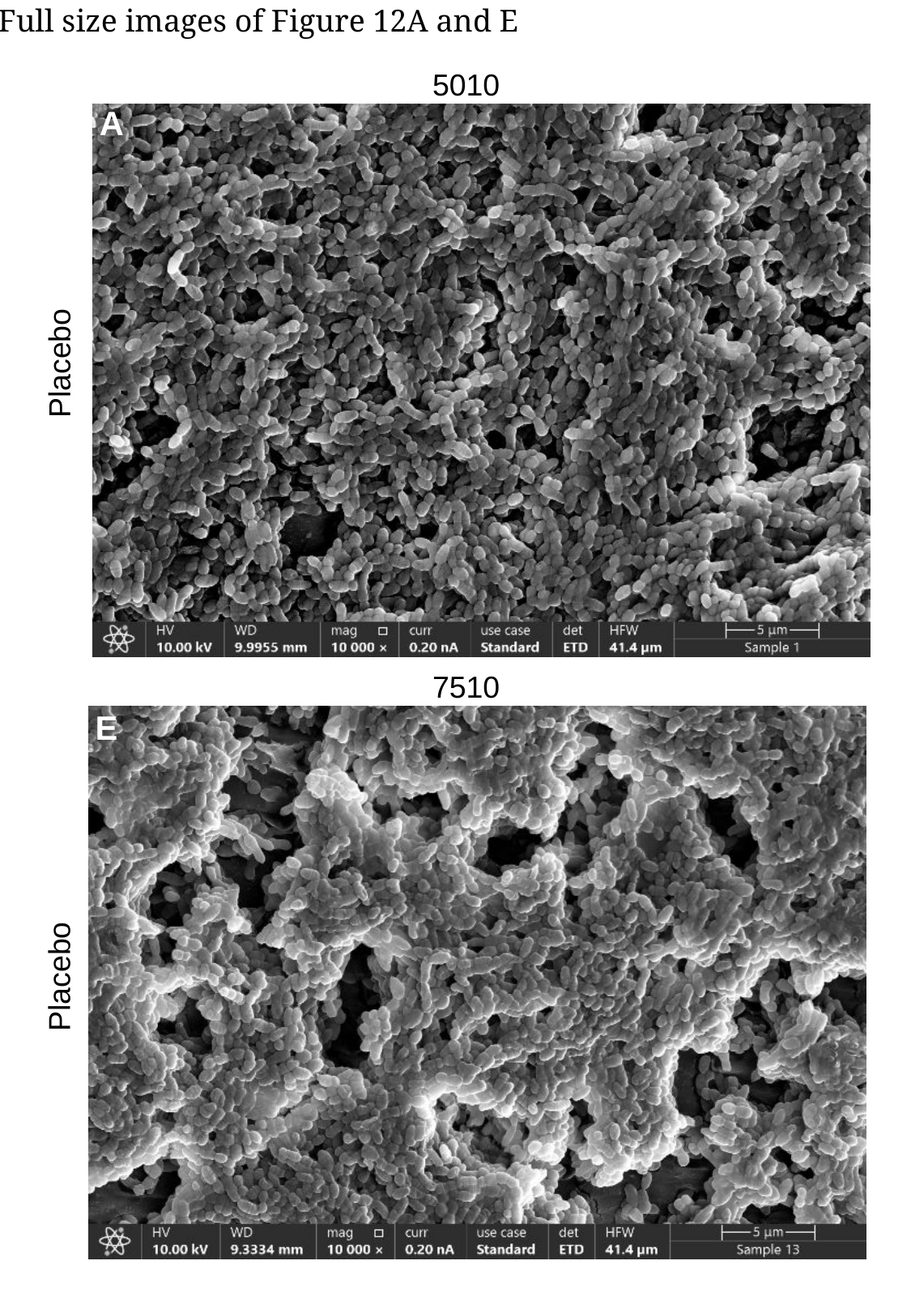

Full size images of Figure 12A and E
5010
A
Placebo
7510
E
Placebo

## Slide 20
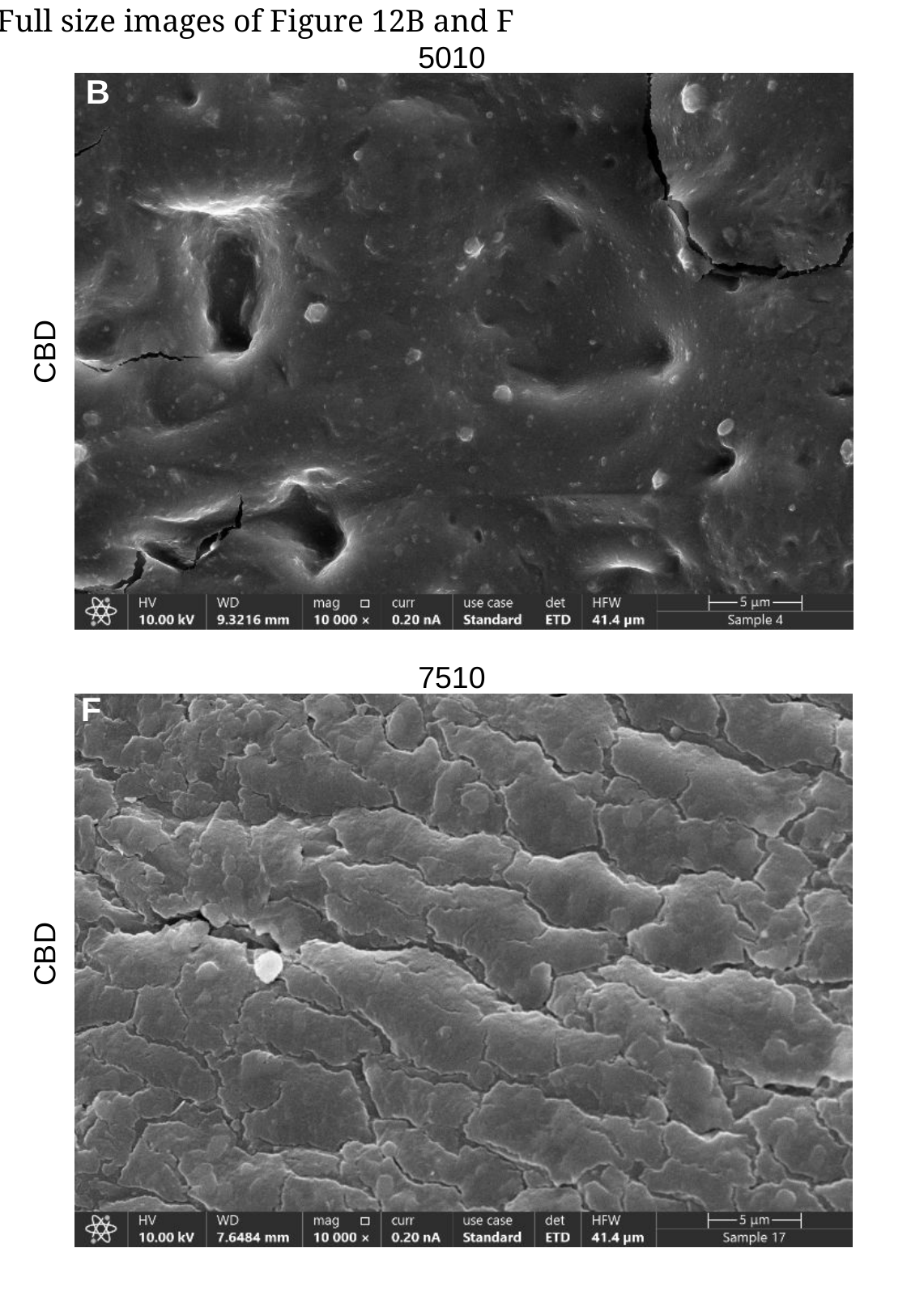

Full size images of Figure 12B and F
5010
B
CBD
7510
F
CBD

## Slide 21
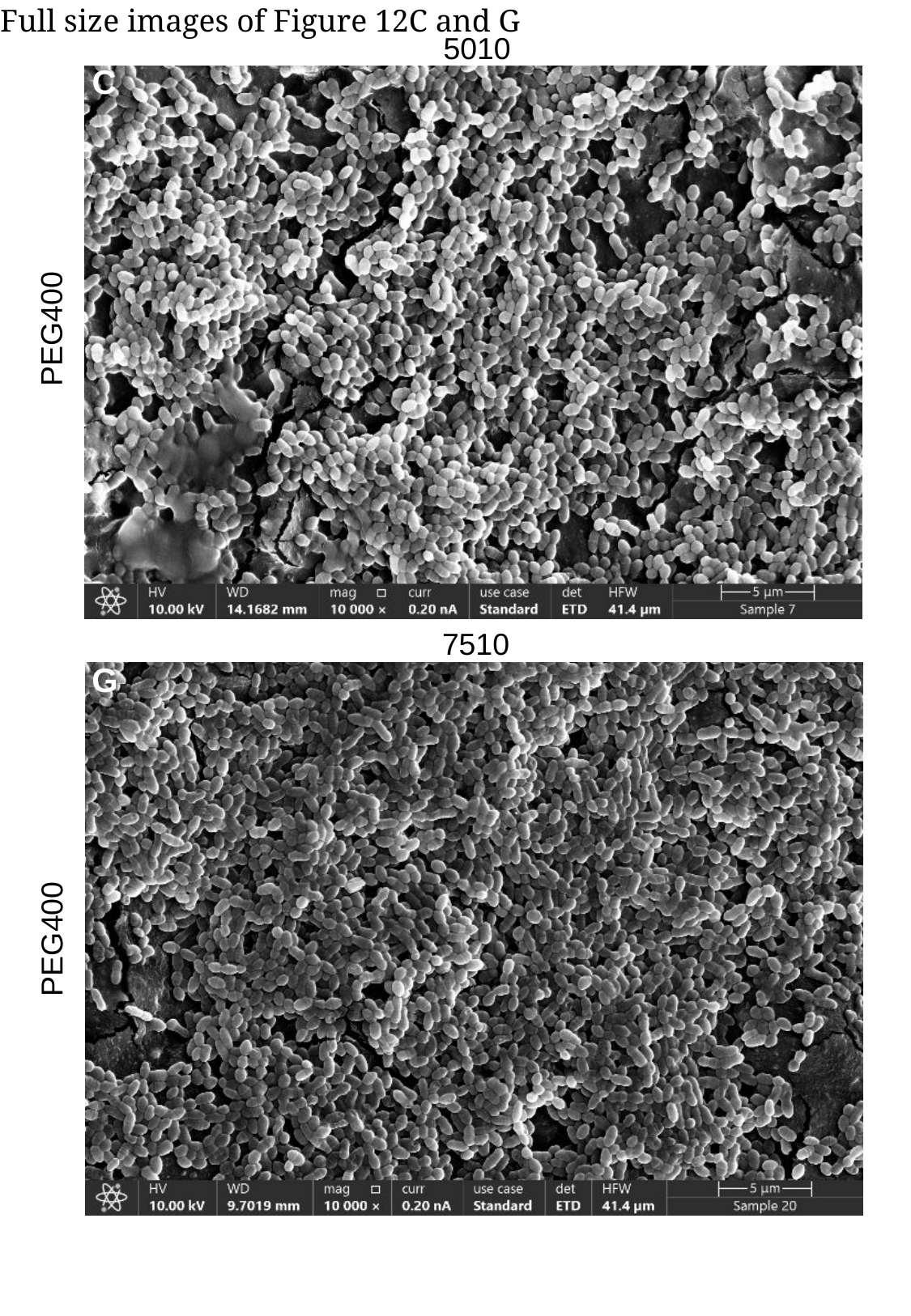

Full size images of Figure 12C and G
5010
C
PEG400
7510
G
PEG400

## Slide 22
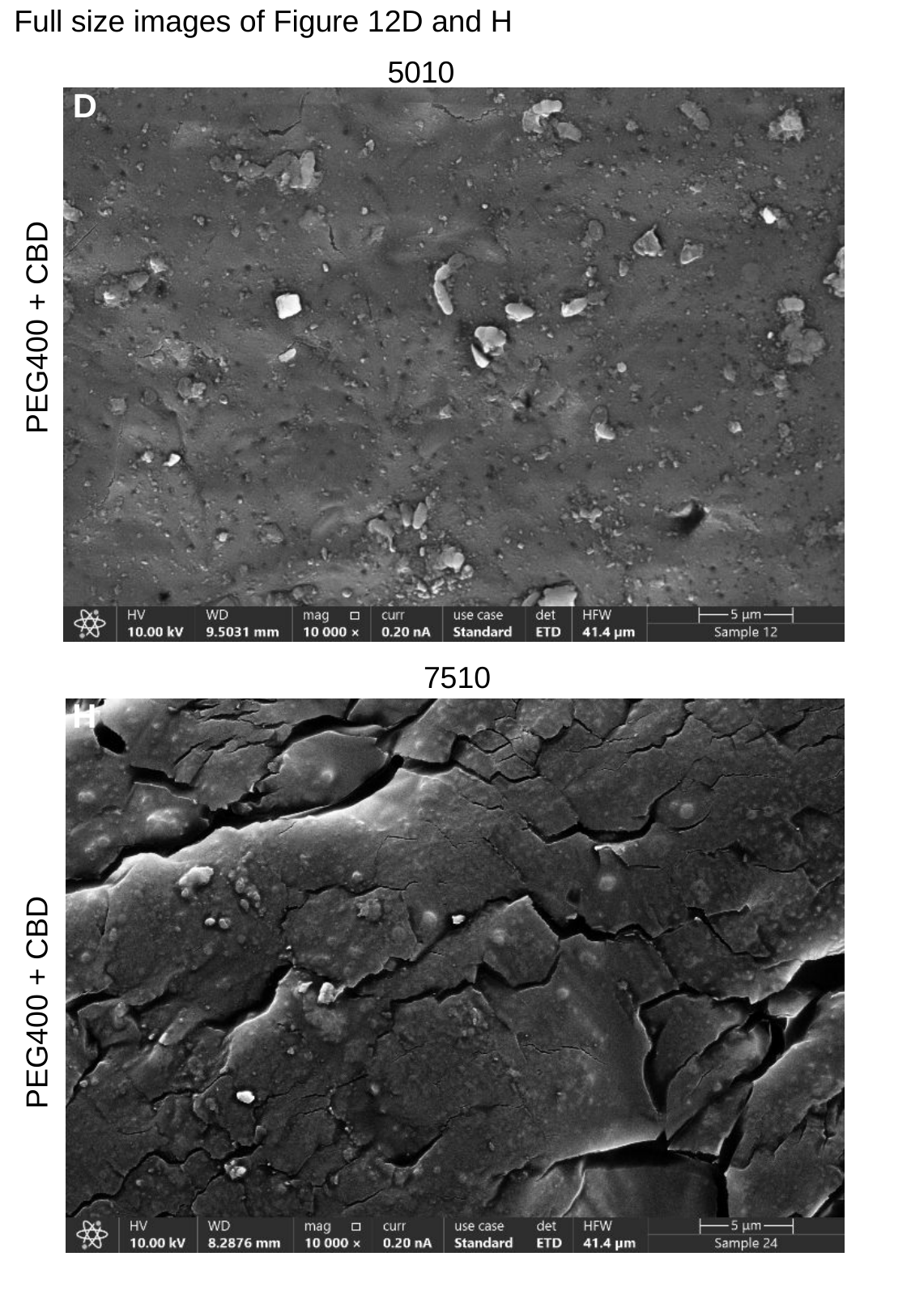

Full size images of Figure 12D and H
5010
D
PEG400 + CBD
7510
H
PEG400 + CBD
