# Supplementary material for: The Incorporation of CBD into Biodegradable DL-Lactide/Glycolide Copolymers Creates a Persistent Antibacterial Environment: An In Vitro Study on Streptococcus mutans and Staphylococcus aureus
Source: Pharmaceutics. 2025 Apr 2;17(4):463. doi: 10.3390/pharmaceutics17040463 (PMC12030335; doi:10.3390/pharmaceutics17040463)

Quantitative Analysis Sample Report

**Batch Data Path File Name**	D:\MassHunter\Data\2022\2022_fitocannabinoids _calib\QuantResults\04_02_2025_CBD_calib.batch.bin		
**Analysis Time Stamp**	3/25/2025 3:57:01 PM	**Analyst Name**	DESKTOP-9792RPL\GCMS
**Report Generation Time**	3/25/2025 3:57:34 PM	**Report Generator Name**	DESKTOP-9792RPL\GCMS
**Calibration Last Update**	3/25/2025 3:57:01 PM	**Batch State**	Processed
**Analyze Quant Version**	10.2	**Report Quant Version**	10.2
**Acq. Date-Time**	2/3/2025 4:27:24 PM	**Data File**	3_2_25_cbd_1_6_uM.D
**Type**	Cal	**Name**	3_2_25_cbd_1_6_uM
**Dil.**	1	**Acq. Method File**	CannabinoidsAgilent 29_05

Sample Chromatogram

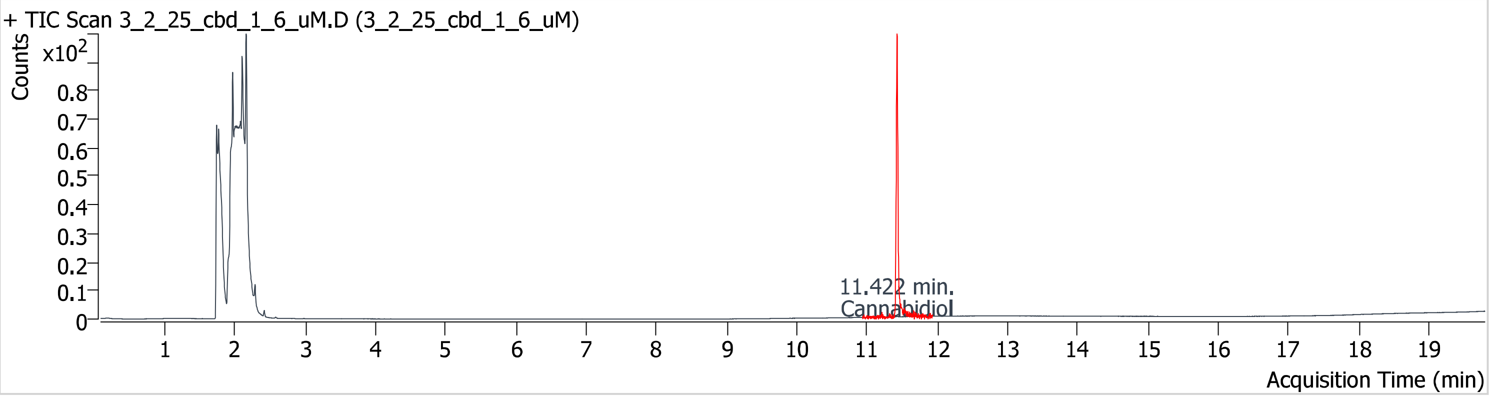

Name	RT	Transition	Resp.	Height	Final Conc.	Units
Cannabidiol	11.422	231.0	19670	11407.69	2.4150	uM

Cannabidiol

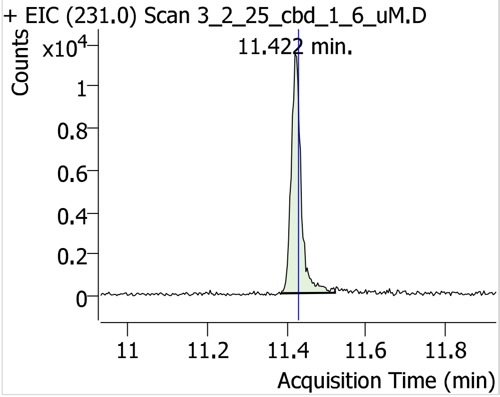

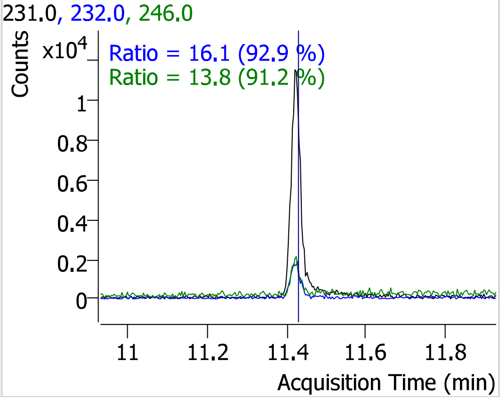

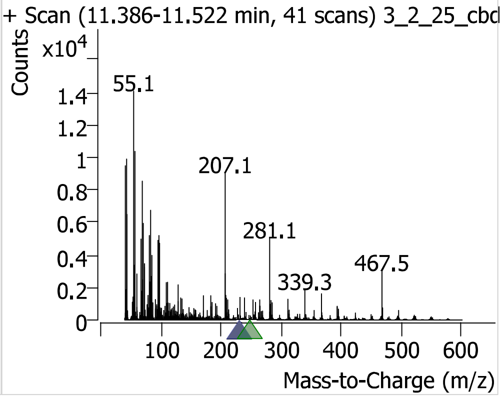

Quantitative Analysis Sample Report

**Batch Data Path File Name**	D:\MassHunter\Data\2022\2022_fitocannabinoids _calib\QuantResults\04_02_2025_CBD_calib.batch.bin		
**Analysis Time Stamp**	3/25/2025 3:57:01 PM	**Analyst Name**	DESKTOP-9792RPL\GCMS
**Report Generation Time**	3/25/2025 3:57:34 PM	**Report Generator Name**	DESKTOP-9792RPL\GCMS
**Calibration Last Update**	3/25/2025 3:57:01 PM	**Batch State**	Processed
**Analyze Quant Version**	10.2	**Report Quant Version**	10.2

**Acq. Date-Time**	2/3/2025 4:51:44 PM	**Data File**	3_2_25_cbd_3_2_uM.D
**Type**	Cal	**Name**	3_2_25_cbd_3_2_uM
**Dil.**	1	**Acq. Method File**	CannabinoidsAgilent 29_05

Sample Chromatogram

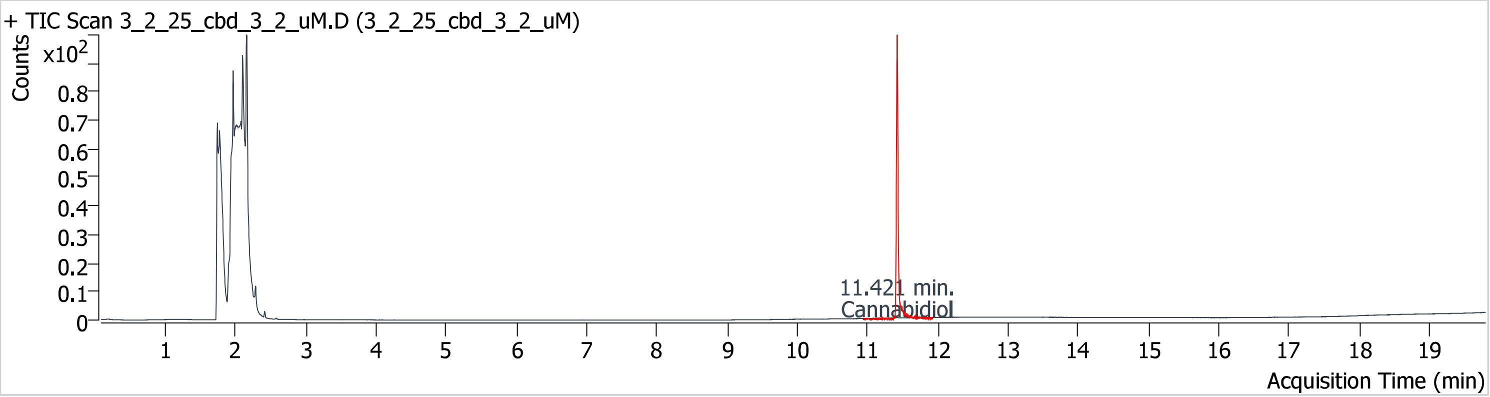

Name	RT	Transition	Resp.	Height	Final Conc.	Units
Cannabidiol	11.421	231.0	37312	22161.73	3.8068	uM

Cannabidiol

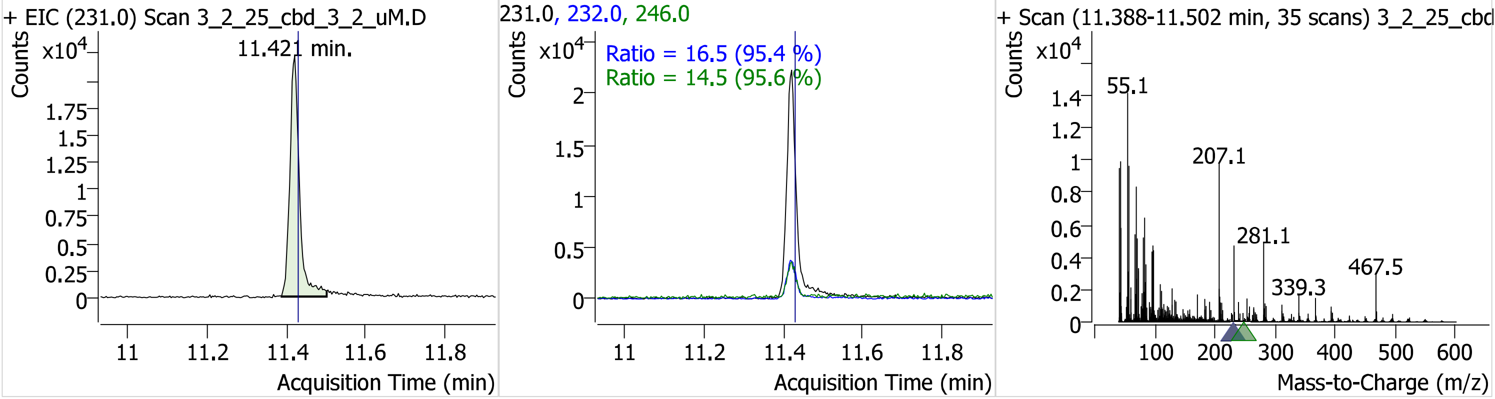

Quantitative Analysis Sample Report

**Batch Data Path File Name**	D:\MassHunter\Data\2022\2022_fitocannabinoids _calib\QuantResults\04_02_2025_CBD_calib.batch.bin		
**Analysis Time Stamp**	3/25/2025 3:57:01 PM	**Analyst Name**	DESKTOP-9792RPL\GCMS
**Report Generation Time**	3/25/2025 3:57:34 PM	**Report Generator Name**	DESKTOP-9792RPL\GCMS
**Calibration Last Update**	3/25/2025 3:57:01 PM	**Batch State**	Processed
**Analyze Quant Version**	10.2	**Report Quant Version**	10.2
**Acq. Date-Time**	2/3/2025 5:16:00 PM	**Data File**	3_2_25_cbd_32_uM.D
**Type**	Cal	**Name**	3_2_25_cbd_32_uM
**Dil.**	1	**Acq. Method File**	CannabinoidsAgilent 29_05

Sample Chromatogram

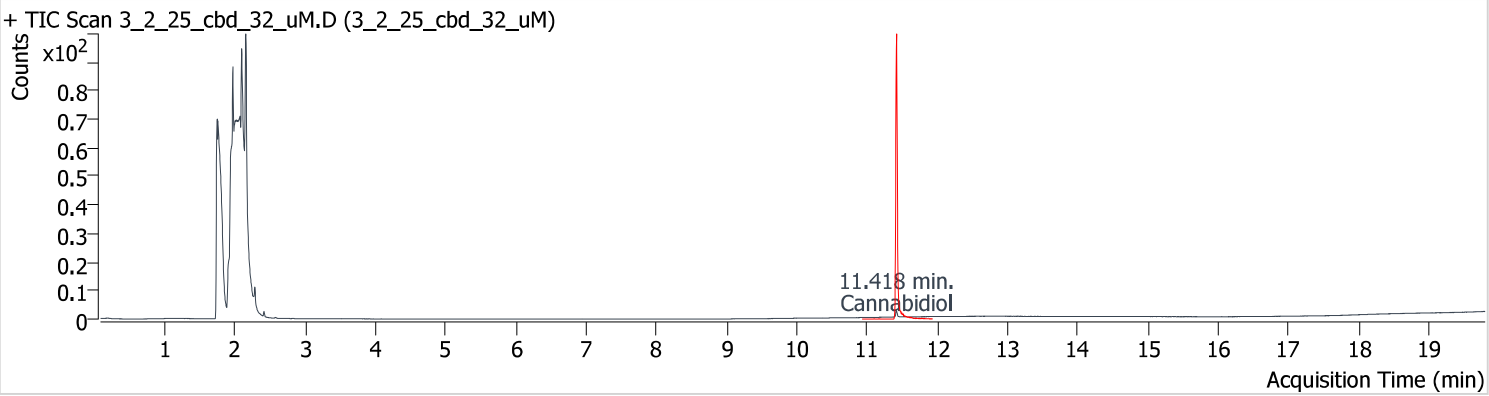

Name	RT	Transition	Resp.	Height	Final Conc.	Units
Cannabidiol	11.418	231.0	374788	233315.01	30.4314	uM

Cannabidiol

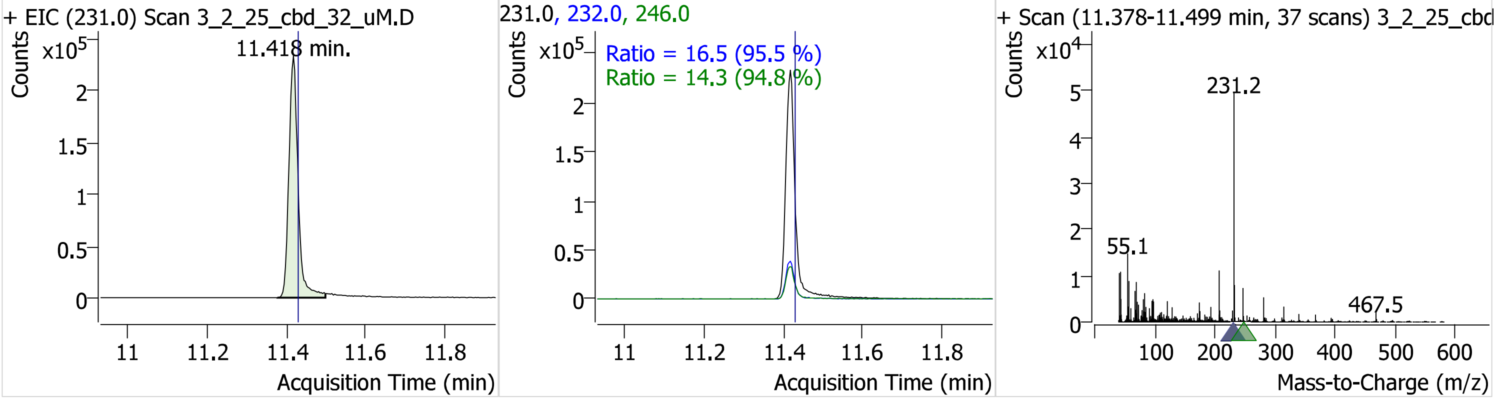

Quantitative Analysis Sample Report

INSTITUTE FOR PERSONALIZED AND TRANSLATIONAL MEDICINE

**Batch Data Path File Name**	D:\MassHunter\Data\2022\2022_fitocannabinoids _calib\QuantResults\04_02_2025_CBD_calib.batch.bin		
**Analysis Time Stamp**	3/25/2025 3:57:01 PM	**Analyst Name**	DESKTOP-9792RPL\GCMS
**Report Generation Time**	3/25/2025 3:57:34 PM	**Report Generator Name**	DESKTOP-9792RPL\GCMS
**Calibration Last Update**	3/25/2025 3:57:01 PM	**Batch State**	Processed
**Analyze Quant Version**	10.2	**Report Quant Version**	10.2
**Acq. Date-Time**	2/3/2025 5:40:23 PM	**Data File**	3_2_25_cbd_320_uM.D
**Type**	Cal	**Name**	3_2_25_cbd_320_uM
**Dil.**	1	**Acq. Method File**	CannabinoidsAgilent 29_05

Sample Chromatogram

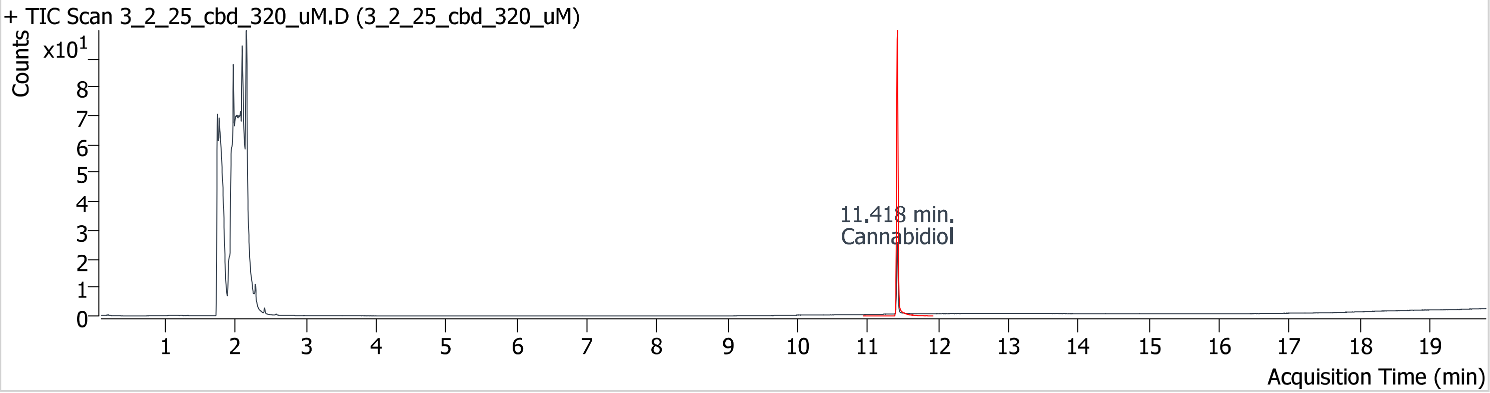

Name	RT	Transition	Resp.	Height	Final Conc.	Units
Cannabidiol	11.418	231.0	4047035	2654838.60	320.1467	uM

Cannabidiol

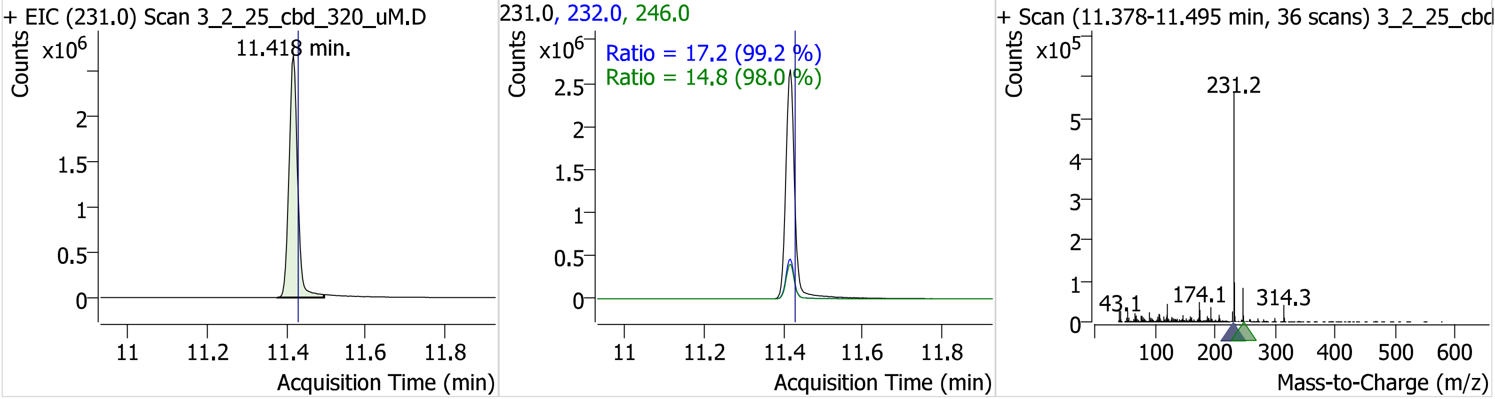

**Batch Data Path File Name**	D:\MassHunter\Data\2022\2022_fitocannabinoids_calib\QuantResults\04_02_2025_CBD_calib.batch.bin		
**Analysis Time Stamp**	3/25/2025 3:57:01 PM	**Analyst Name**	DESKTOP-9792RPL\GCMS
**Report Generation Time**	3/25/2025 3:57:34 PM	**Report Generator Name**	DESKTOP-9792RPL\GCMS
**Calibration Last Update**	3/25/2025 3:57:01 PM	**Batch State**	Processed
**Analyze Quant Version**	10.2	**Report Quant Version**	10.2
**Acq. Date-Time**	2/10/2025 7:48:51 PM	**Data File**	10_2_25_DAY3_5010_PLACEBO_1.D
**Type**	Sample	**Name**	10_2_25_DAY3_5010_PLACEBO_1
**Dil.**	1	**Acq. Method File**	CannabinoidsAgilent 29_05

Sample Chromatogram

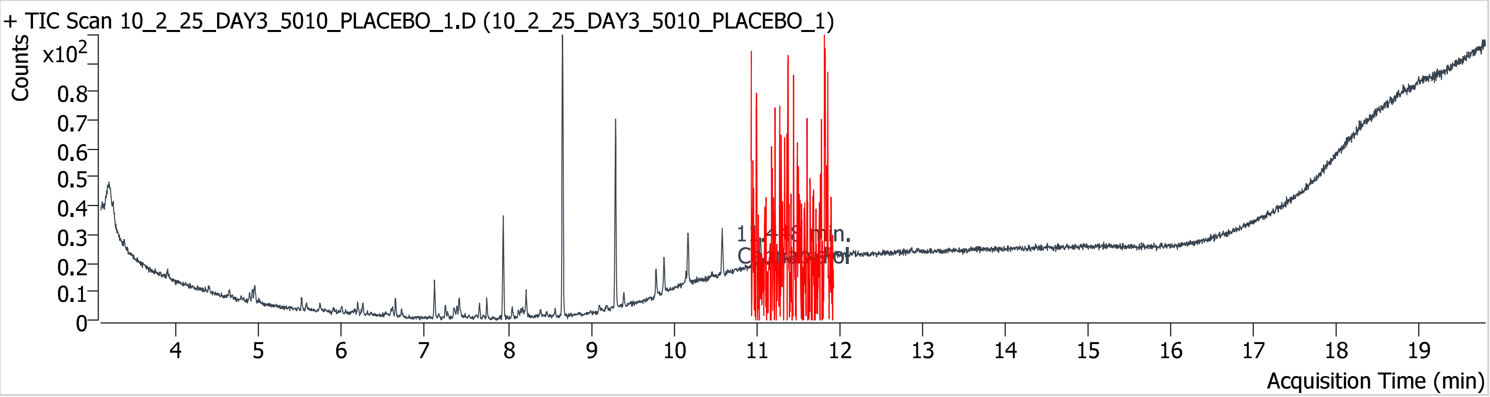

Name	RT	Transition	Resp.	Height	Final Conc.	Units
Cannabidiol	11.448	231.0	28	89.20	0.8654	uM

Cannabidiol

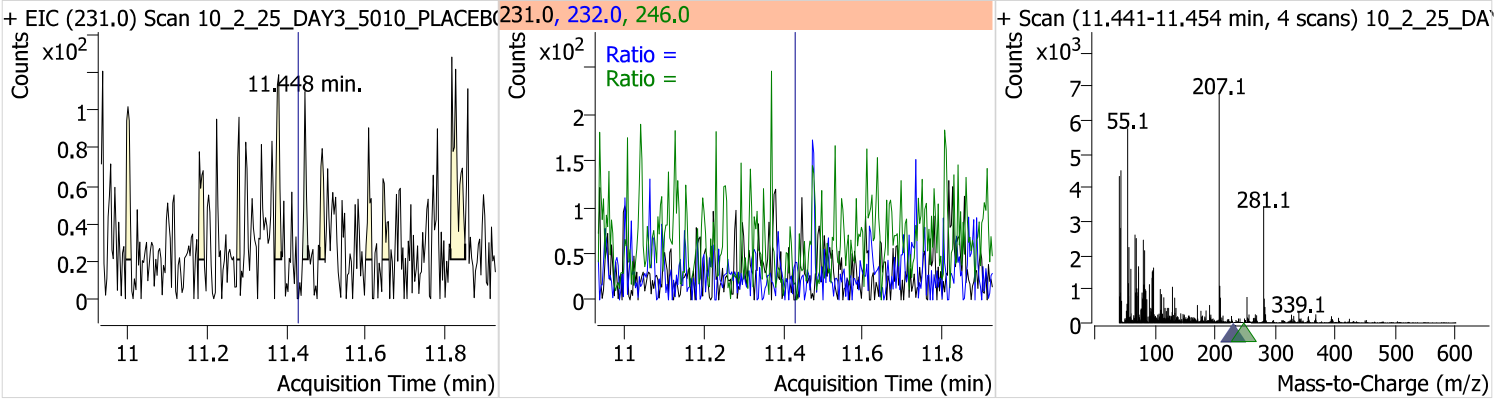

Quantitative Analysis Sample Report

**Batch Data Path File Name**	D:\MassHunter\Data\2022\2022_fitocannabinoids _calib\QuantResults\04_02_2025_CBD_calib.batch.bin		
**Analysis Time Stamp**	3/25/2025 3:57:01 PM	**Analyst Name**	DESKTOP-9792RPL\GCMS
**Report Generation Time**	3/25/2025 3:57:34 PM	**Report Generator Name**	DESKTOP-9792RPL\GCMS
**Calibration Last Update**	3/25/2025 3:57:01 PM	**Batch State**	Processed
**Analyze Quant Version**	10.2	**Report Quant Version**	10.2
**Acq. Date-Time**	2/10/2025 9:01:49 PM	**Data File**	10_2_25_DAY3_5010_PLACEBO_PEG400_1.D
**Type**	Sample	**Name**	10_2_25_DAY3_5010_PLACEBO_PEG400_1
**Dil.**	1	**Acq. Method File**	CannabinoidsAgilent 29_05

Sample Chromatogram

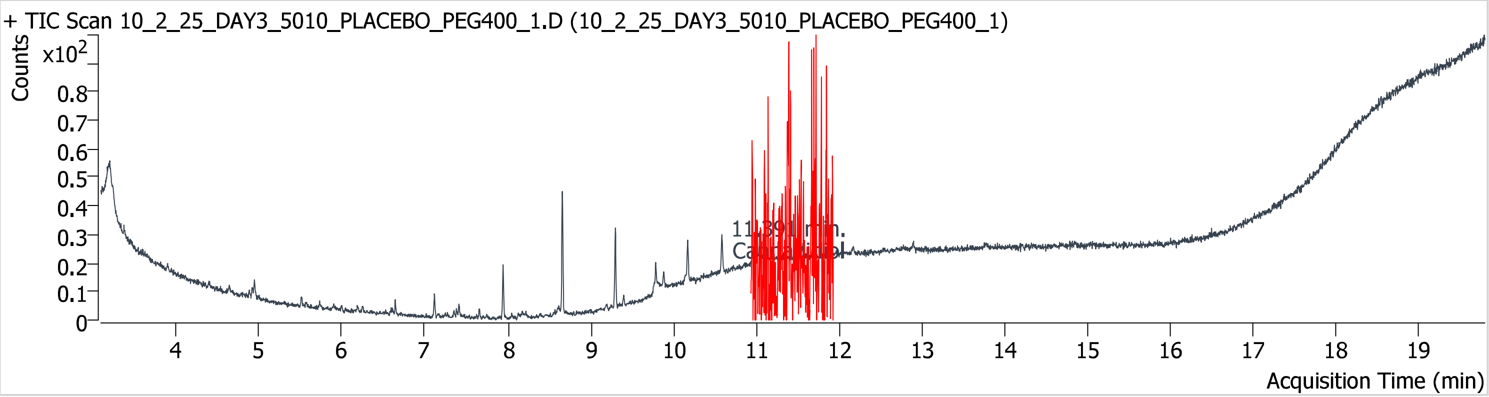

Name	RT	Transition	Resp.	Height	Final Conc.	Units
Cannabidiol	11.391	231.0	67	91.80	0.8685	uM

Cannabidiol

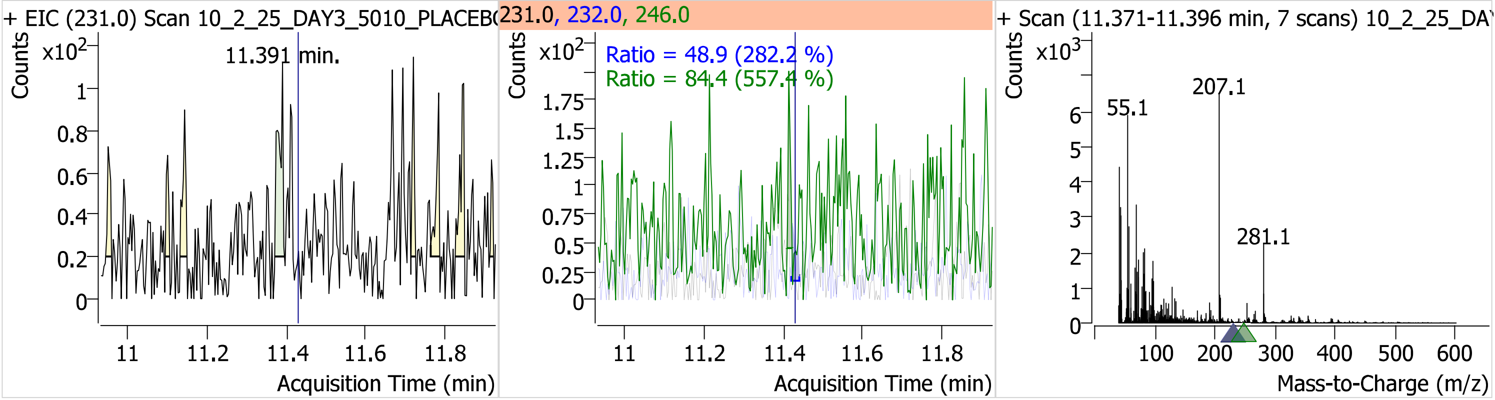

Quantitative Analysis Sample Report

**Batch Data Path File Name**	D:\MassHunter\Data\2022\2022_fitocannabinoids _calib\QuantResults\04_02_2025_CBD_calib.batch.bin		
**Analysis Time Stamp**	3/25/2025 3:57:01 PM	**Analyst Name**	DESKTOP-9792RPL\GCMS
**Report Generation Time**	3/25/2025 3:57:34 PM	**Report Generator Name**	DESKTOP-9792RPL\GCMS
**Calibration Last Update**	3/25/2025 3:57:01 PM	**Batch State**	Processed
**Analyze Quant Version**	10.2	**Report Quant Version**	10.2
**Acq. Date-Time**	2/10/2025 10:14:34 PM	**Data File**	10_2_25_DAY3_5010_CBD_1.D
**Type**	Sample	**Name**	10_2_25_DAY3_5010_CBD_1
**Dil.**	1	**Acq. Method File**	CannabinoidsAgilent 29_05

Sample Chromatogram

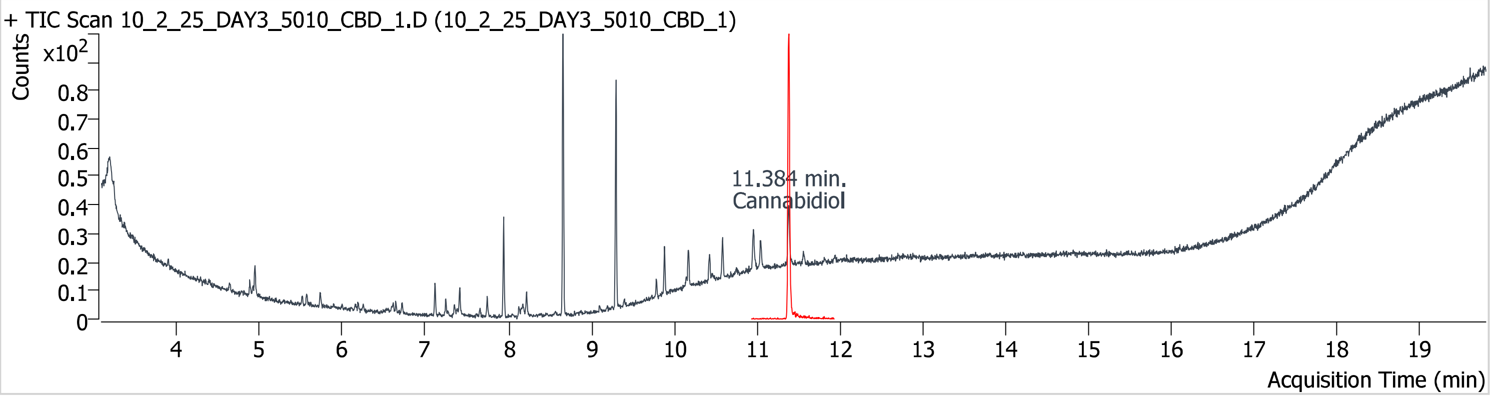

Name	RT	Transition	Resp.	Height	Final Conc.	Units
Cannabidiol	11.384	231.0	39714	24826.40	3.9964	uM

Cannabidiol

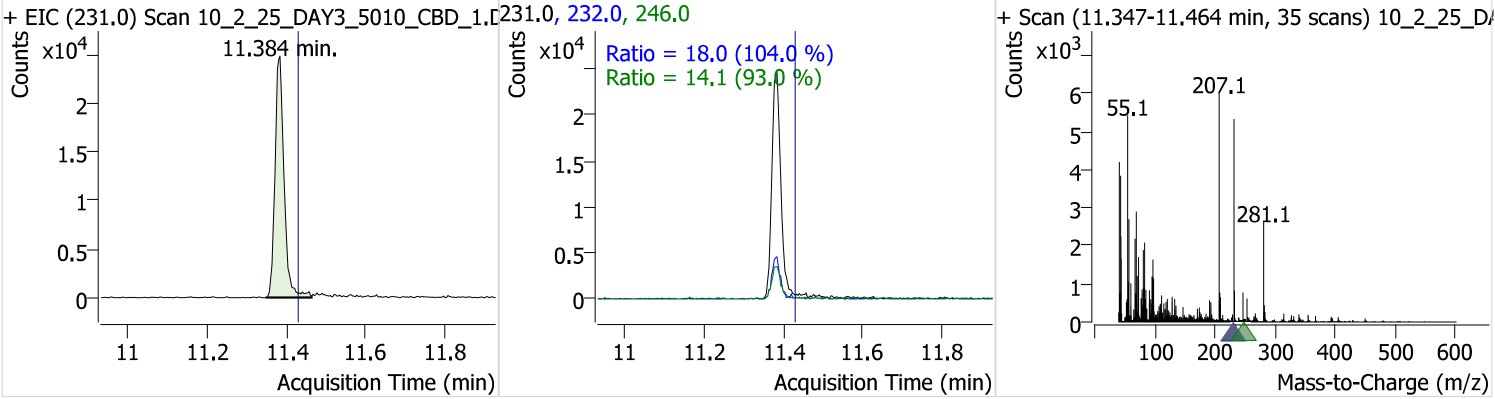

Quantitative Analysis Sample Report

**Batch Data Path File Name**	D:\MassHunter\Data\2022\2022_fitocannabinoids _calib\QuantResults\04_02_2025_CBD_calib.batch.bin		
**Analysis Time Stamp**	3/25/2025 3:57:01 PM	**Analyst Name**	DESKTOP-9792RPL\GCMS
**Report Generation Time**	3/25/2025 3:57:34 PM	**Report Generator Name**	DESKTOP-9792RPL\GCMS
**Calibration Last Update**	3/25/2025 3:57:01 PM	**Batch State**	Processed
**Analyze Quant Version**	10.2	**Report Quant Version**	10.2
**Acq. Date-Time**	2/10/2025 11:27:24 PM	**Data File**	10_2_25_DAY3_5010_CBD_PEG400_1.D
**Type**	Sample	**Name**	10_2_25_DAY3_5010_CBD_PEG400_1
**Dil.**	1	**Acq. Method File**	CannabinoidsAgilent 29_05

Sample Chromatogram

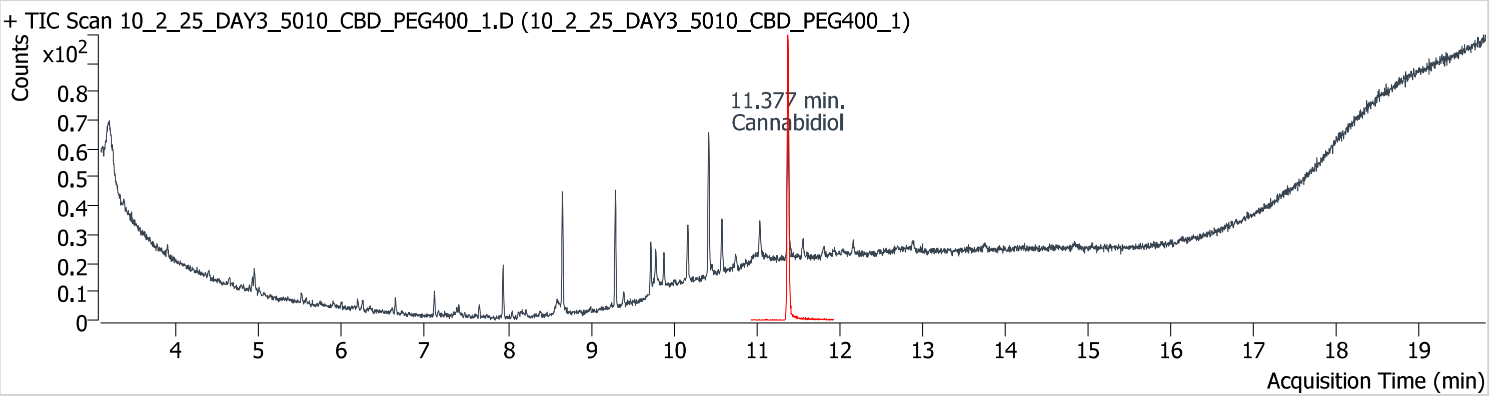

Name	RT	Transition	Resp.	Height	Final Conc.	Units
Cannabidiol	11.377	231.0	70770	45879.38	6.4465	uM

Cannabidiol

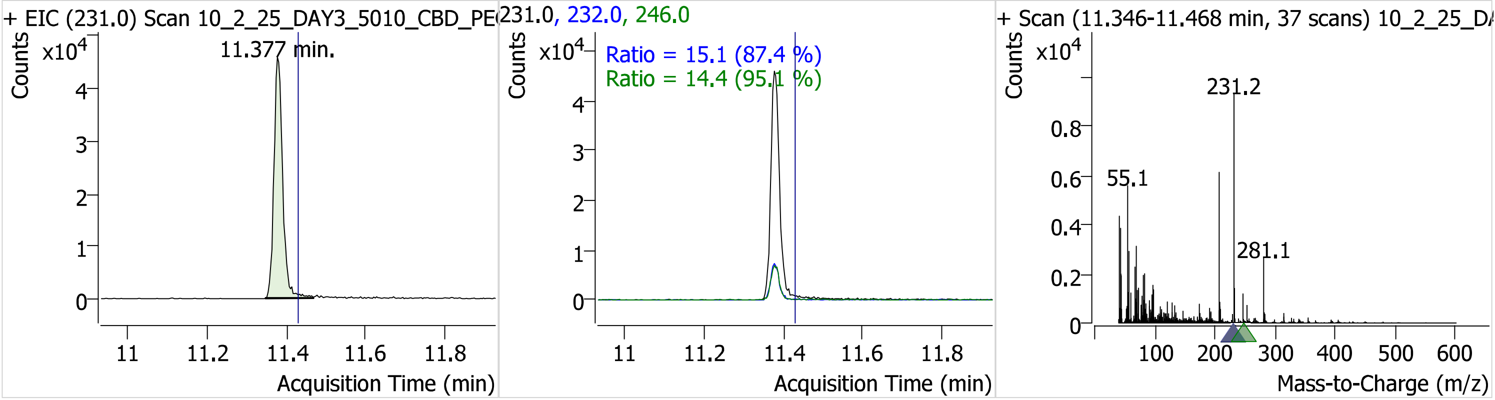

Quantitative Analysis Sample Report

**Batch Data Path File Name**	D:\MassHunter\Data\2022\2022_fitocannabinoids_calib\QuantResults\04_02_2025_CBD_calib.batch.bin		
**Analysis Time Stamp**	3/25/2025 3:57:01 PM	**Analyst Name**	DESKTOP-9792RPL\GCMS
**Report Generation Time**	3/25/2025 3:57:34 PM	**Report Generator Name**	DESKTOP-9792RPL\GCMS
**Calibration Last Update**	3/25/2025 3:57:01 PM	**Batch State**	Processed
**Analyze Quant Version**	10.2	**Report Quant Version**	10.2
**Acq. Date-Time**	2/11/2025 3:52:48 PM	**Data File**	11_2_25_DAY3_7510_PLACEBO_1.D
**Type**	Sample	**Name**	11_2_25_DAY3_7510_PLACEBO_1
**Dil.**	1	**Acq. Method File**	CannabinoidsAgilent 29_05

Sample Chromatogram

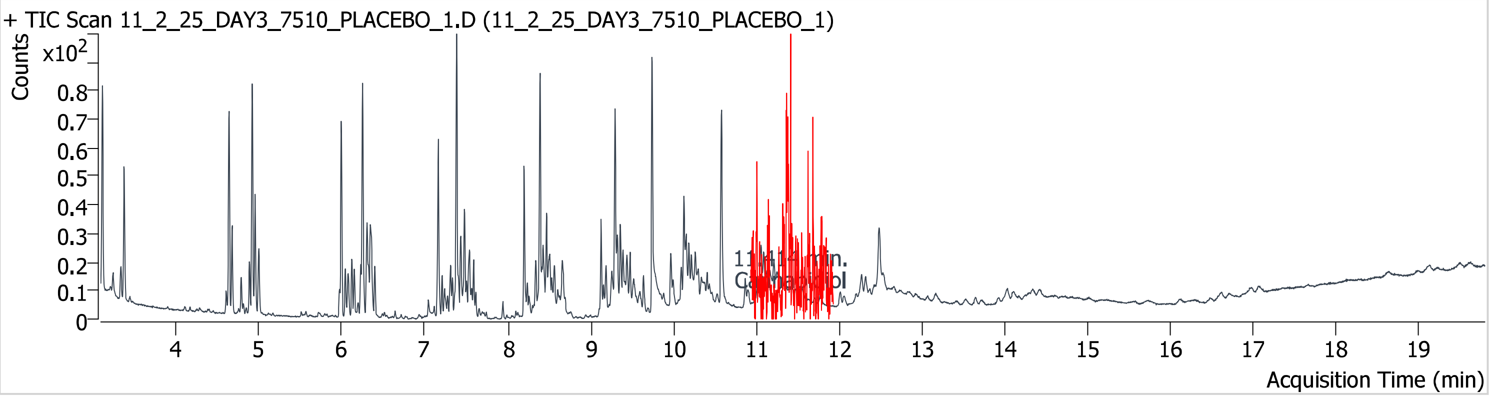

Name	RT	Transition	Resp.	Height	Final Conc.	Units
Cannabidiol	11.414	231.0	78	225.52	0.8694	uM

Cannabidiol

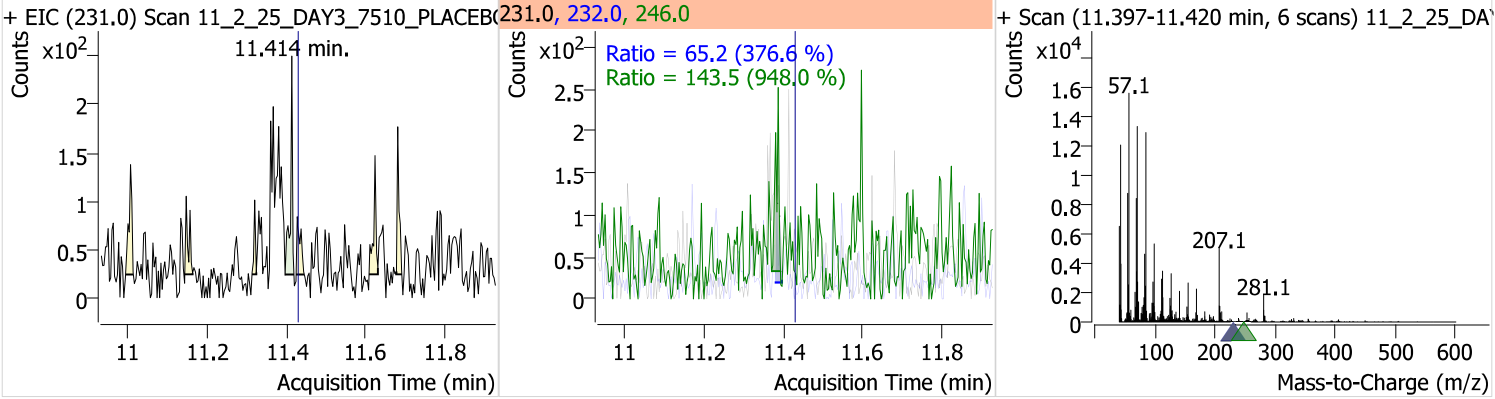

Quantitative Analysis Sample Report

**Batch Data Path File Name**	D:\MassHunter\Data\2022\2022_fitocannabinoids _calib\QuantResults\04_02_2025_CBD_calib.batch.bin		
**Analysis Time Stamp**	3/25/2025 3:57:01 PM	**Analyst Name**	DESKTOP-9792RPL\GCMS
**Report Generation Time**	3/25/2025 3:57:34 PM	**Report Generator Name**	DESKTOP-9792RPL\GCMS
**Calibration Last Update**	3/25/2025 3:57:01 PM	**Batch State**	Processed
**Analyze Quant Version**	10.2	**Report Quant Version**	10.2
**Acq. Date-Time**	2/11/2025 5:05:37 PM	**Data File**	11_2_25_DAY3_7510_PLACEBO_PEG400_1.D
**Type**	Sample	**Name**	11_2_25_DAY3_7510_PLACEBO_PEG400_1
**Dil.**	1	**Acq. Method File**	CannabinoidsAgilent 29_05

Sample Chromatogram

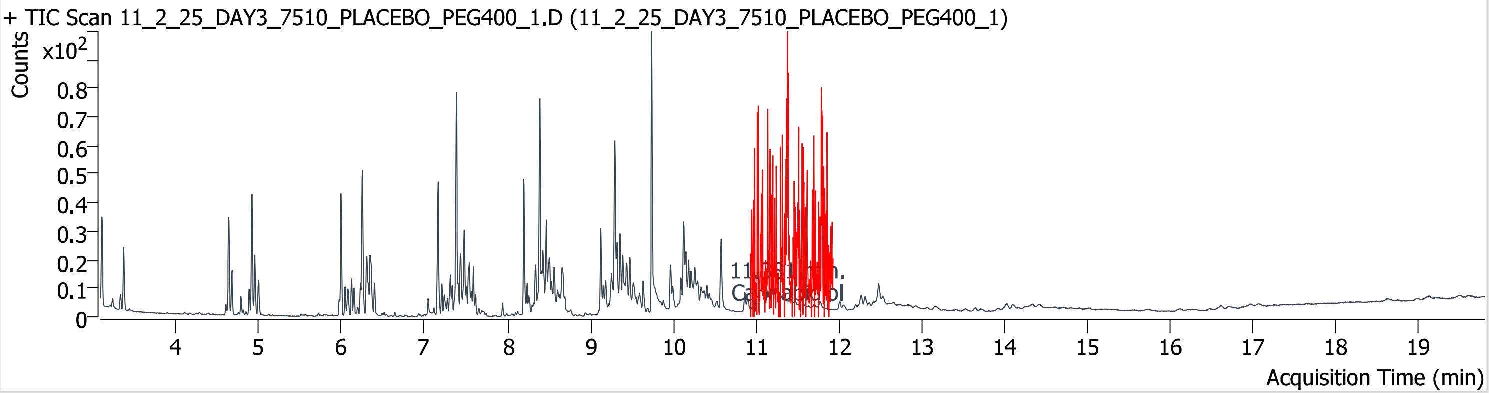

Name	RT	Transition	Resp.	Height	Final Conc.	Units
Cannabidiol	11.381	231.0	272	184.90	0.8847	uM

Cannabidiol

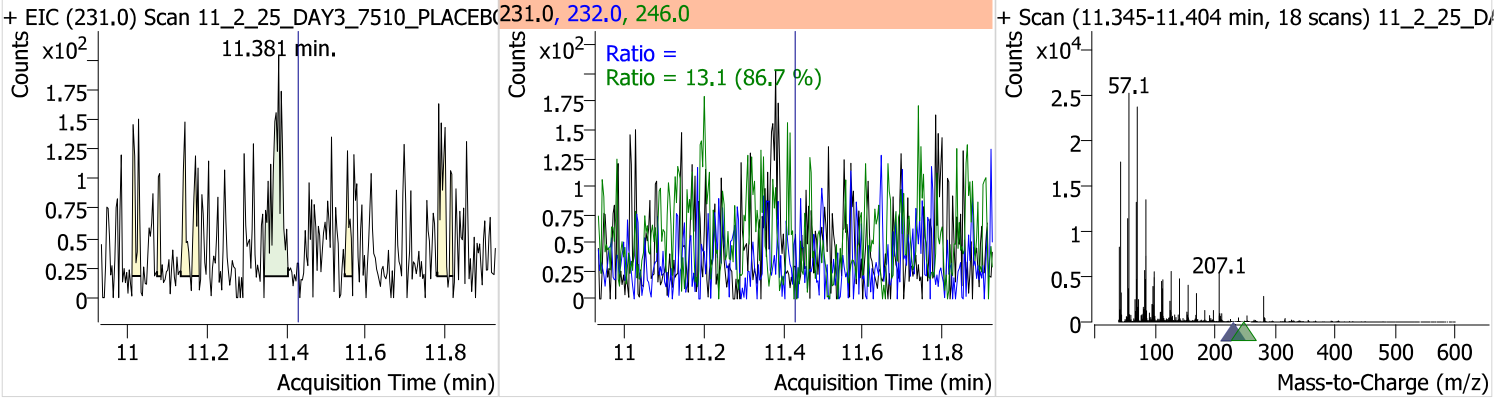

Quantitative Analysis Sample Report

**Batch Data Path File Name**	D:\MassHunter\Data\2022\2022_fitocannabinoids_calib\QuantResults\04_02_2025_CBD_calib.batch.bin		
**Analysis Time Stamp**	3/25/2025 3:57:01 PM	**Analyst Name**	DESKTOP-9792RPL\GCMS
**Report Generation Time**	3/25/2025 3:57:34 PM	**Report Generator Name**	DESKTOP-9792RPL\GCMS
**Calibration Last Update**	3/25/2025 3:57:01 PM	**Batch State**	Processed
**Analyze Quant Version**	10.2	**Report Quant Version**	10.2
**Acq. Date-Time**	2/11/2025 6:18:17 PM	**Data File**	11_2_25_DAY3_7510_CBD_1.D
**Type**	Sample	**Name**	11_2_25_DAY3_7510_CBD_1
**Dil.**	1	**Acq. Method File**	CannabinoidsAgilent 29_05

Sample Chromatogram

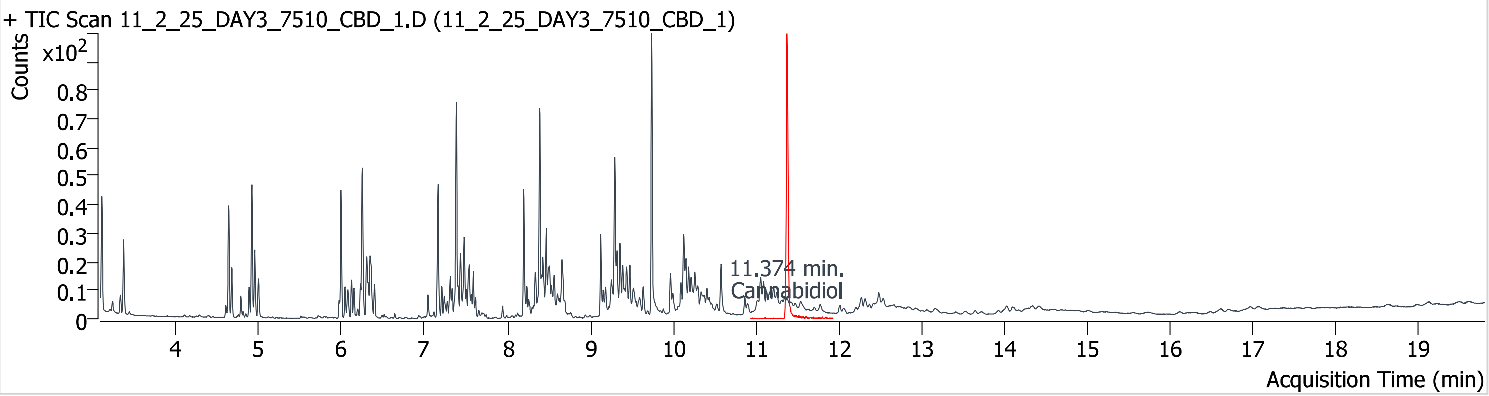

Name	RT	Transition	Resp.	Height	Final Conc.	Units
Cannabidiol	11.374	231.0	55866	36509.95	5.2707	uM

Cannabidiol

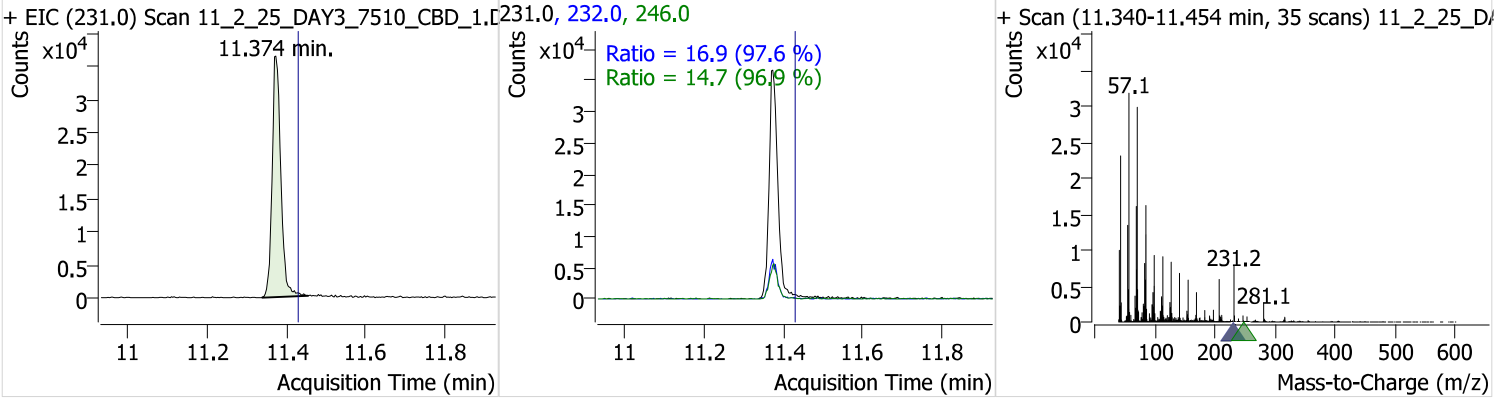

Quantitative Analysis Sample Report

**Batch Data Path File Name**	D:\MassHunter\Data\2022\2022_fitocannabinoids _calib\QuantResults\04_02_2025_CBD_calib.batch.bin		
**Analysis Time Stamp**	3/25/2025 3:57:01 PM	**Analyst Name**	DESKTOP-9792RPL\GCMS
**Report Generation Time**	3/25/2025 3:57:34 PM	**Report Generator Name**	DESKTOP-9792RPL\GCMS
**Calibration Last Update**	3/25/2025 3:57:01 PM	**Batch State**	Processed
**Analyze Quant Version**	10.2	**Report Quant Version**	10.2
**Acq. Date-Time**	2/11/2025 7:31:19 PM	**Data File**	11_2_25_DAY3_7510_CBD_PEG400_1.D
**Type**	Sample	**Name**	11_2_25_DAY3_7510_CBD_PEG400_1
**Dil.**	1	**Acq. Method File**	CannabinoidsAgilent 29_05

Sample Chromatogram

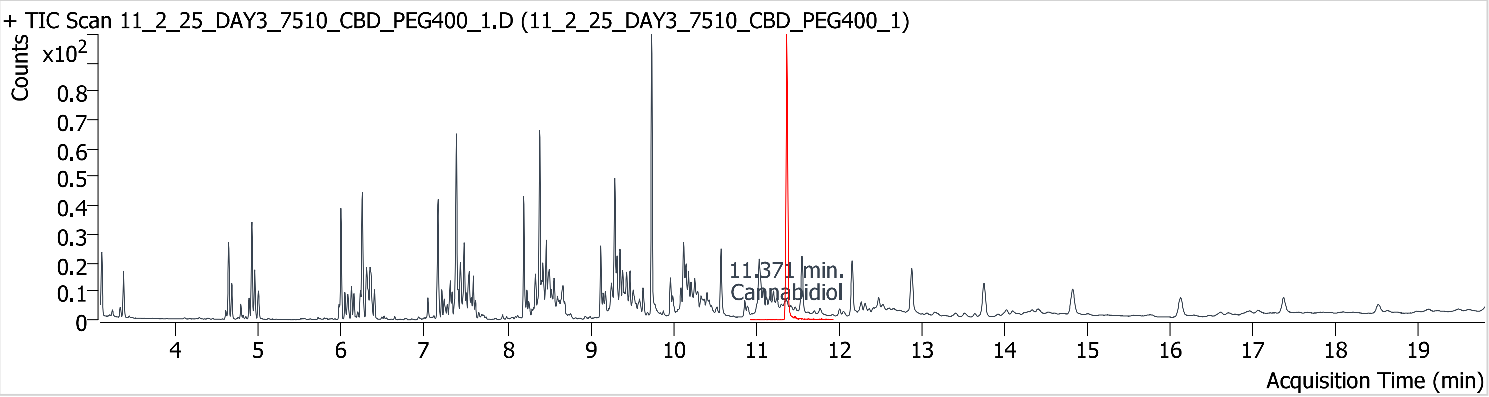

Name	RT	Transition	Resp.	Height	Final Conc.	Units
Cannabidiol	11.371	231.0	137009	91795.05	11.6723	uM

Cannabidiol

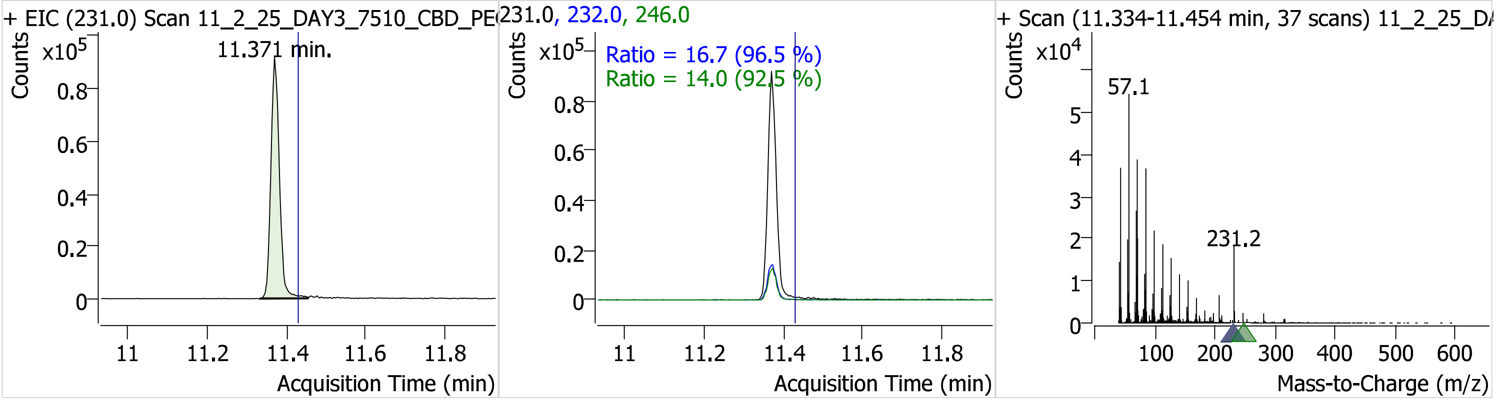

Supplement: Supplementary file 1 [file pharmaceutics-17-00463-s001.zip › pharmaceutics-3548672-supplementary/Supplementary File S2 Chromatogram samples.pdf]
